# Supplementary figures and images for: NAD+ prevents septic shock-induced death by non-canonical inflammasome blockade and IL-10 cytokine production in macrophages (part 1 of 2)
Source: eLife. 2024 Feb 19;12:RP88686. doi: 10.7554/eLife.88686 (PMC10942599; doi:10.7554/eLife.88686)

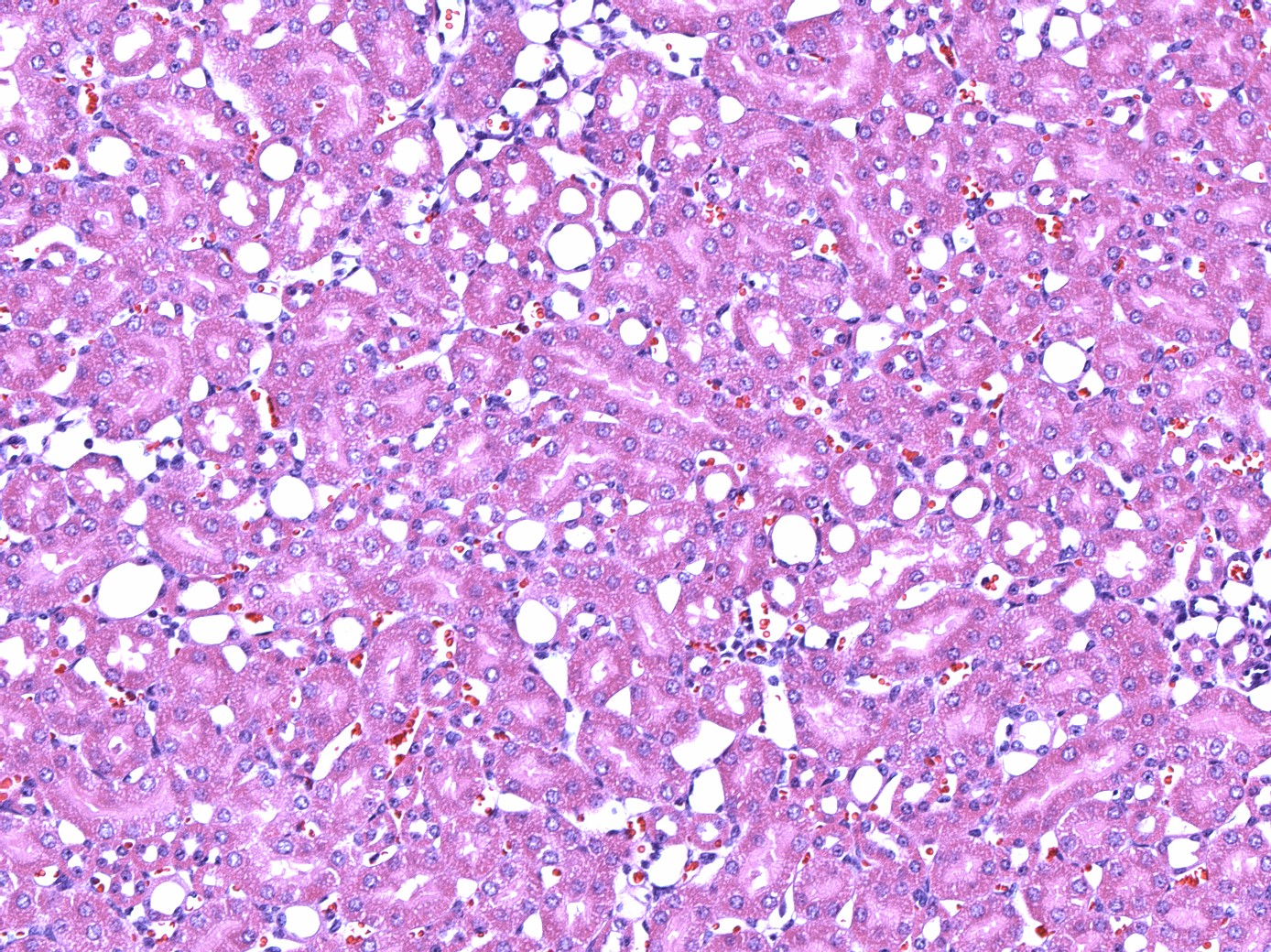

Supplement: Figure 1—source data 4. [file elife-88686-fig1-data4.zip › Kidney NAD+/Kidney_H&E_20x_NAD+_1.jpg]

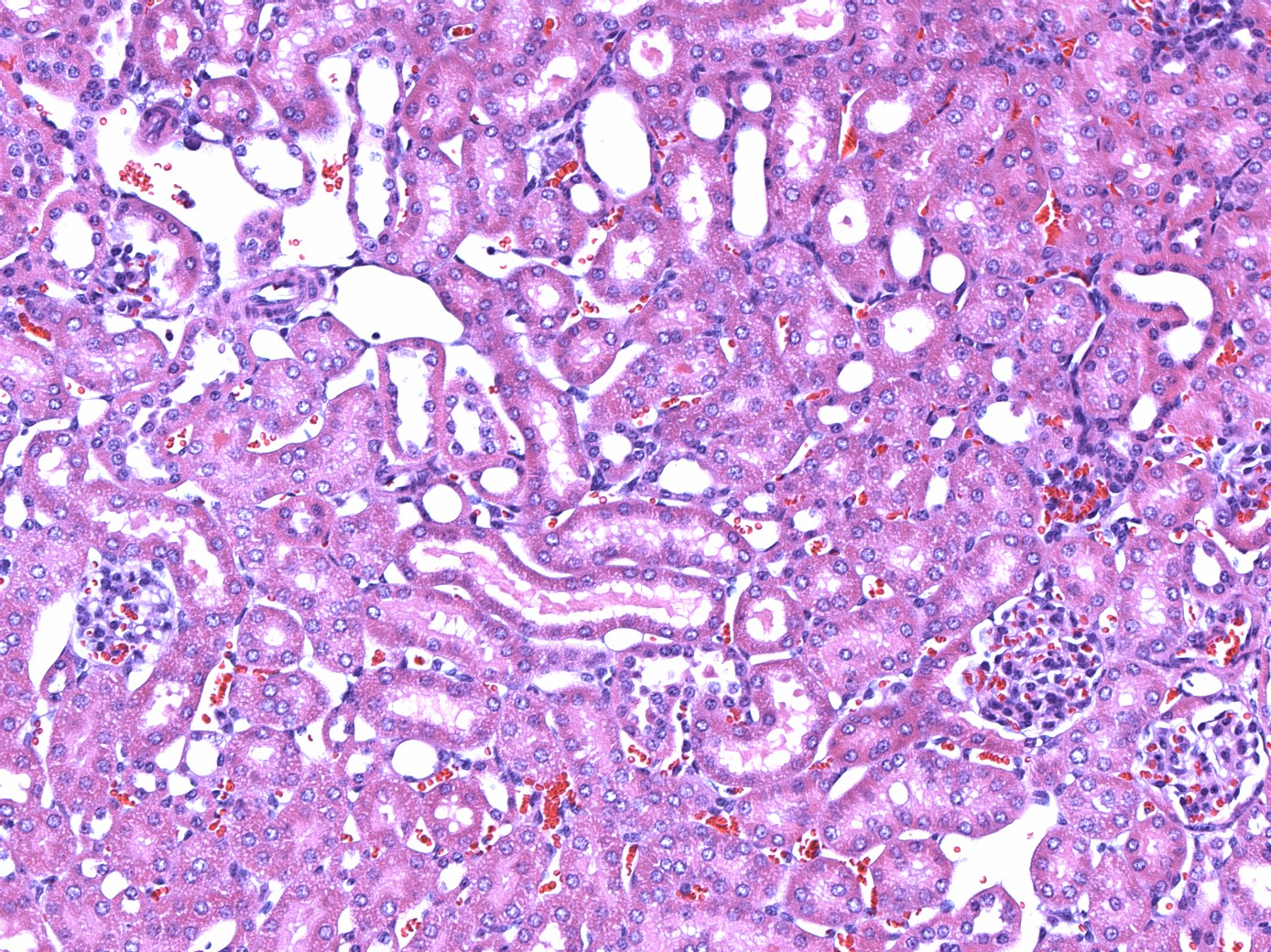

Supplement: Figure 1—source data 4. [file elife-88686-fig1-data4.zip › Kidney NAD+/Kidney_H&E_20x_NAD+_3.jpg]

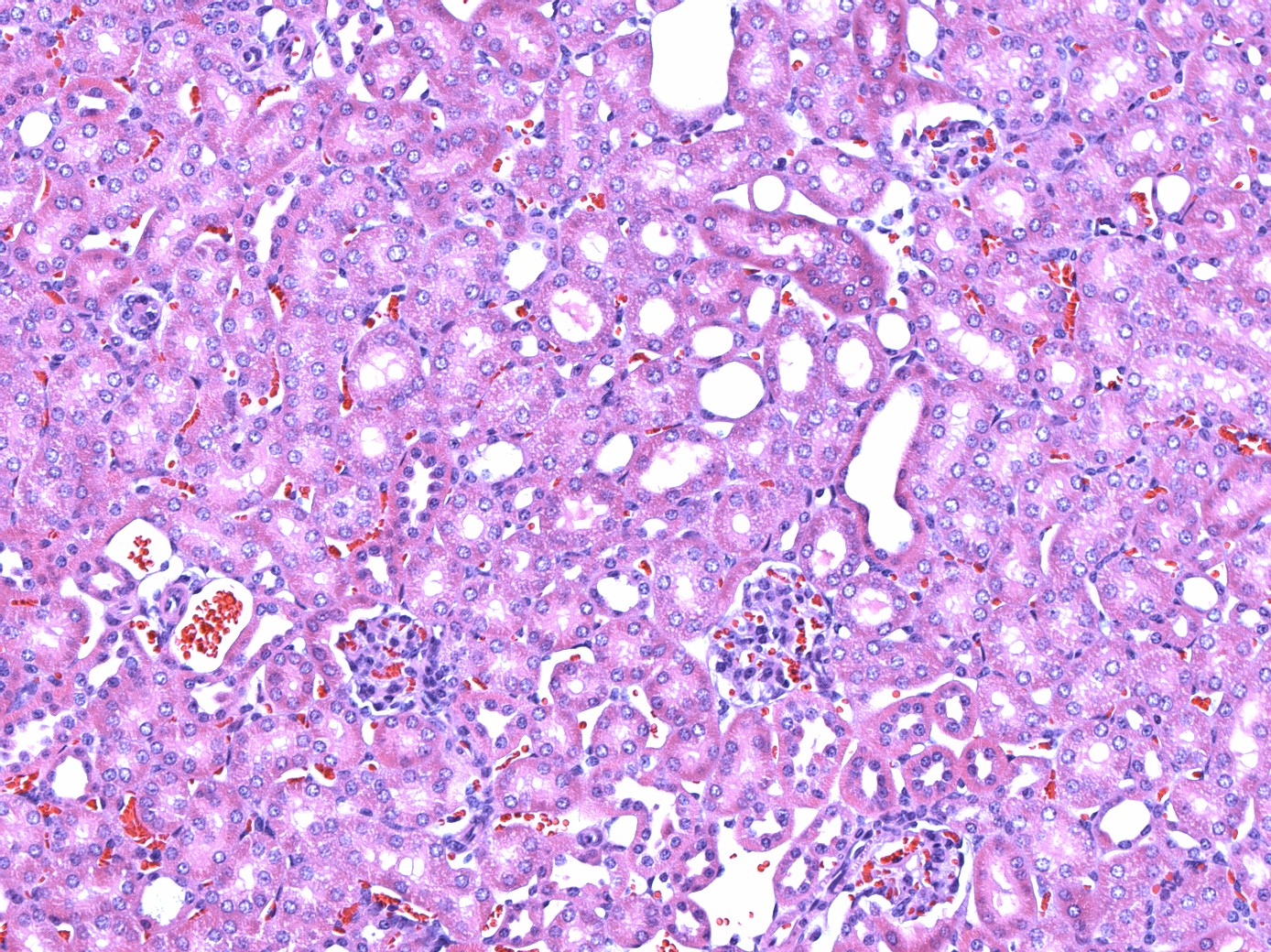

Supplement: Figure 1—source data 4. [file elife-88686-fig1-data4.zip › Kidney NAD+/Kidney_H&E_20x_NAD+_4.jpg]

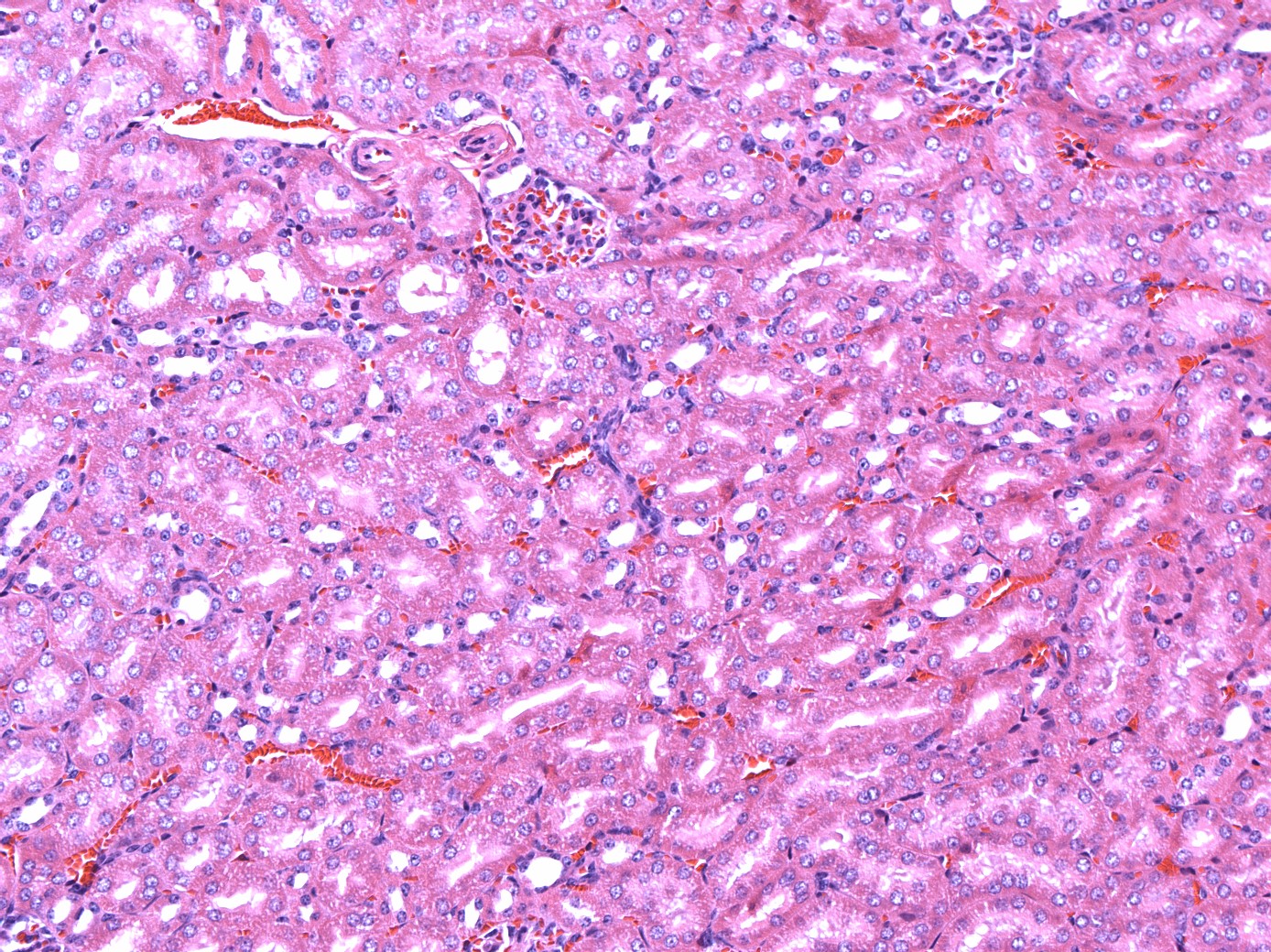

Supplement: Figure 1—source data 4. [file elife-88686-fig1-data4.zip › Kidney NAD+/Kidney_H&E_20x_NAD+_5.jpg]

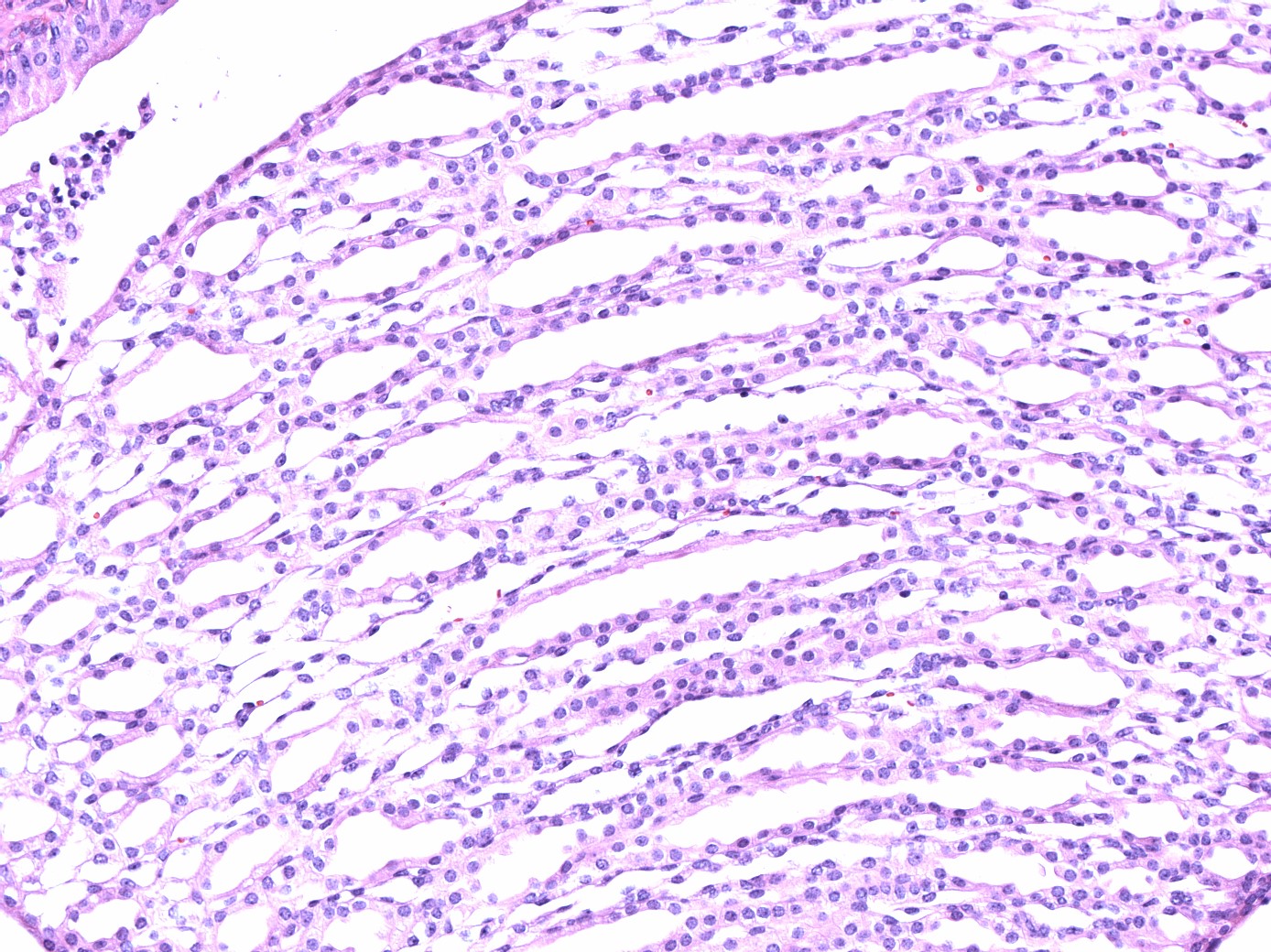

Supplement: Figure 1—source data 4. [file elife-88686-fig1-data4.zip › Kidney NAD+/Kidney_H&E_20x_NAD+_6.jpg]

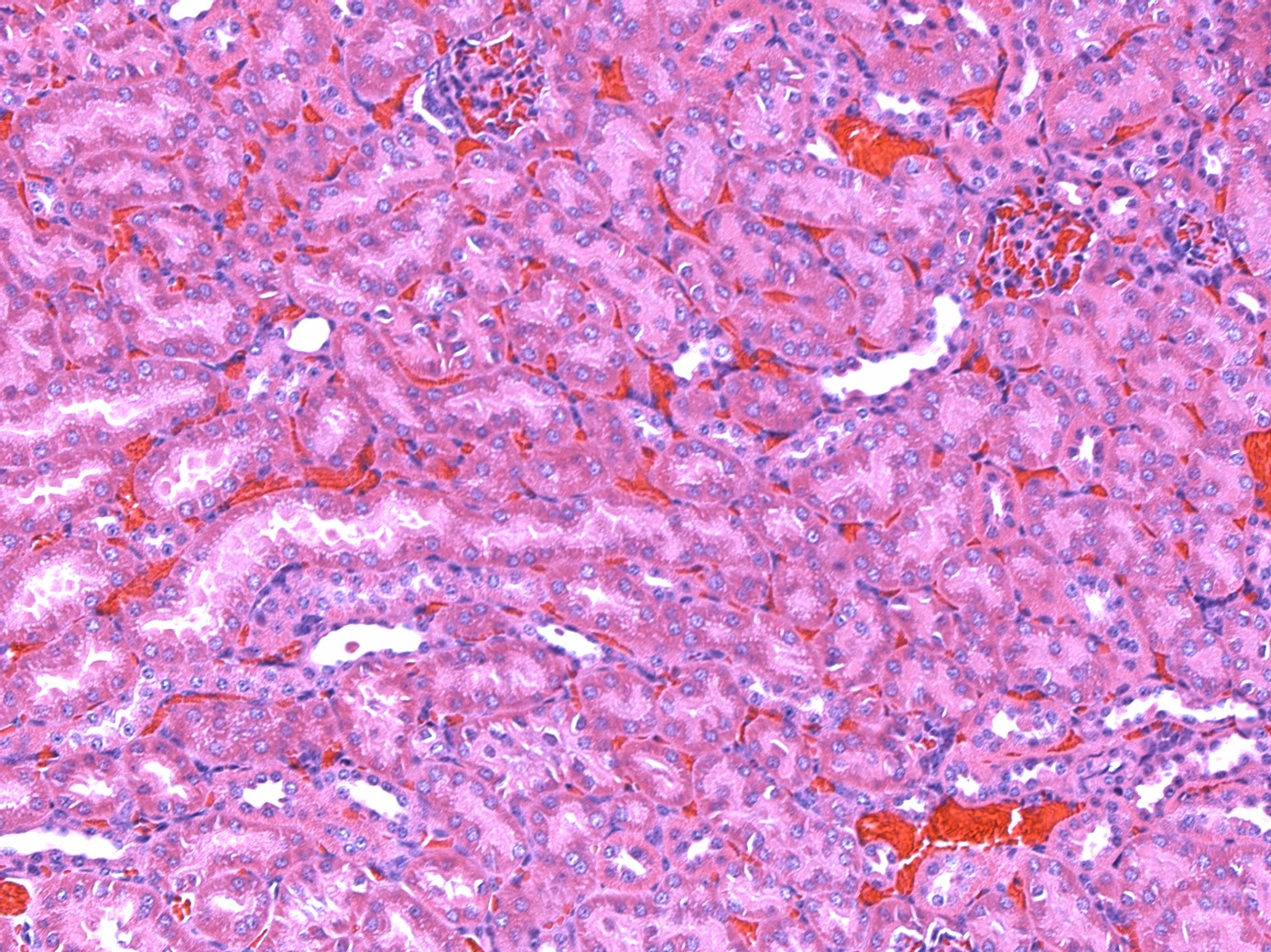

Supplement: Figure 1—source data 4. [file elife-88686-fig1-data4.zip › Kidney PBS/Kidney_H&E_20x_PBS_1.jpg]

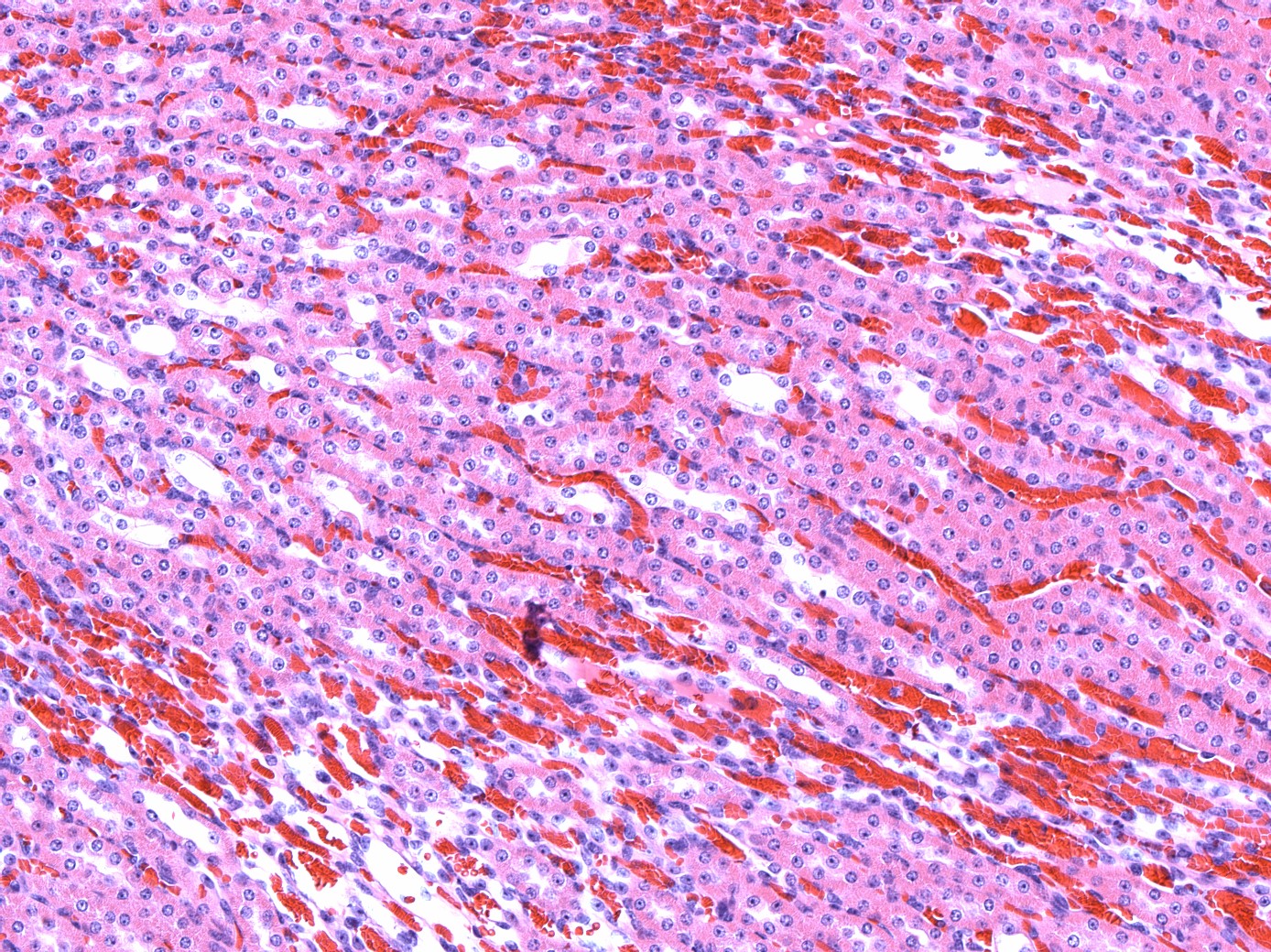

Supplement: Figure 1—source data 4. [file elife-88686-fig1-data4.zip › Kidney PBS/Kidney_H&E_20x_PBS_2.jpg]

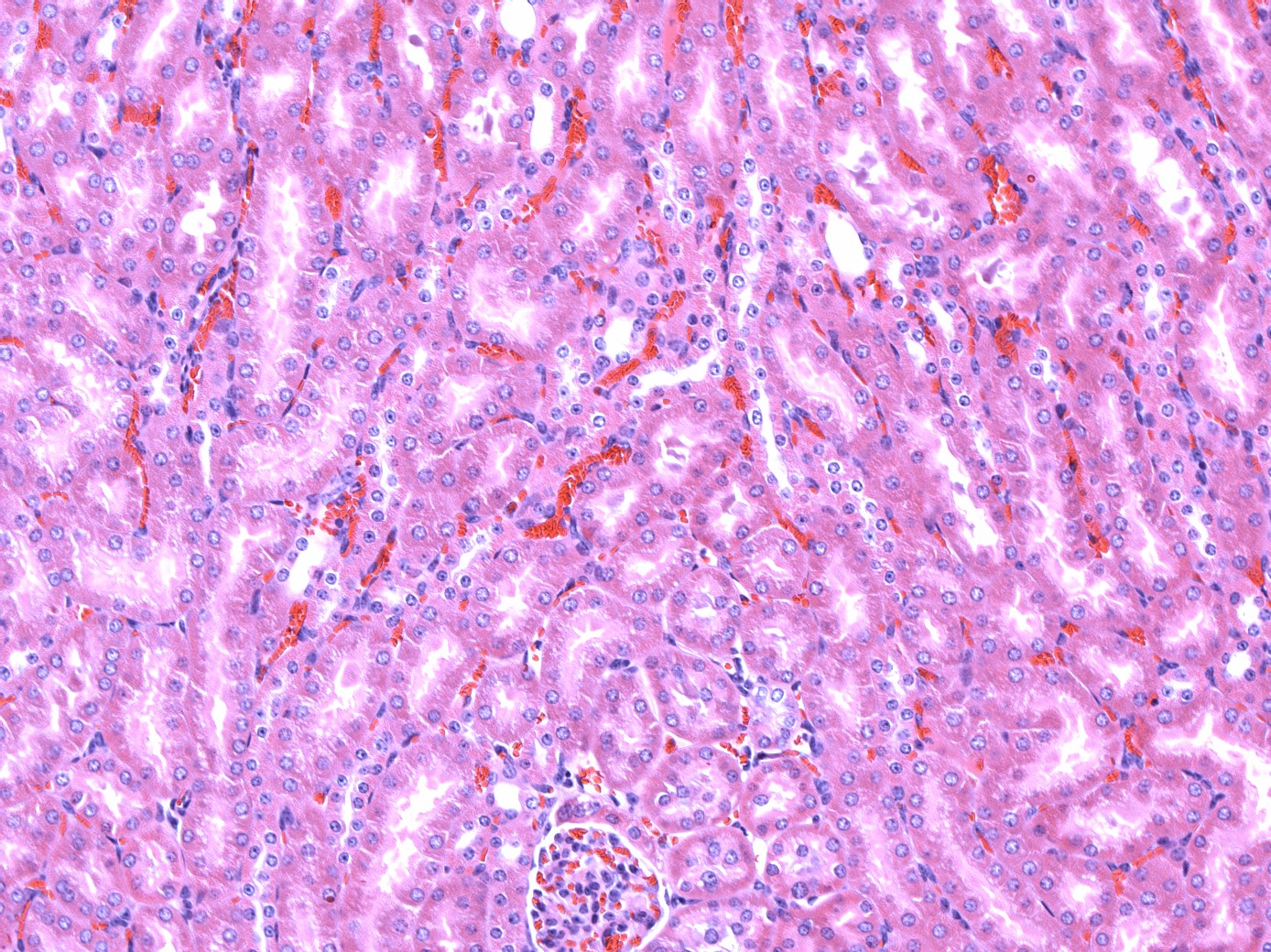

Supplement: Figure 1—source data 4. [file elife-88686-fig1-data4.zip › Kidney PBS/Kidney_H&E_20x_PBS_3.jpg]

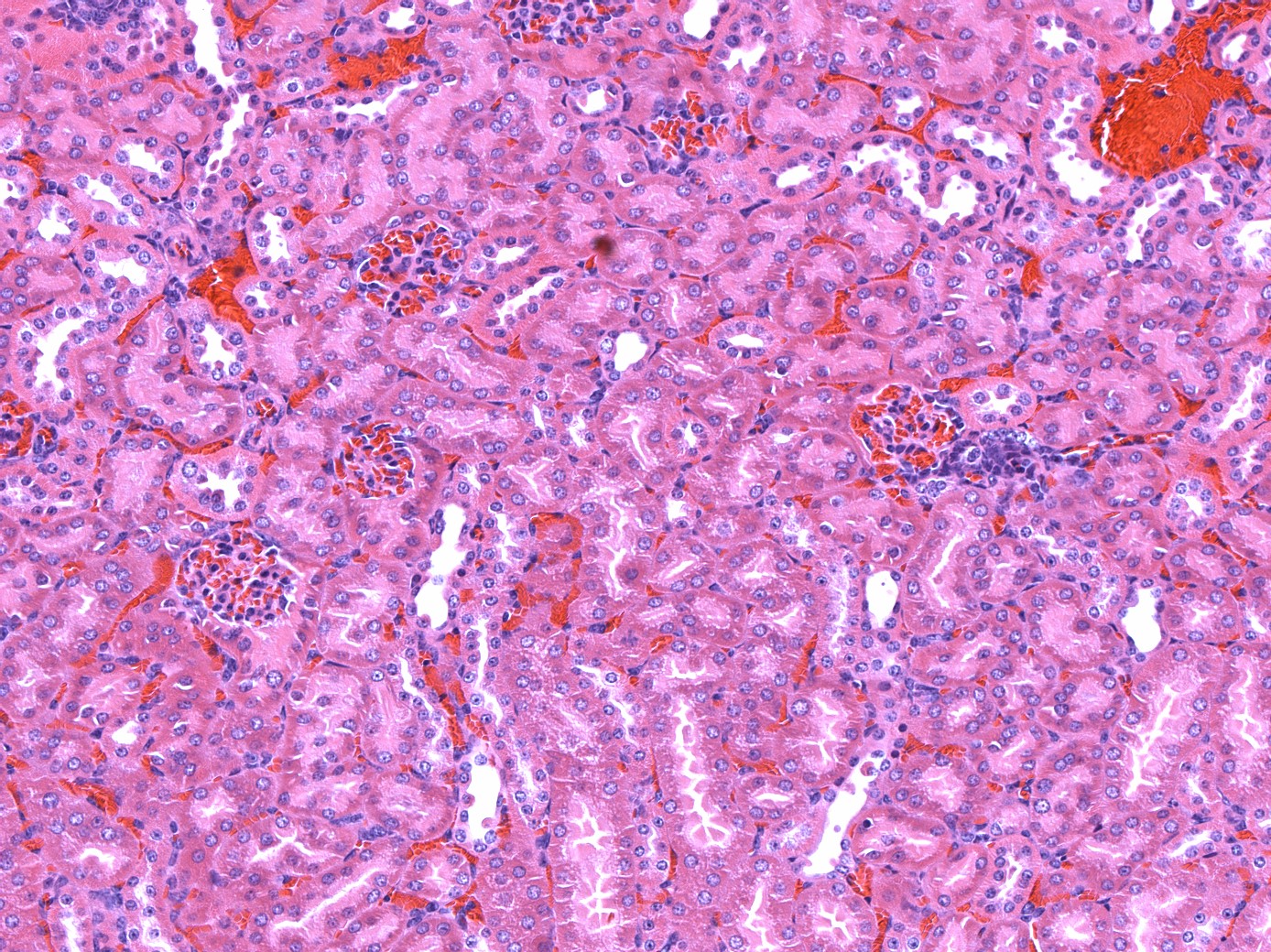

Supplement: Figure 1—source data 4. [file elife-88686-fig1-data4.zip › Kidney PBS/Kidney_H&E_20x_PBS_4.jpg]

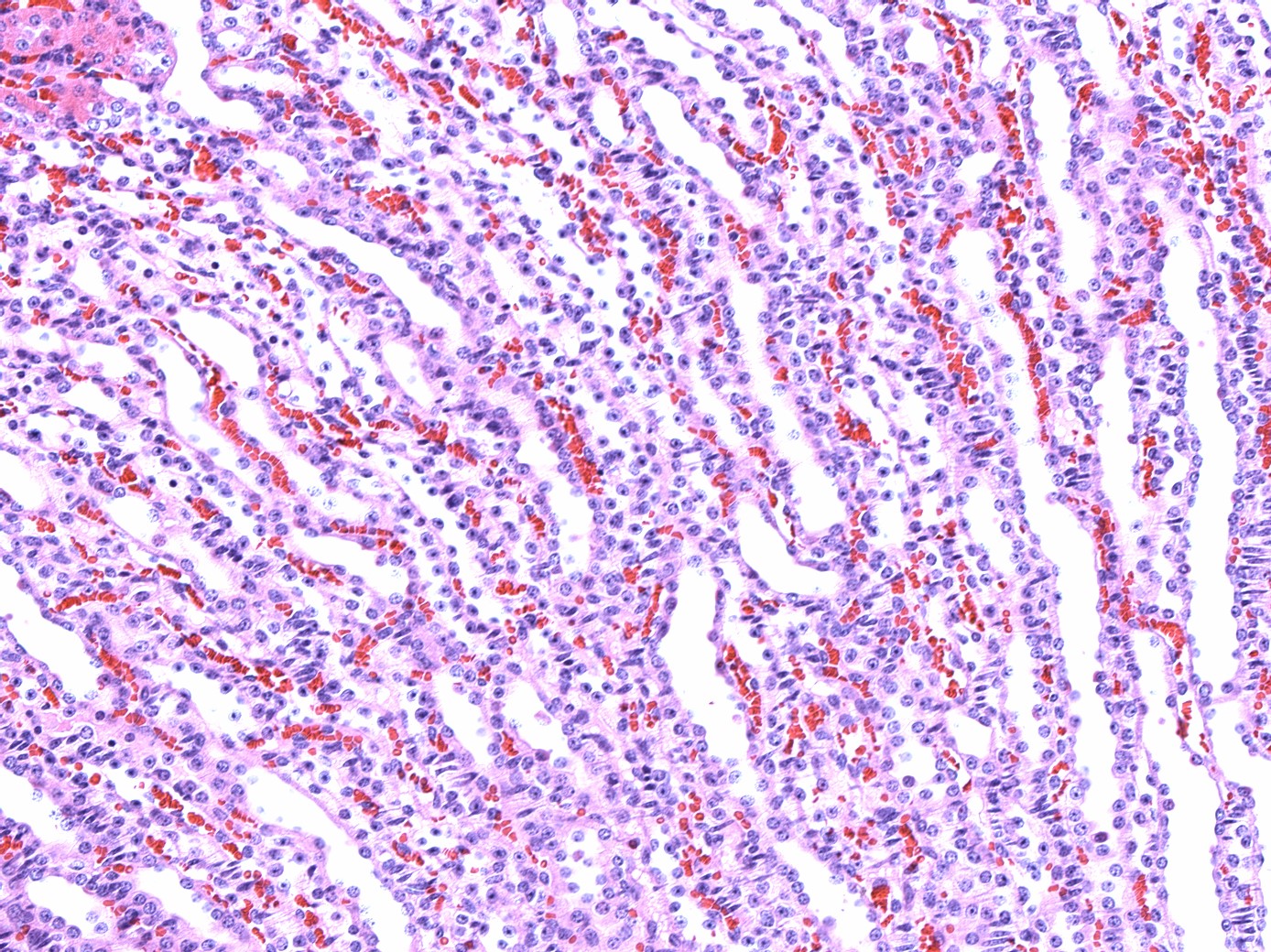

Supplement: Figure 1—source data 4. [file elife-88686-fig1-data4.zip › Kidney PBS/Kidney_H&E_20x_PBS_5.jpg]

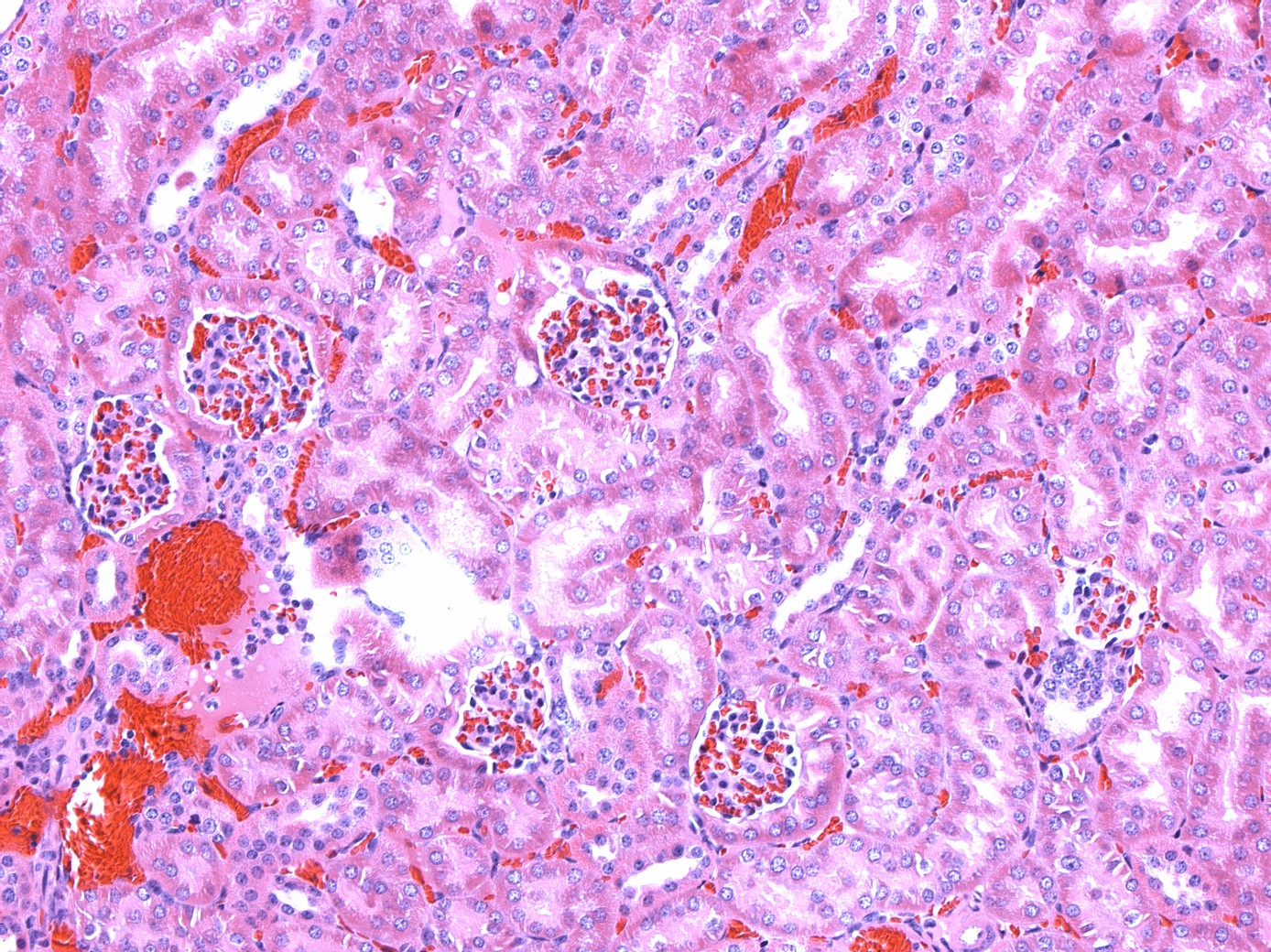

Supplement: Figure 1—source data 4. [file elife-88686-fig1-data4.zip › Kidney PBS/Kidney_H&E_20x_PBS_6.jpg]

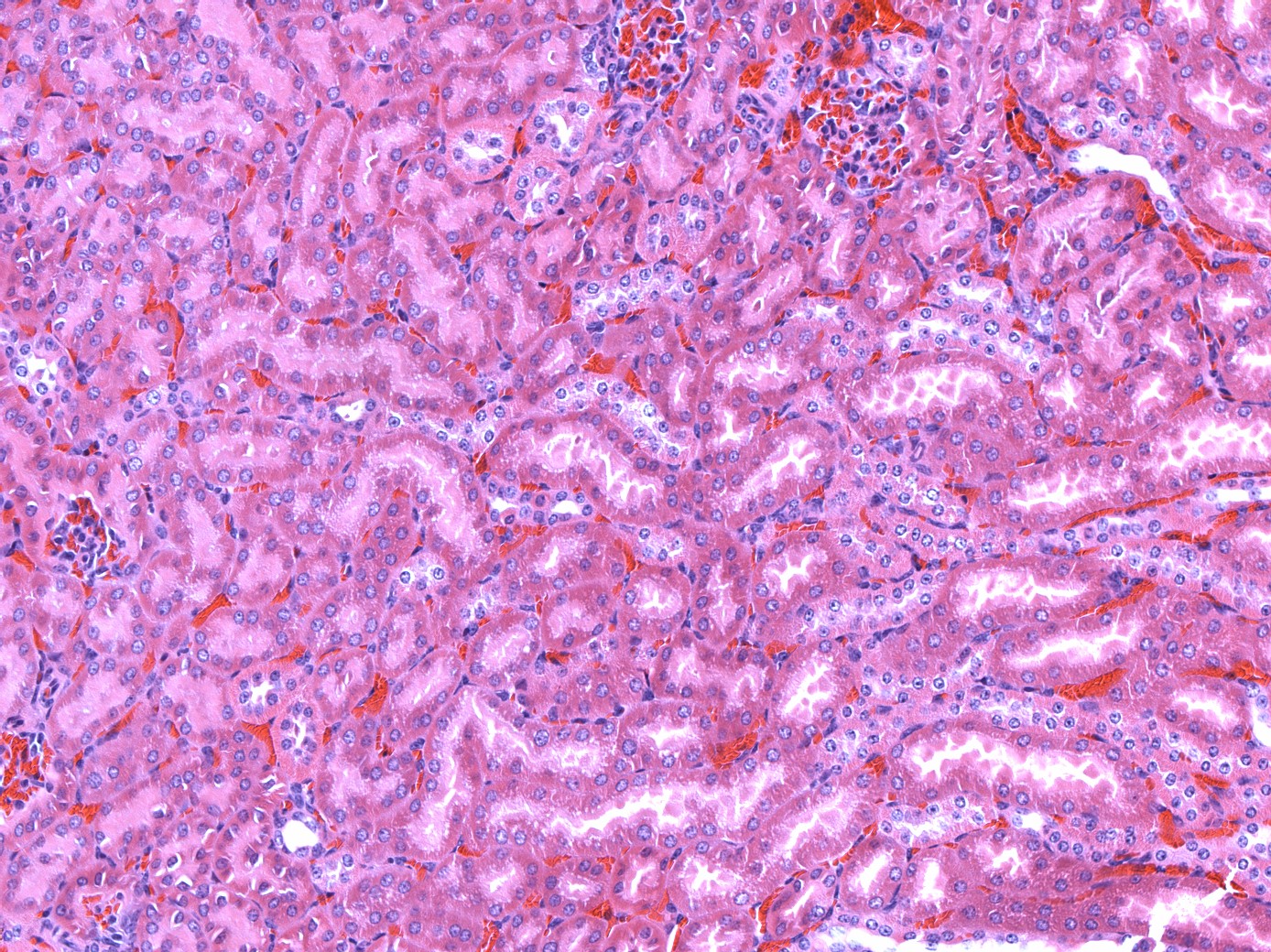

Supplement: Figure 1—source data 4. [file elife-88686-fig1-data4.zip › Kidney PBS/Kidney_H&E_20x_PBS_7.jpg]

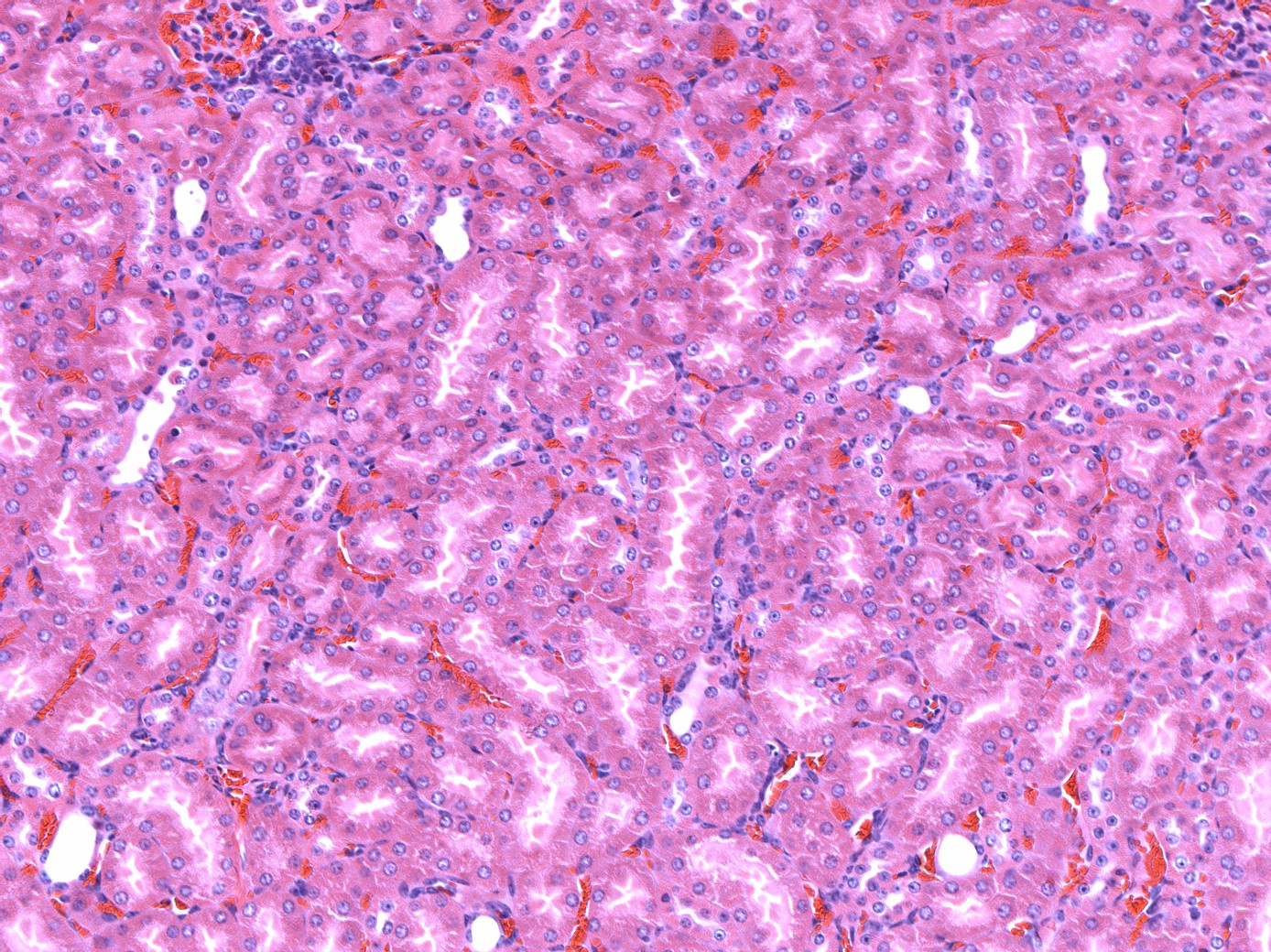

Supplement: Figure 1—source data 4. [file elife-88686-fig1-data4.zip › Kidney PBS/Kidney_H&E_20x_PBS_8.jpg]

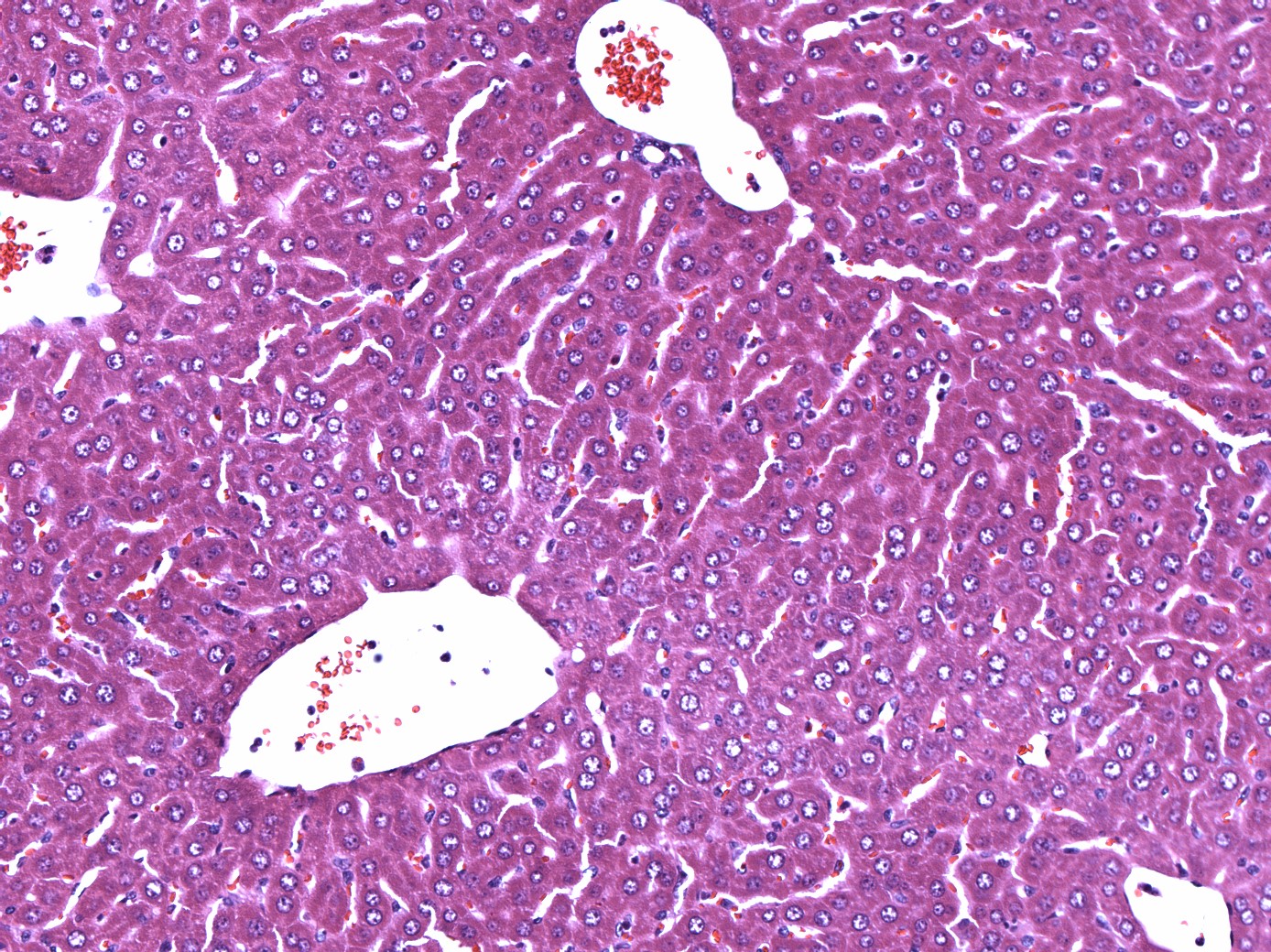

Supplement: Figure 1—source data 4. [file elife-88686-fig1-data4.zip › Liver NAD+/Liver_H&E_20x_NAD_1.jpg]

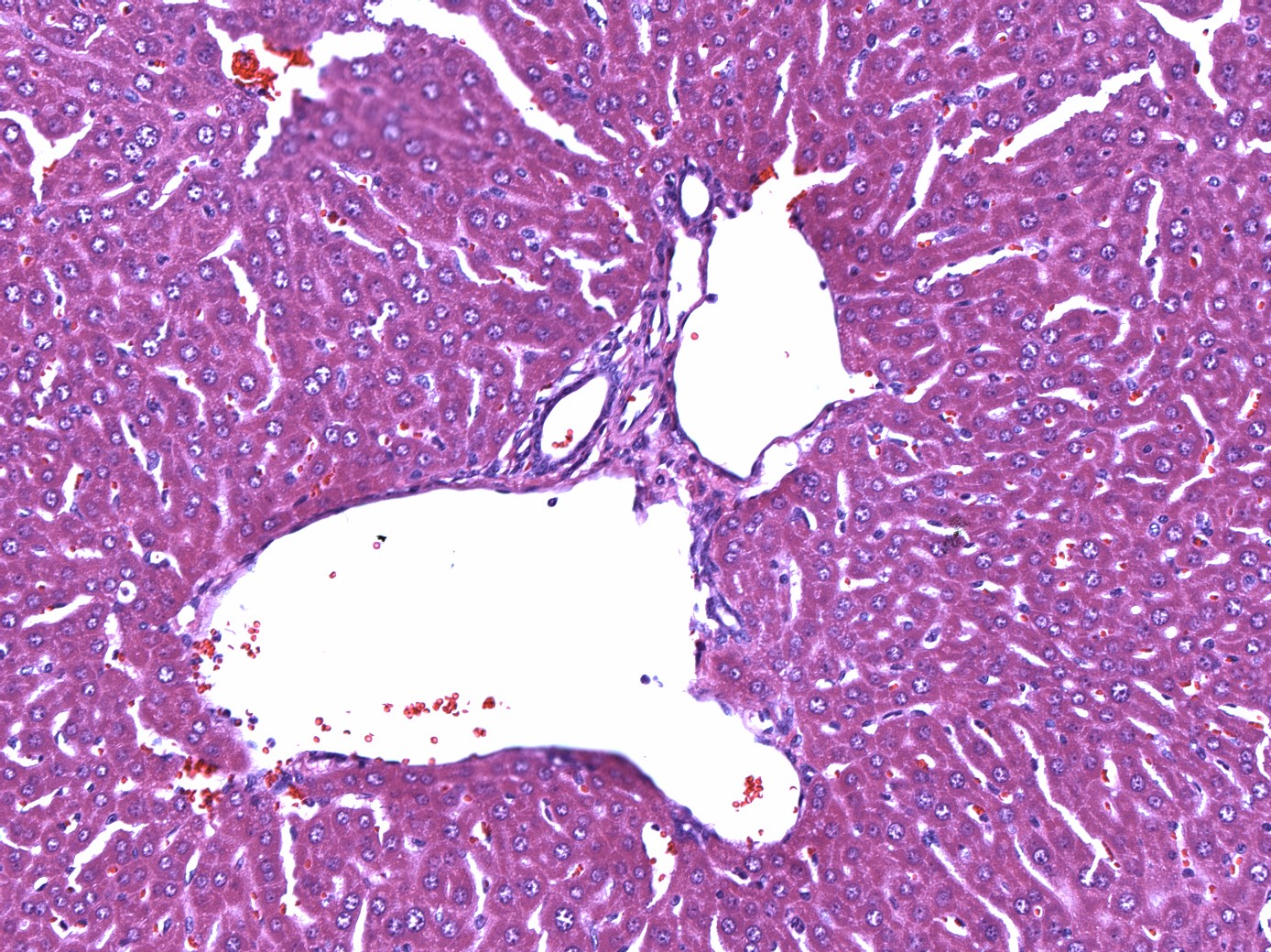

Supplement: Figure 1—source data 4. [file elife-88686-fig1-data4.zip › Liver NAD+/Liver_H&E_20x_NAD_2.jpg]

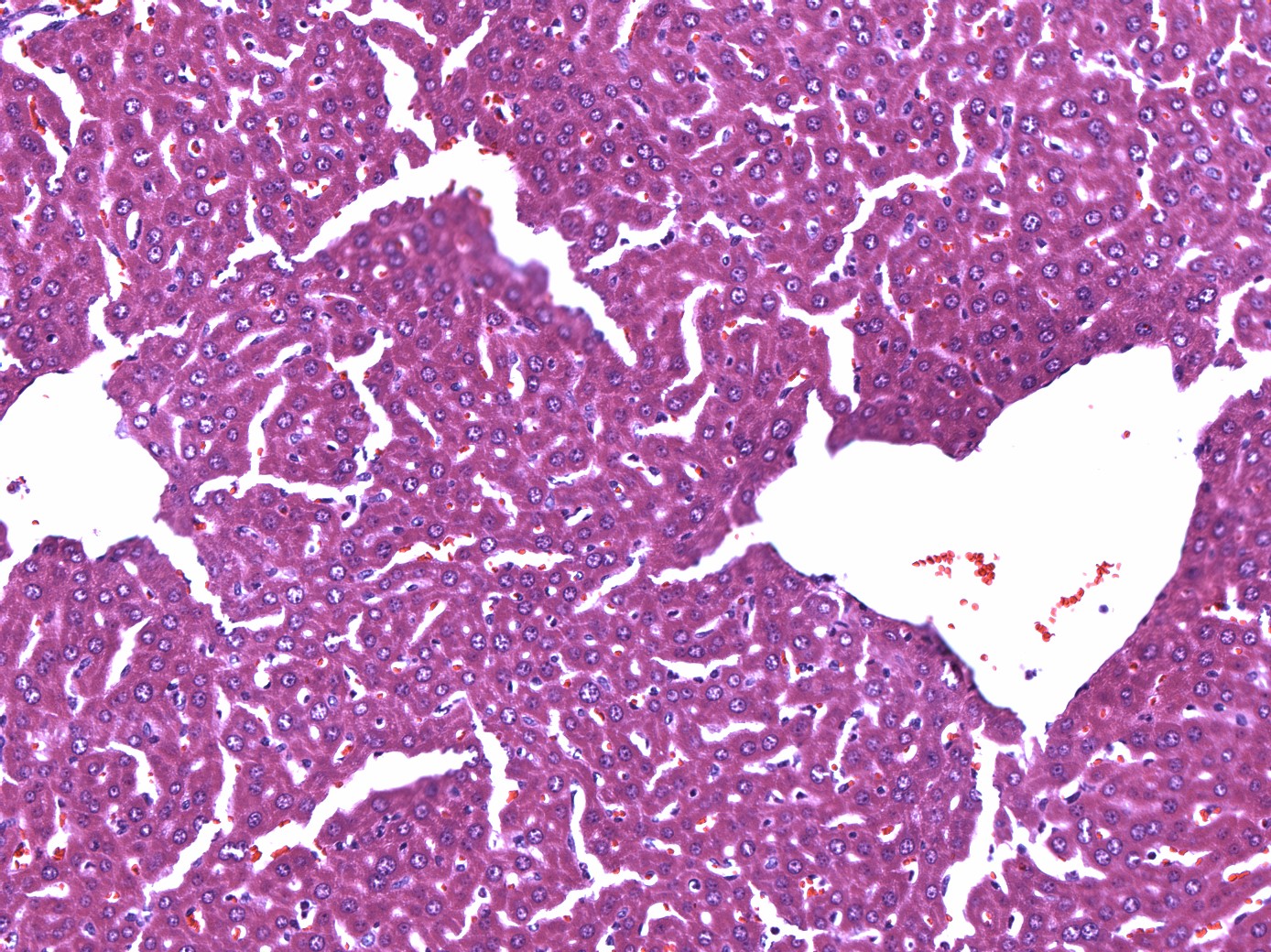

Supplement: Figure 1—source data 4. [file elife-88686-fig1-data4.zip › Liver NAD+/Liver_H&E_20x_NAD_3.jpg]

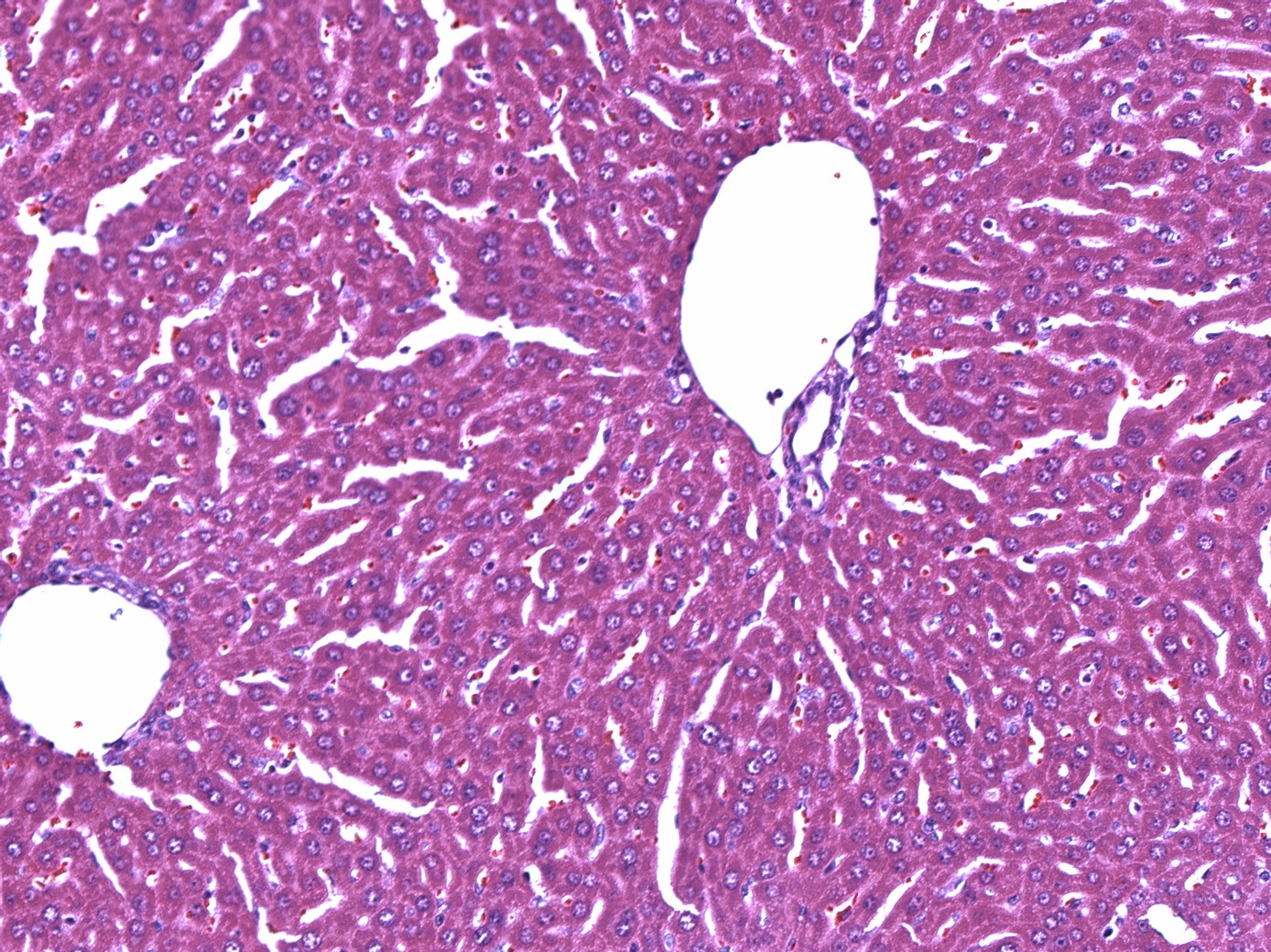

Supplement: Figure 1—source data 4. [file elife-88686-fig1-data4.zip › Liver NAD+/Liver_H&E_20x_NAD_4.jpg]

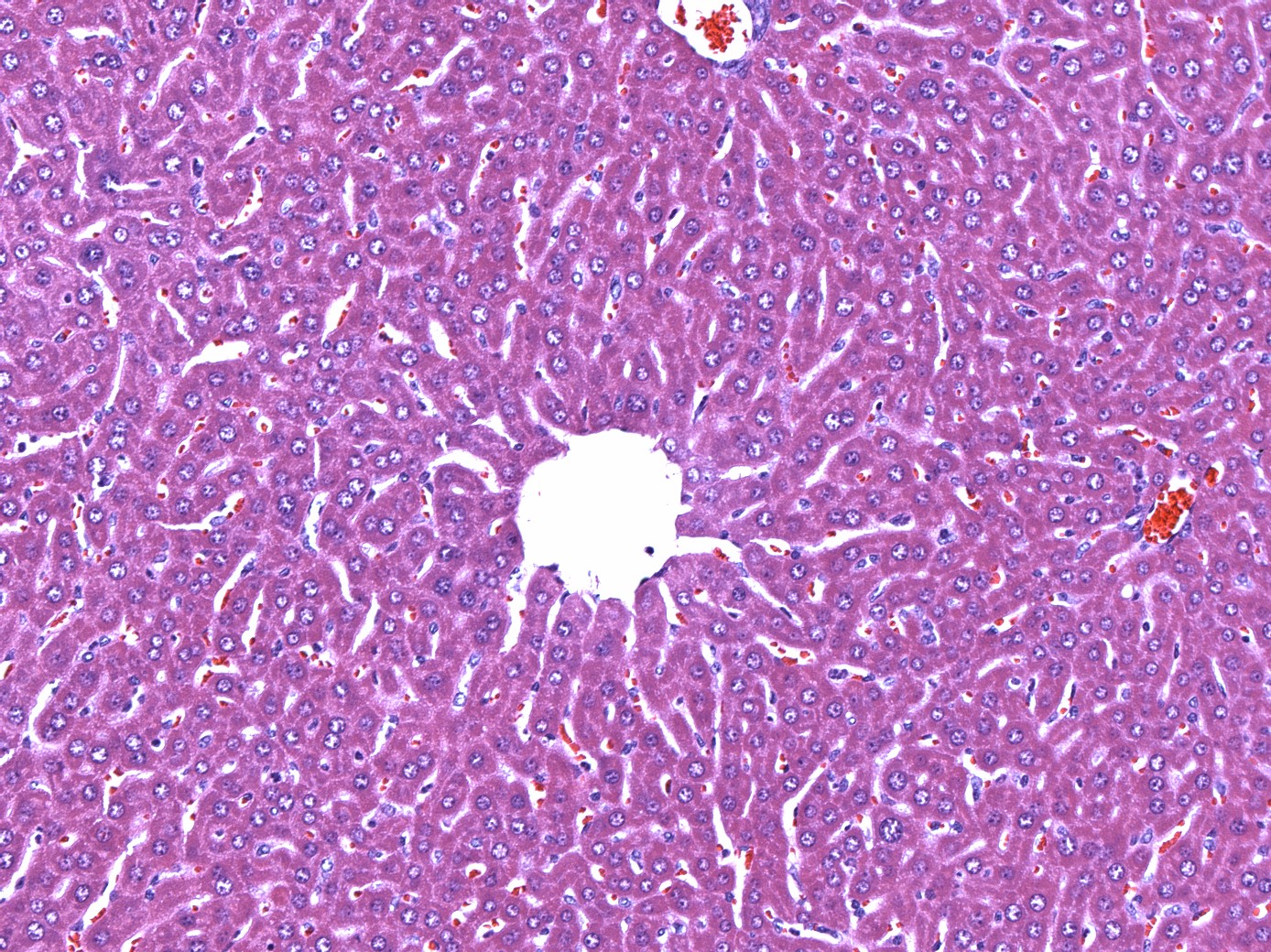

Supplement: Figure 1—source data 4. [file elife-88686-fig1-data4.zip › Liver NAD+/Liver_H&E_20x_NAD_5.jpg]

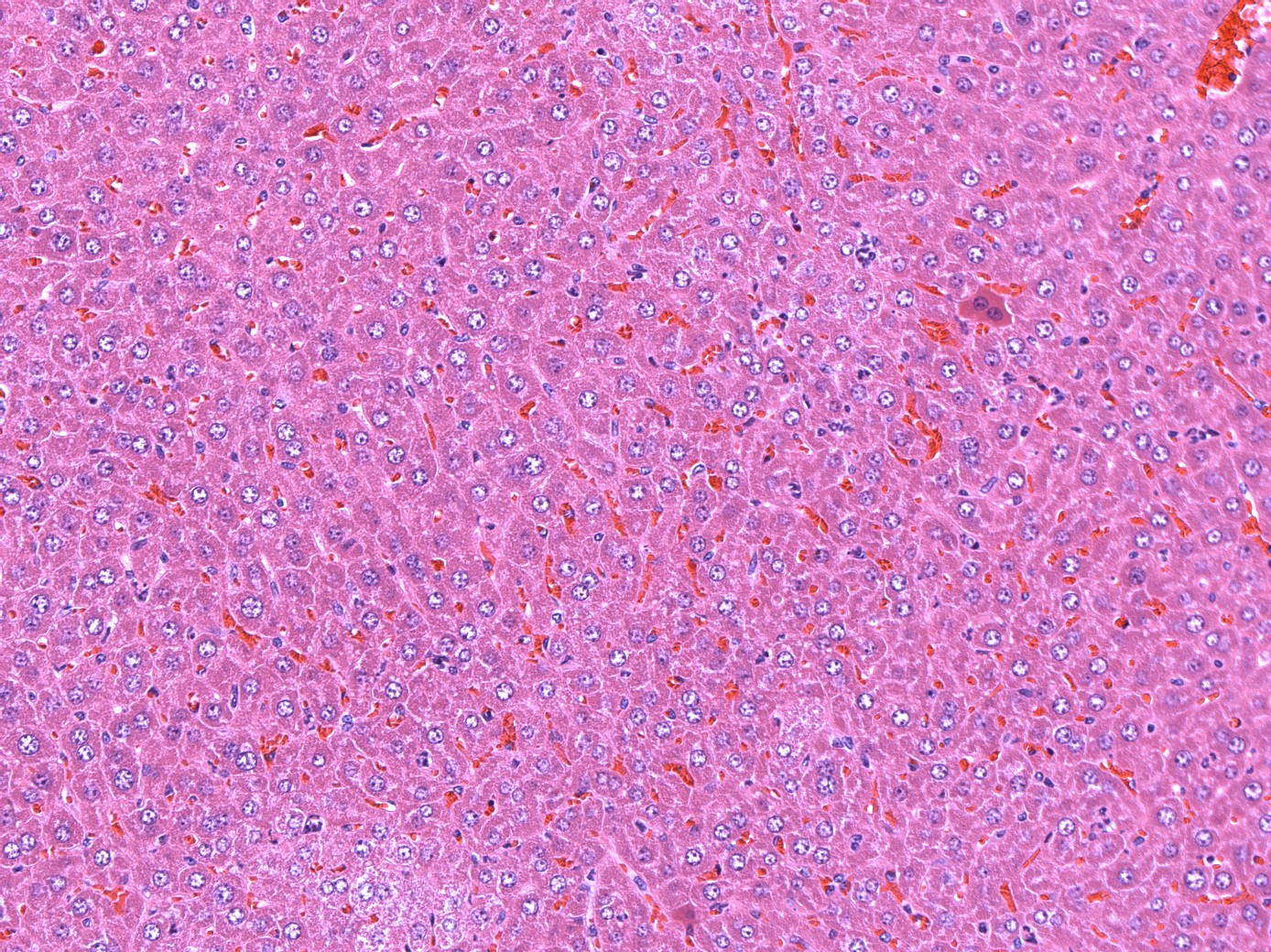

Supplement: Figure 1—source data 4. [file elife-88686-fig1-data4.zip › Liver PBS/Liver_H&E_20x_PBS_1.jpg]

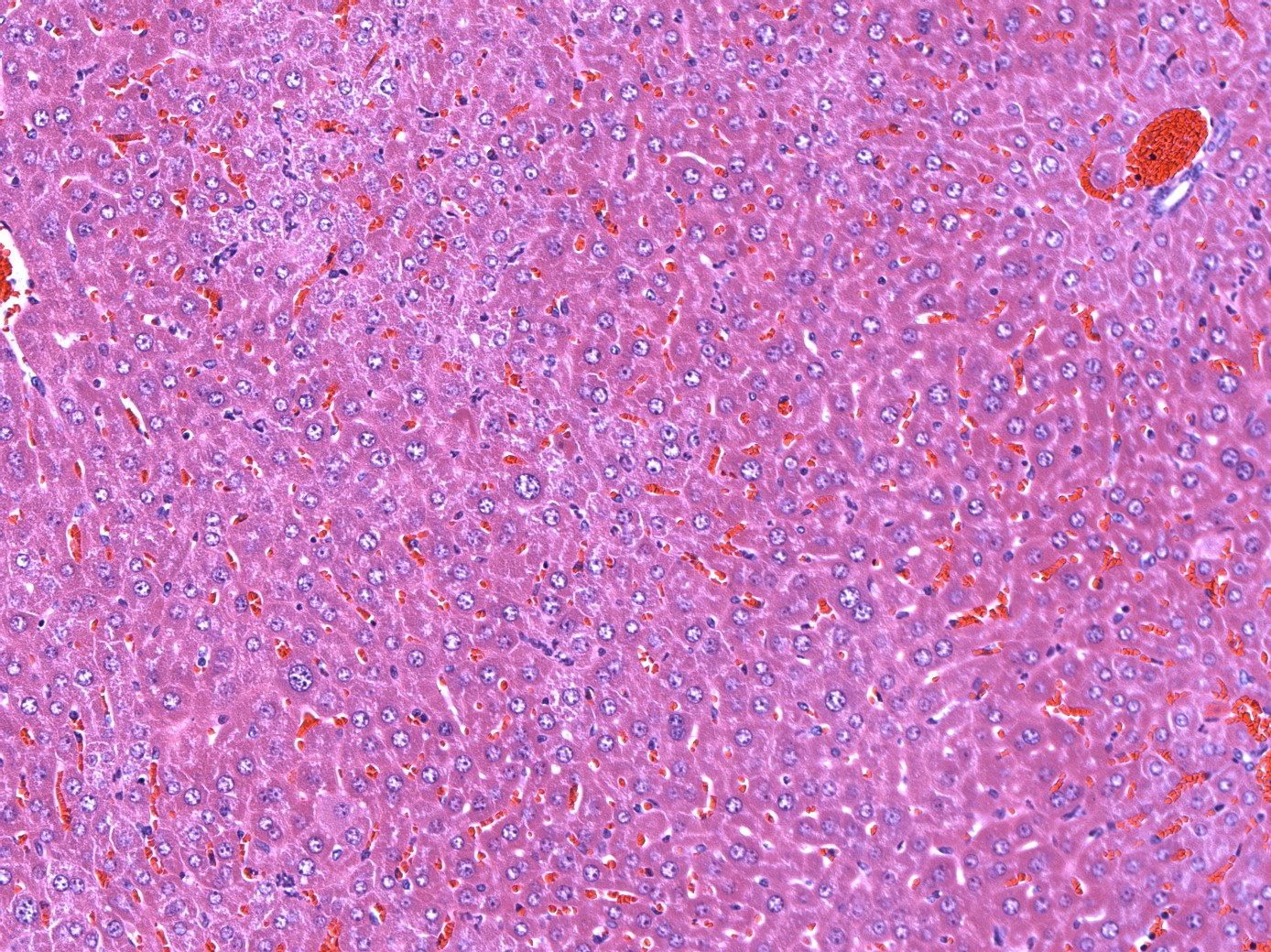

Supplement: Figure 1—source data 4. [file elife-88686-fig1-data4.zip › Liver PBS/Liver_H&E_20x_PBS_2.jpg]

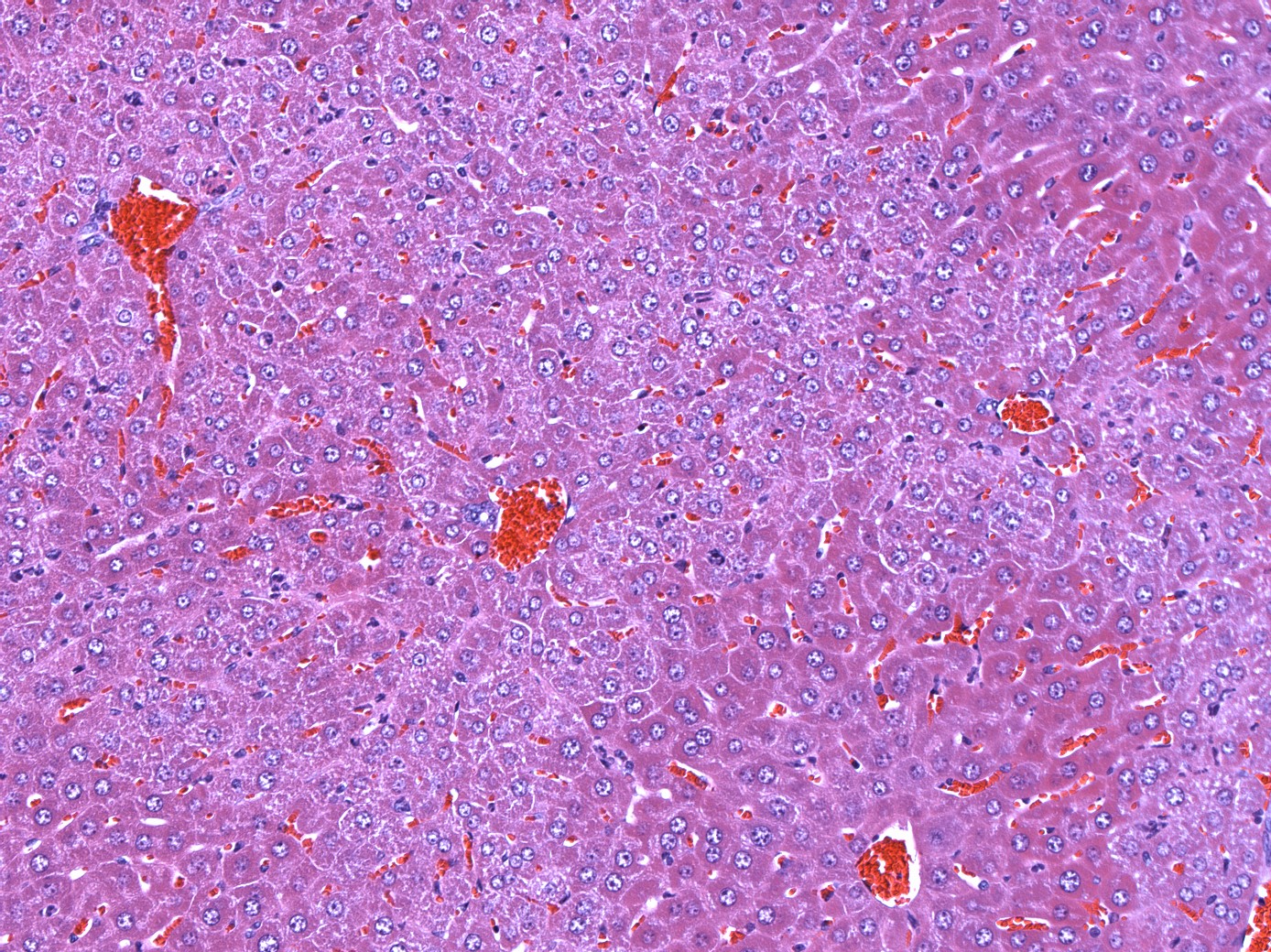

Supplement: Figure 1—source data 4. [file elife-88686-fig1-data4.zip › Liver PBS/Liver_H&E_20x_PBS_3.jpg]

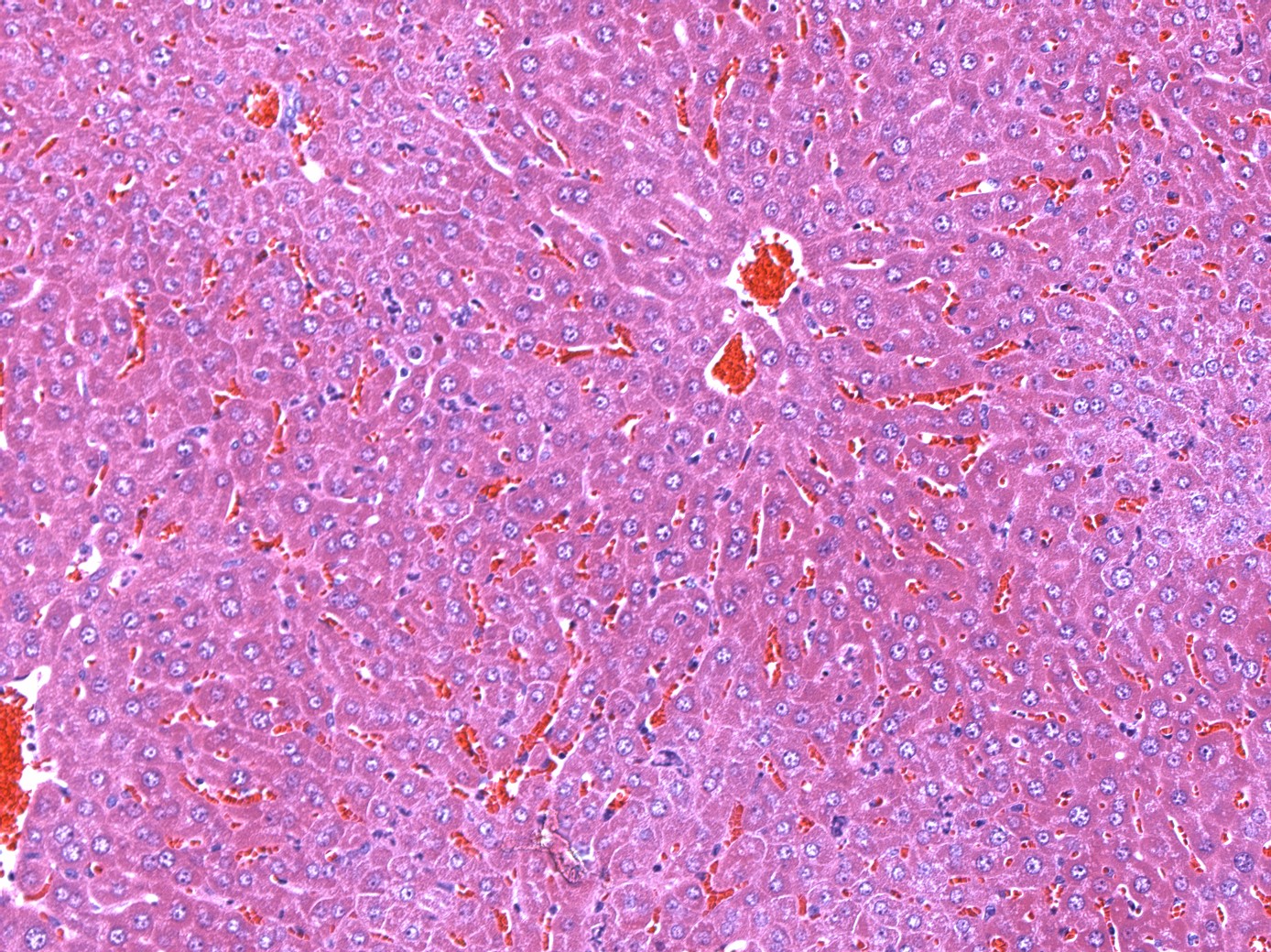

Supplement: Figure 1—source data 4. [file elife-88686-fig1-data4.zip › Liver PBS/Liver_H&E_20x_PBS_4.jpg]

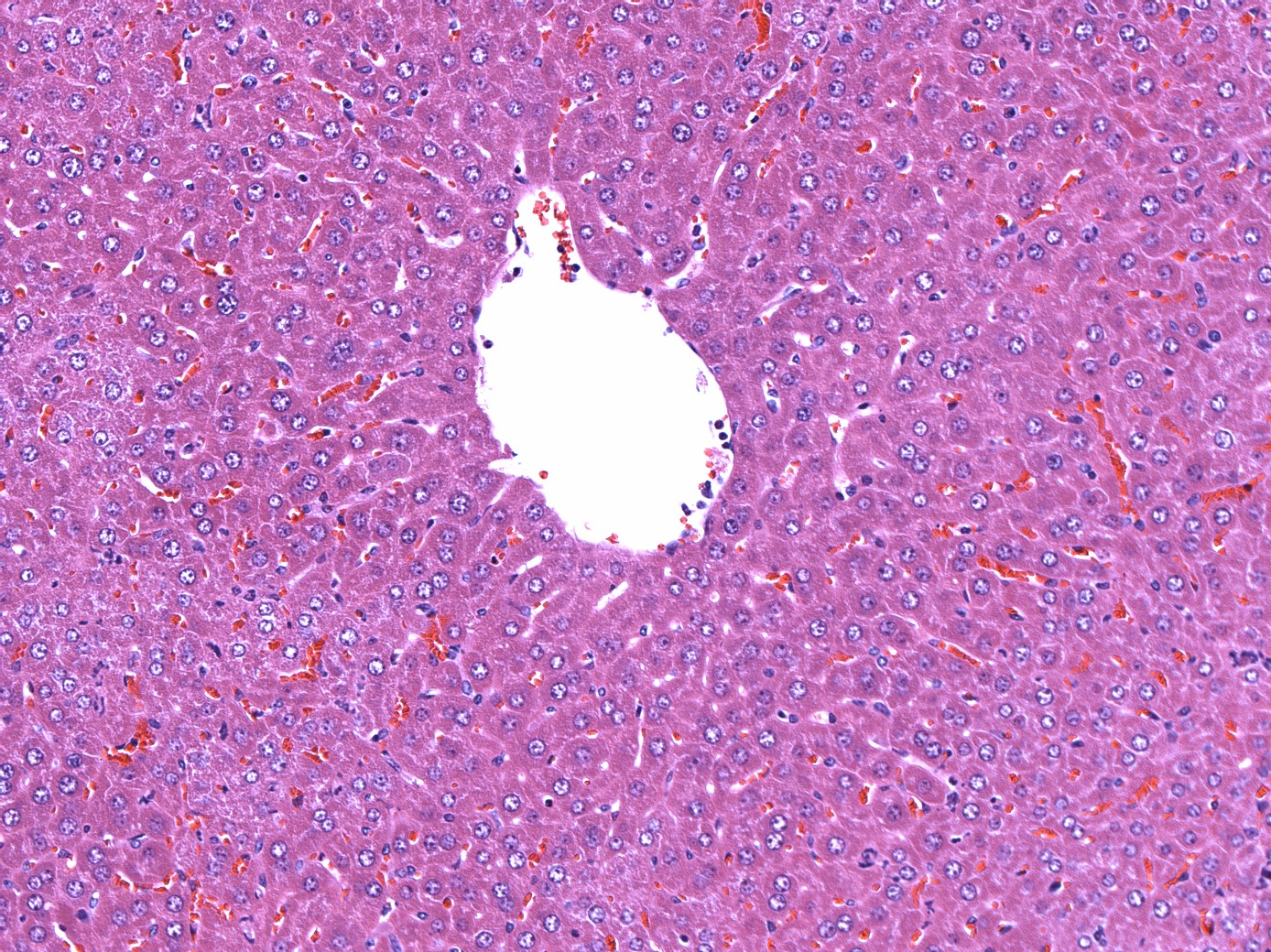

Supplement: Figure 1—source data 4. [file elife-88686-fig1-data4.zip › Liver PBS/Liver_H&E_20x_PBS_5.jpg]

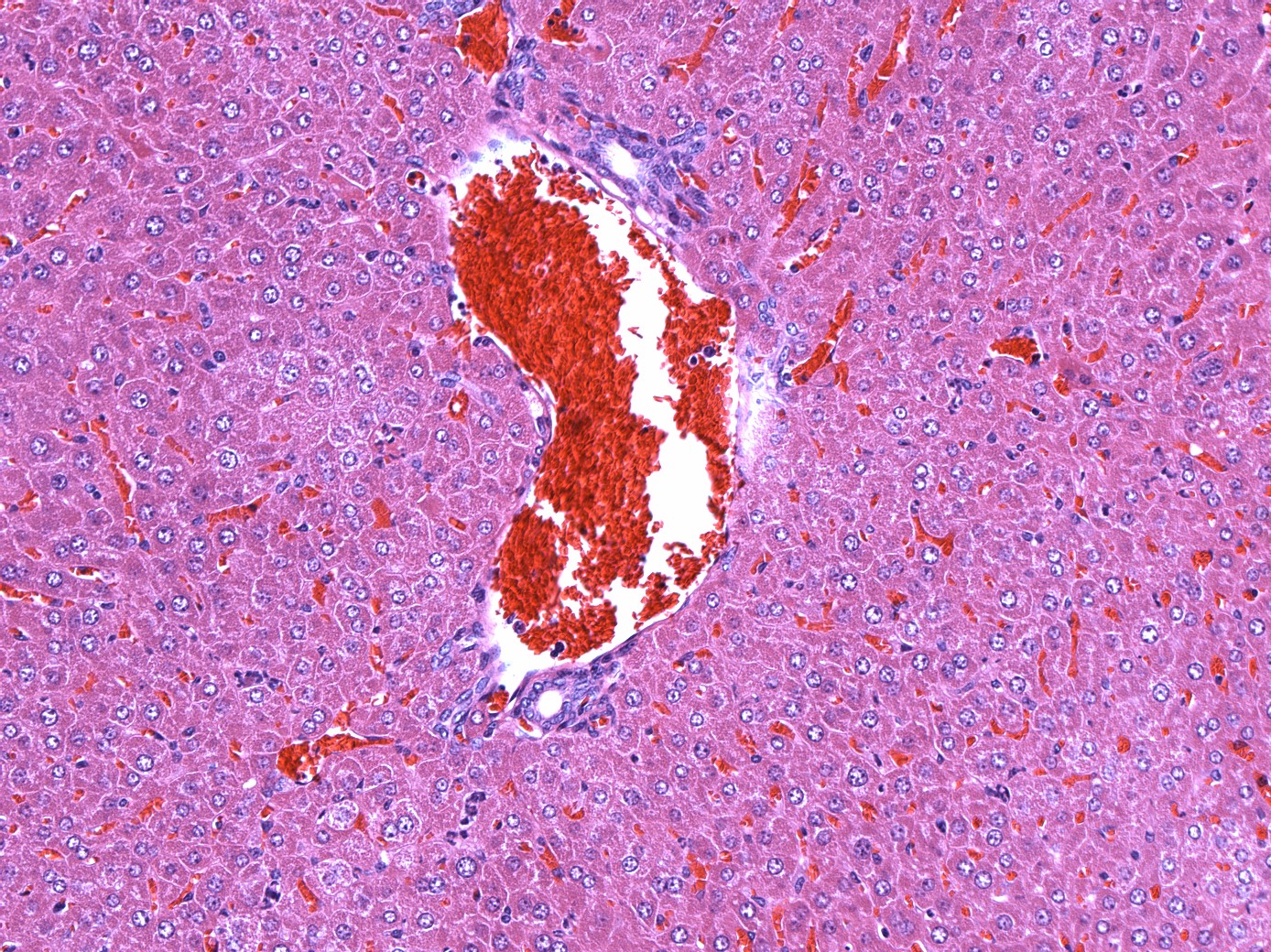

Supplement: Figure 1—source data 4. [file elife-88686-fig1-data4.zip › Liver PBS/Liver_H&E_20x_PBS_6.jpg]

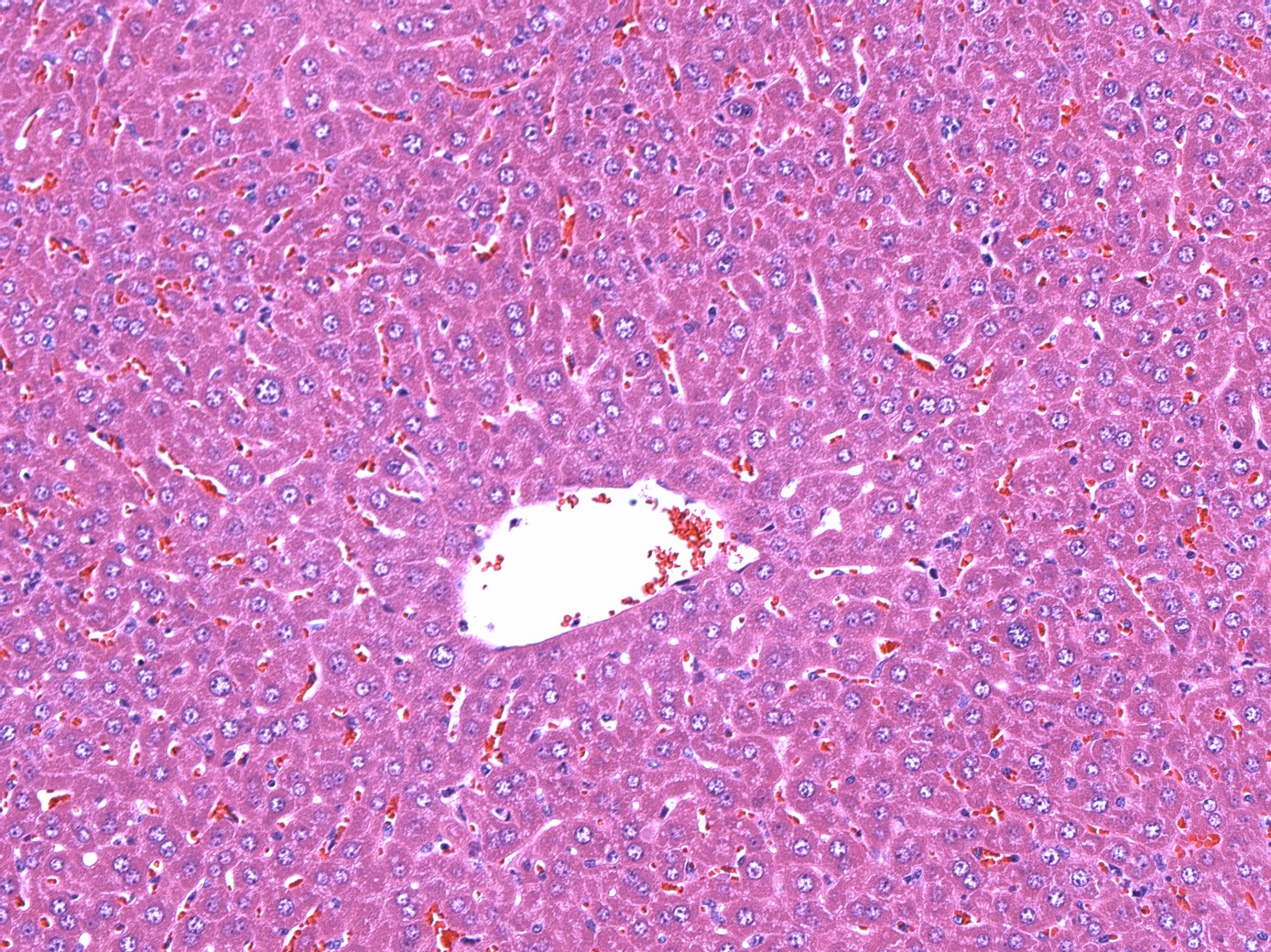

Supplement: Figure 1—source data 4. [file elife-88686-fig1-data4.zip › Liver PBS/Liver_H&E_20x_PBS_7.jpg]

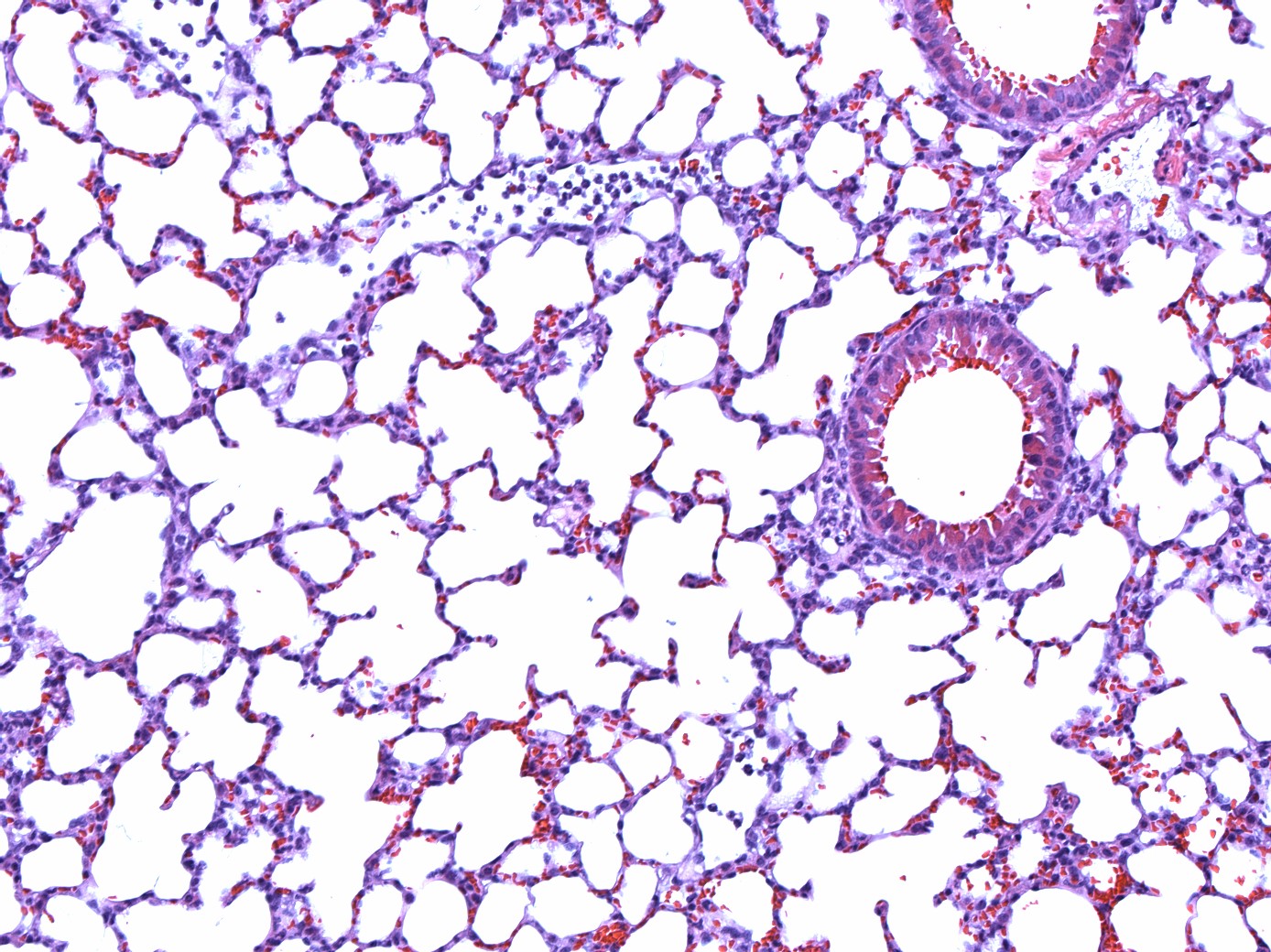

Supplement: Figure 1—source data 4. [file elife-88686-fig1-data4.zip › Lung NAD+/Lung_H&E_20x_NAD_1.jpg]

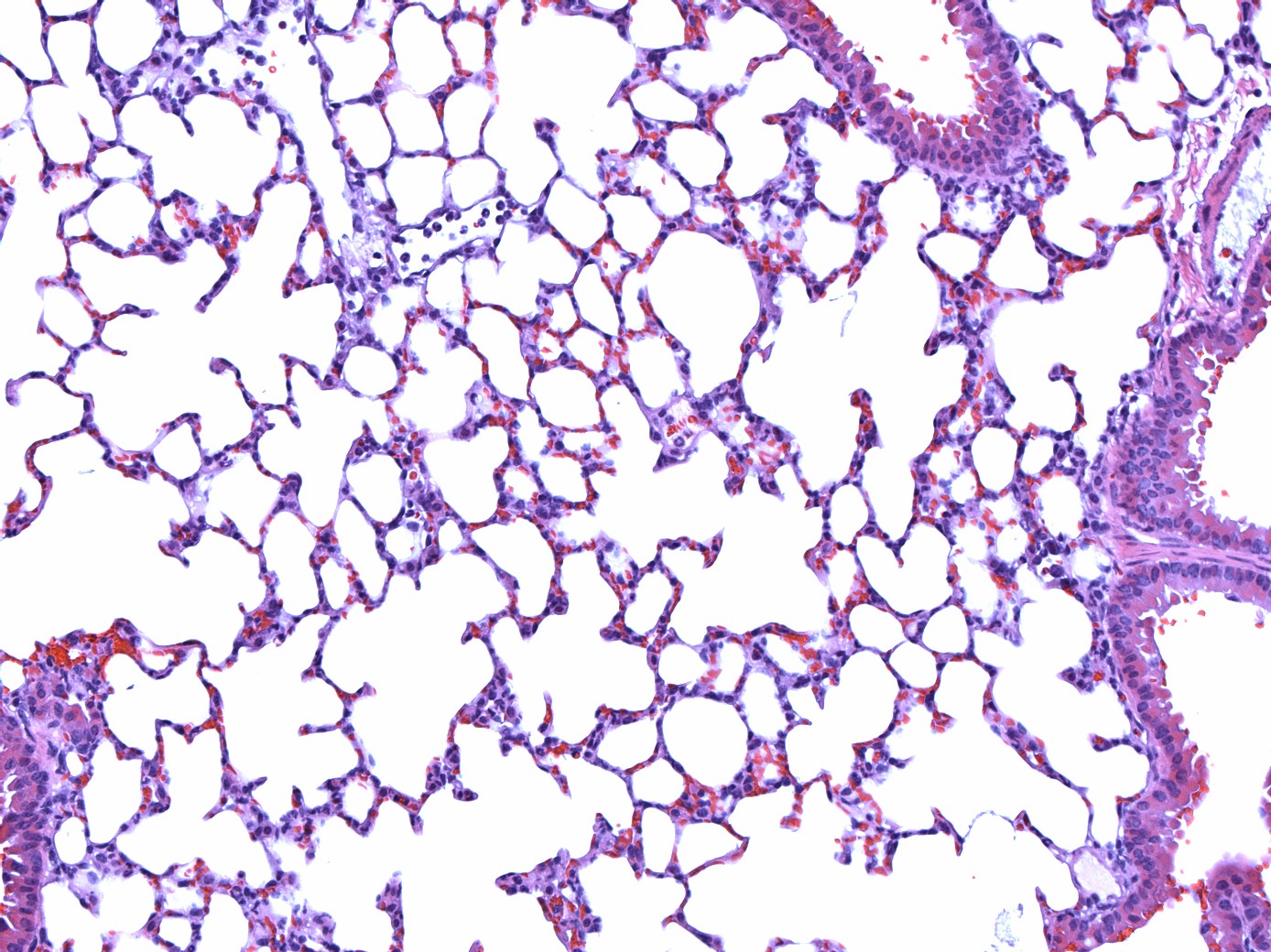

Supplement: Figure 1—source data 4. [file elife-88686-fig1-data4.zip › Lung NAD+/Lung_H&E_20x_NAD_2.jpg]

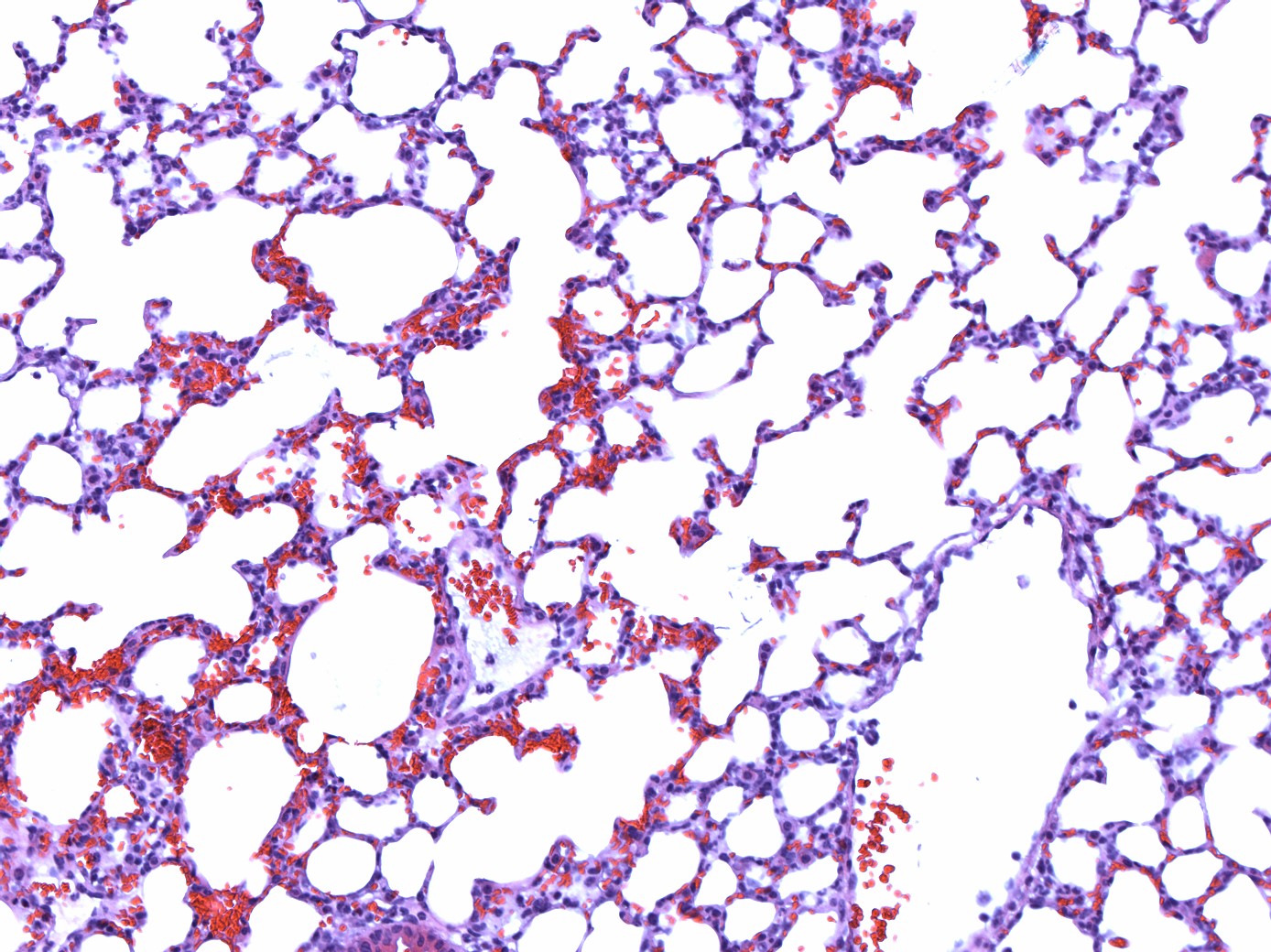

Supplement: Figure 1—source data 4. [file elife-88686-fig1-data4.zip › Lung NAD+/Lung_H&E_20x_NAD_3.jpg]

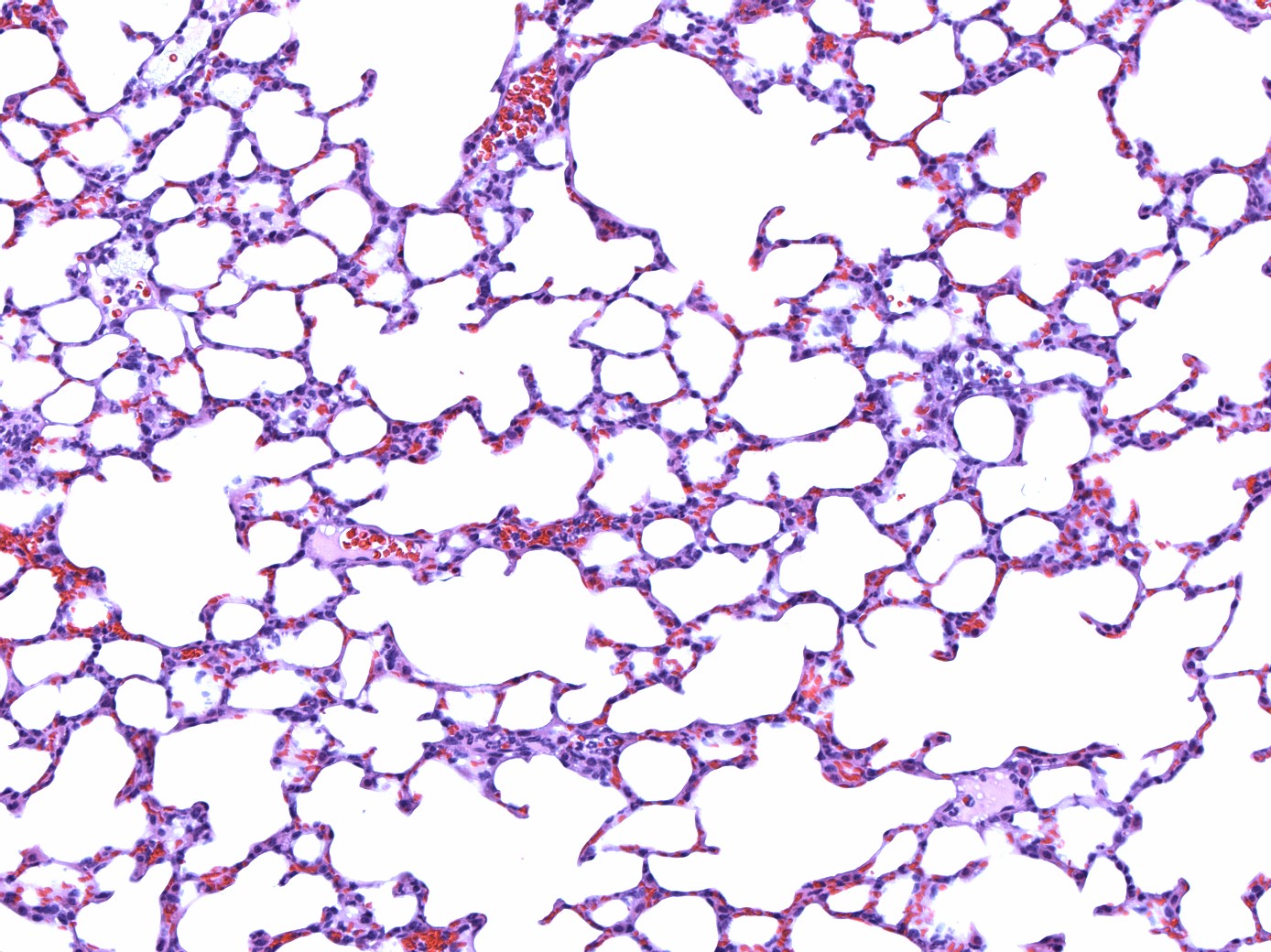

Supplement: Figure 1—source data 4. [file elife-88686-fig1-data4.zip › Lung NAD+/Lung_H&E_20x_NAD_4.jpg]

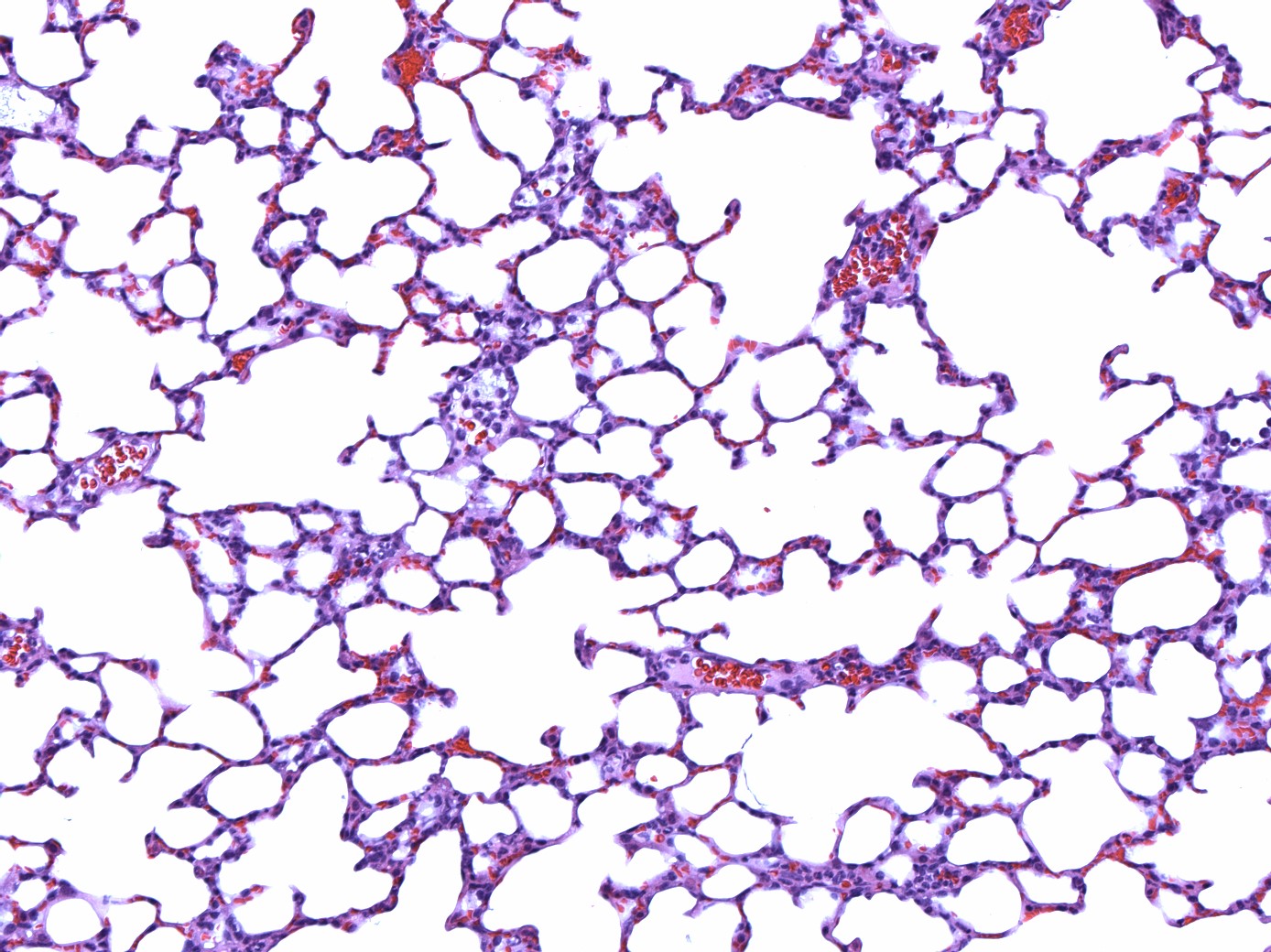

Supplement: Figure 1—source data 4. [file elife-88686-fig1-data4.zip › Lung NAD+/Lung_H&E_20x_NAD_5.jpg]

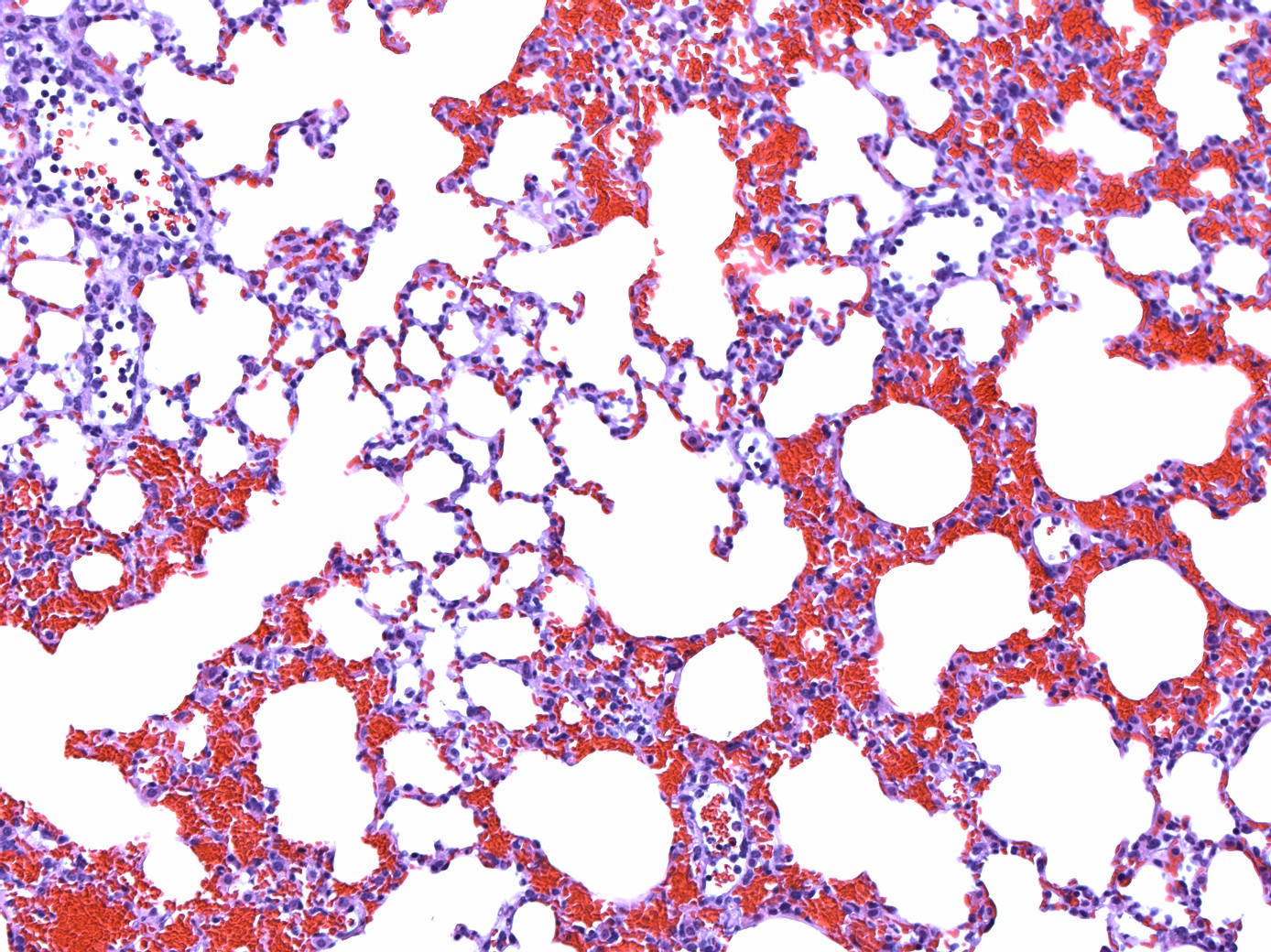

Supplement: Figure 1—source data 4. [file elife-88686-fig1-data4.zip › Lung PBS/Lung_H&E_20x_PBS_1.jpg]

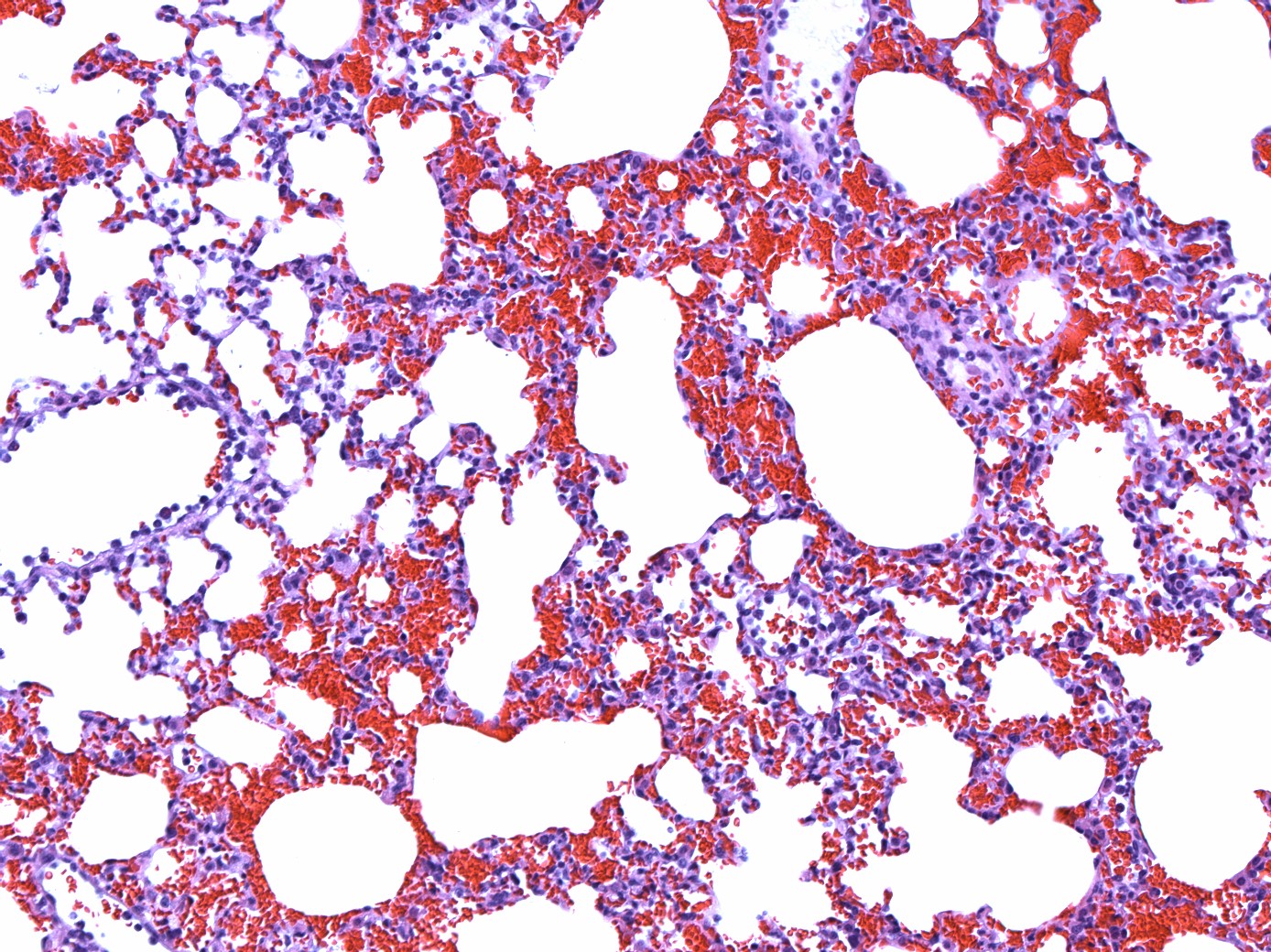

Supplement: Figure 1—source data 4. [file elife-88686-fig1-data4.zip › Lung PBS/Lung_H&E_20x_PBS_2.jpg]

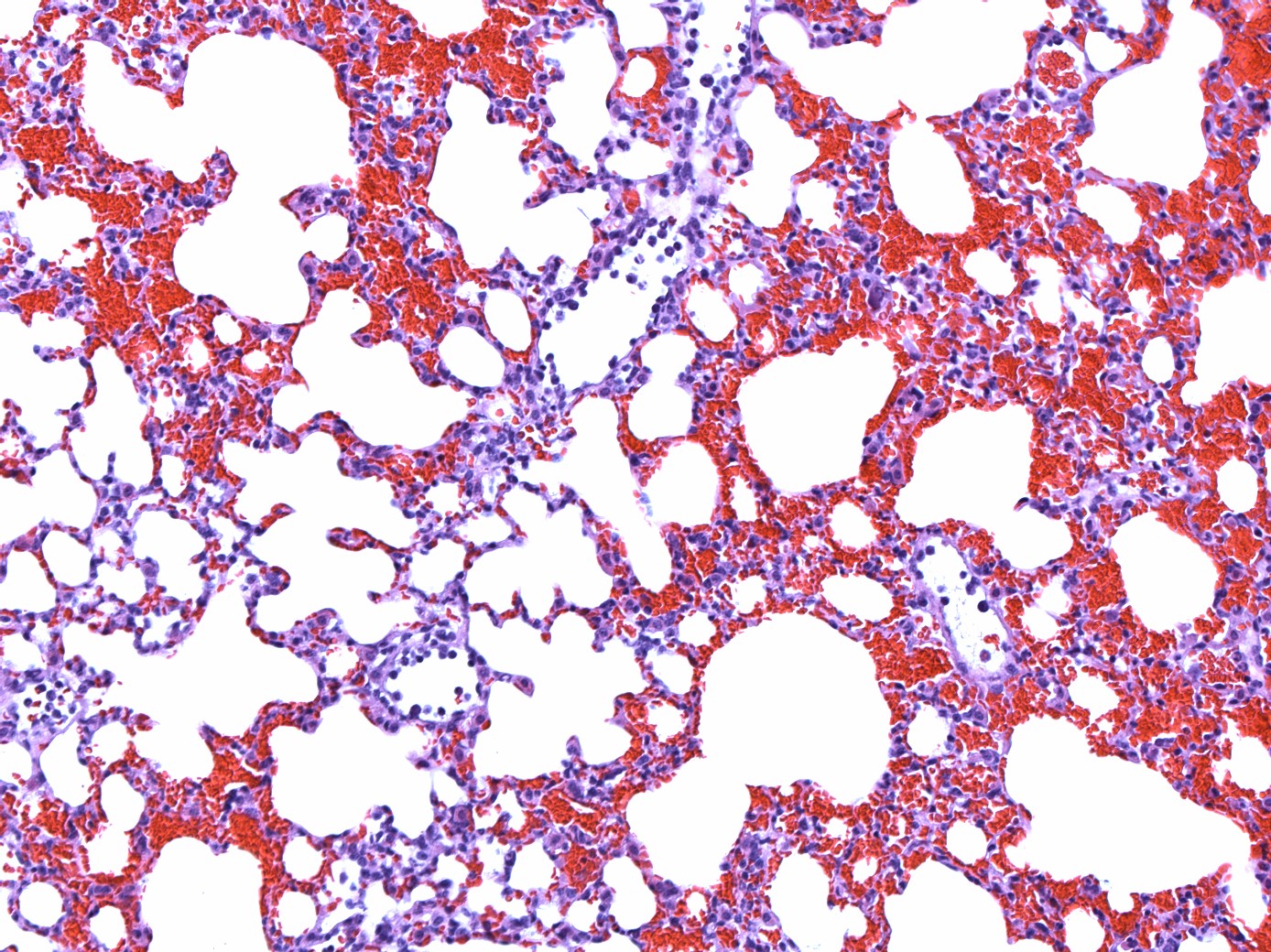

Supplement: Figure 1—source data 4. [file elife-88686-fig1-data4.zip › Lung PBS/Lung_H&E_20x_PBS_3.jpg]

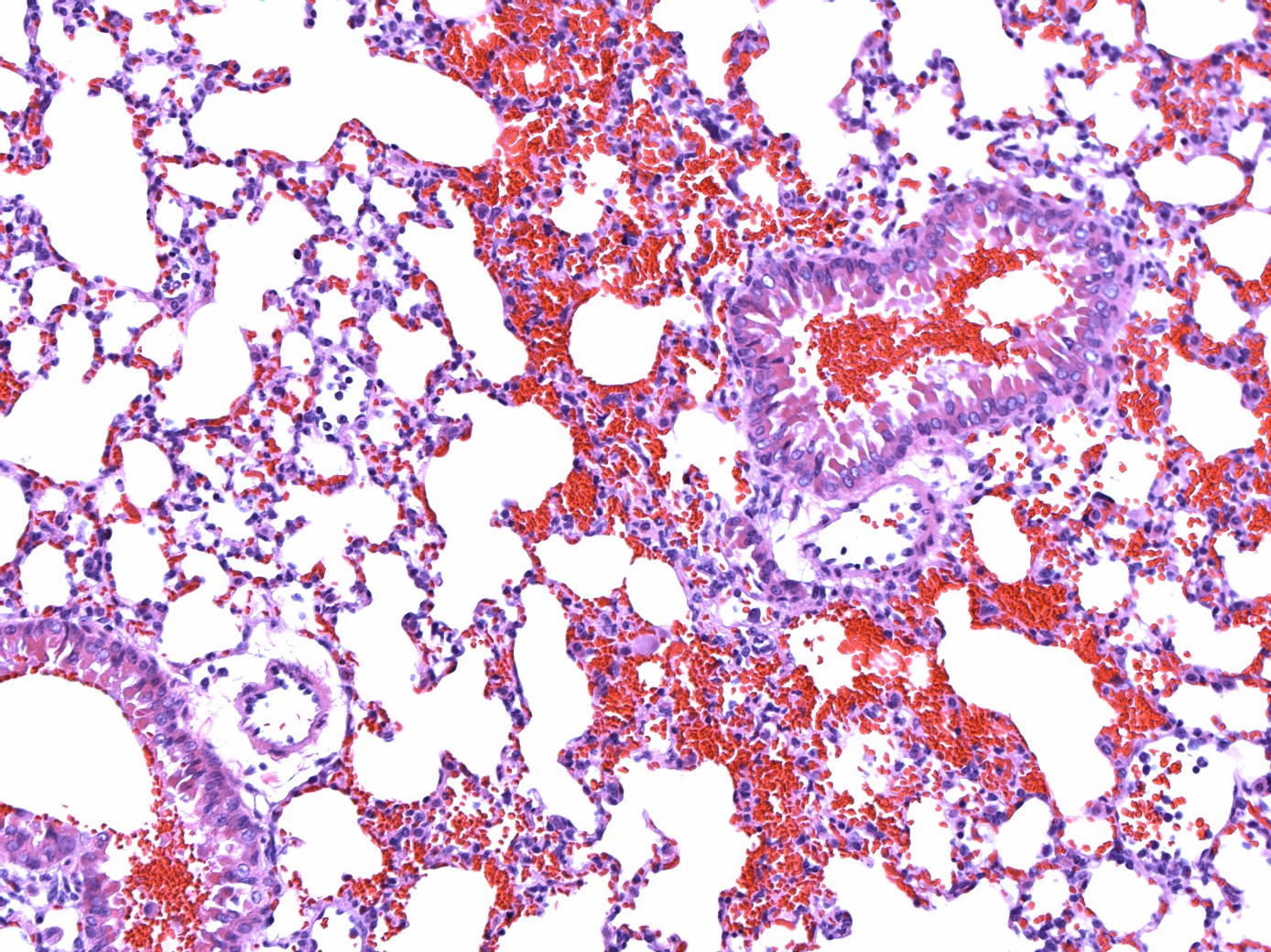

Supplement: Figure 1—source data 4. [file elife-88686-fig1-data4.zip › Lung PBS/Lung_H&E_20x_PBS_4.jpg]

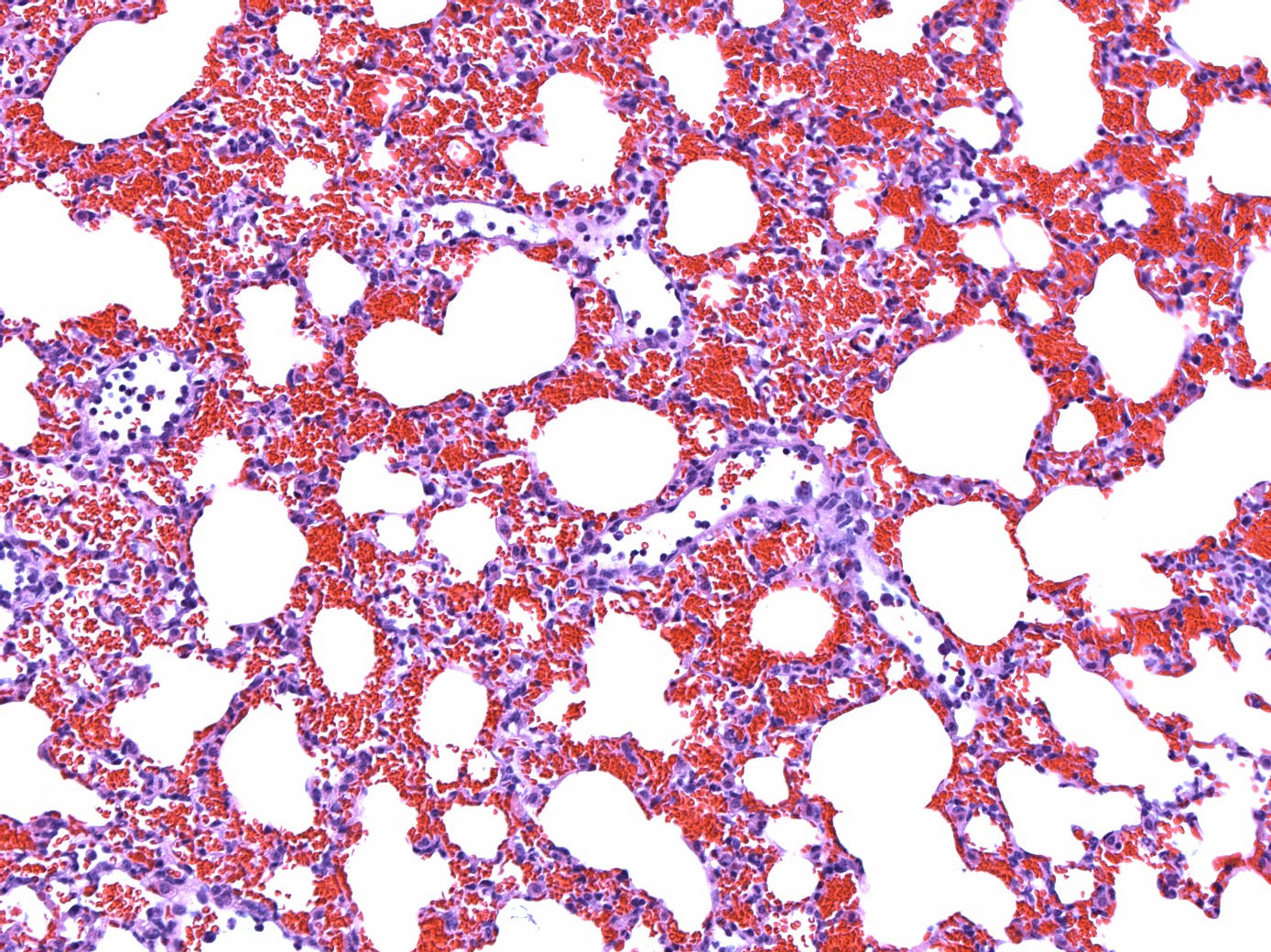

Supplement: Figure 1—source data 4. [file elife-88686-fig1-data4.zip › Lung PBS/Lung_H&E_20x_PBS_5.jpg]

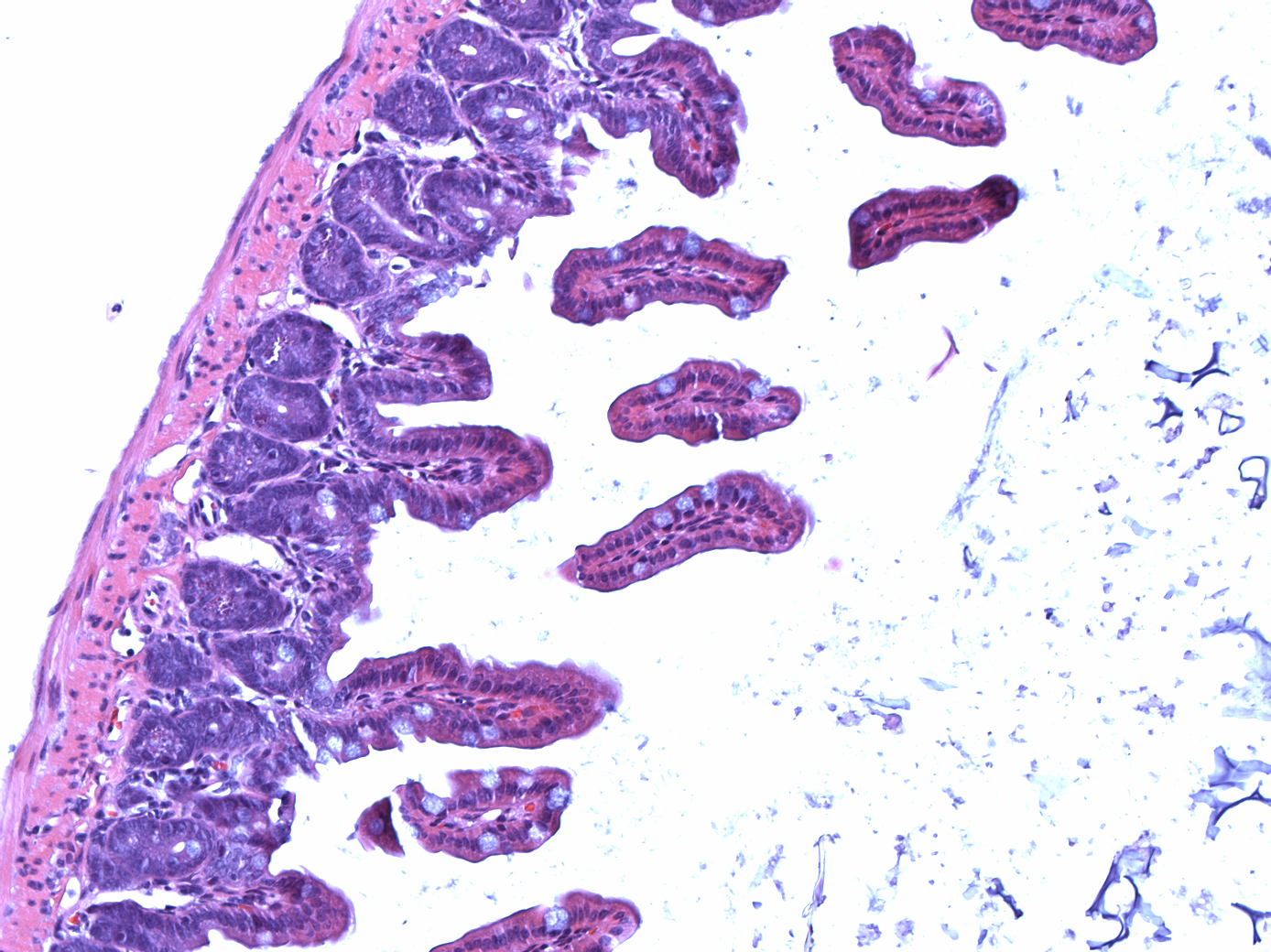

Supplement: Figure 1—figure supplement 1—source data 1. [file elife-88686-fig1-figsupp1-data1.zip › Ileum NAD+/Ileum_H&E_20x_NAD_1_JI_(c1).TIF]

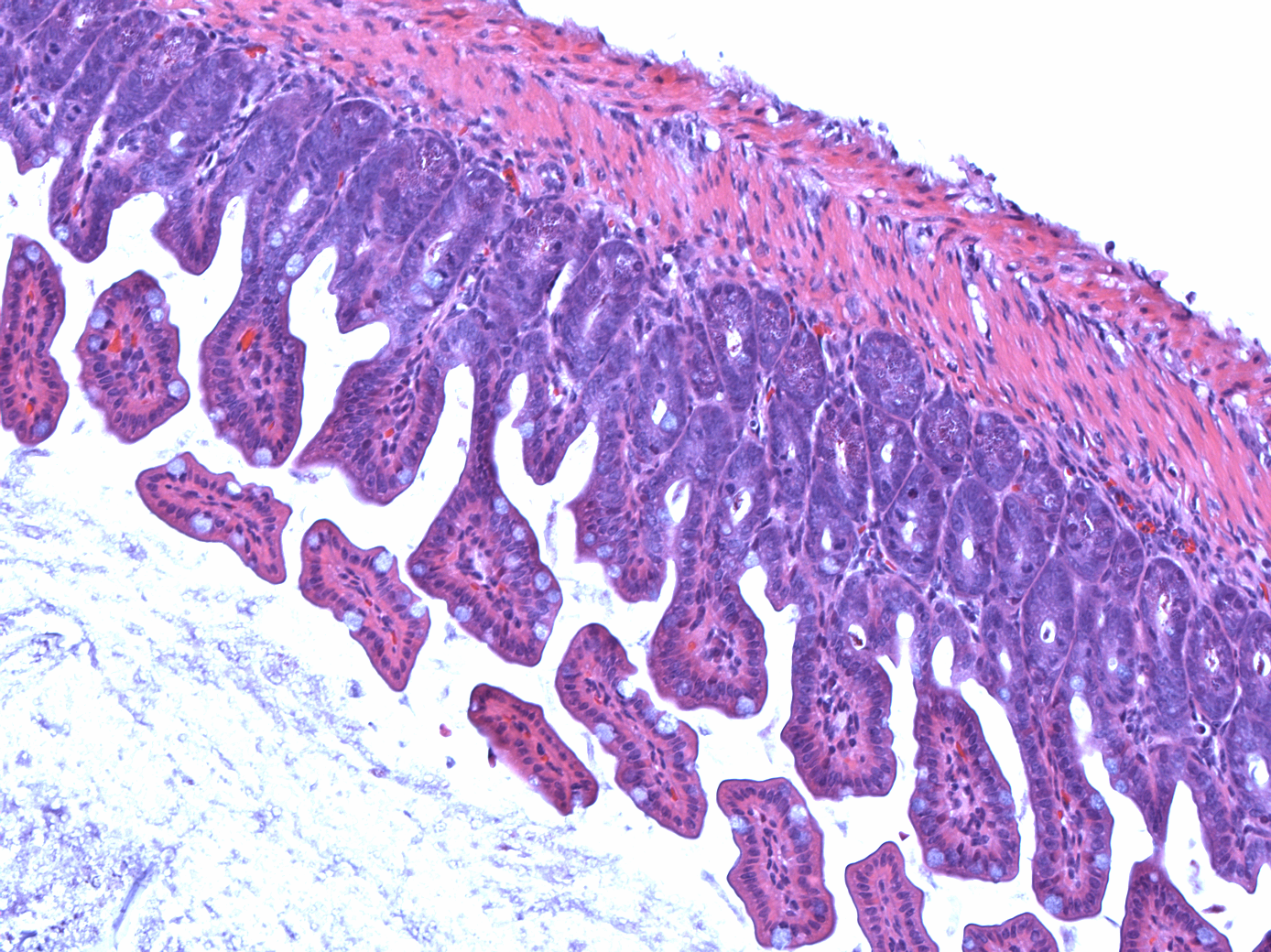

Supplement: Figure 1—figure supplement 1—source data 1. [file elife-88686-fig1-figsupp1-data1.zip › Ileum NAD+/Ileum_H&E_20x_NAD_2_JI_(c1).TIF]

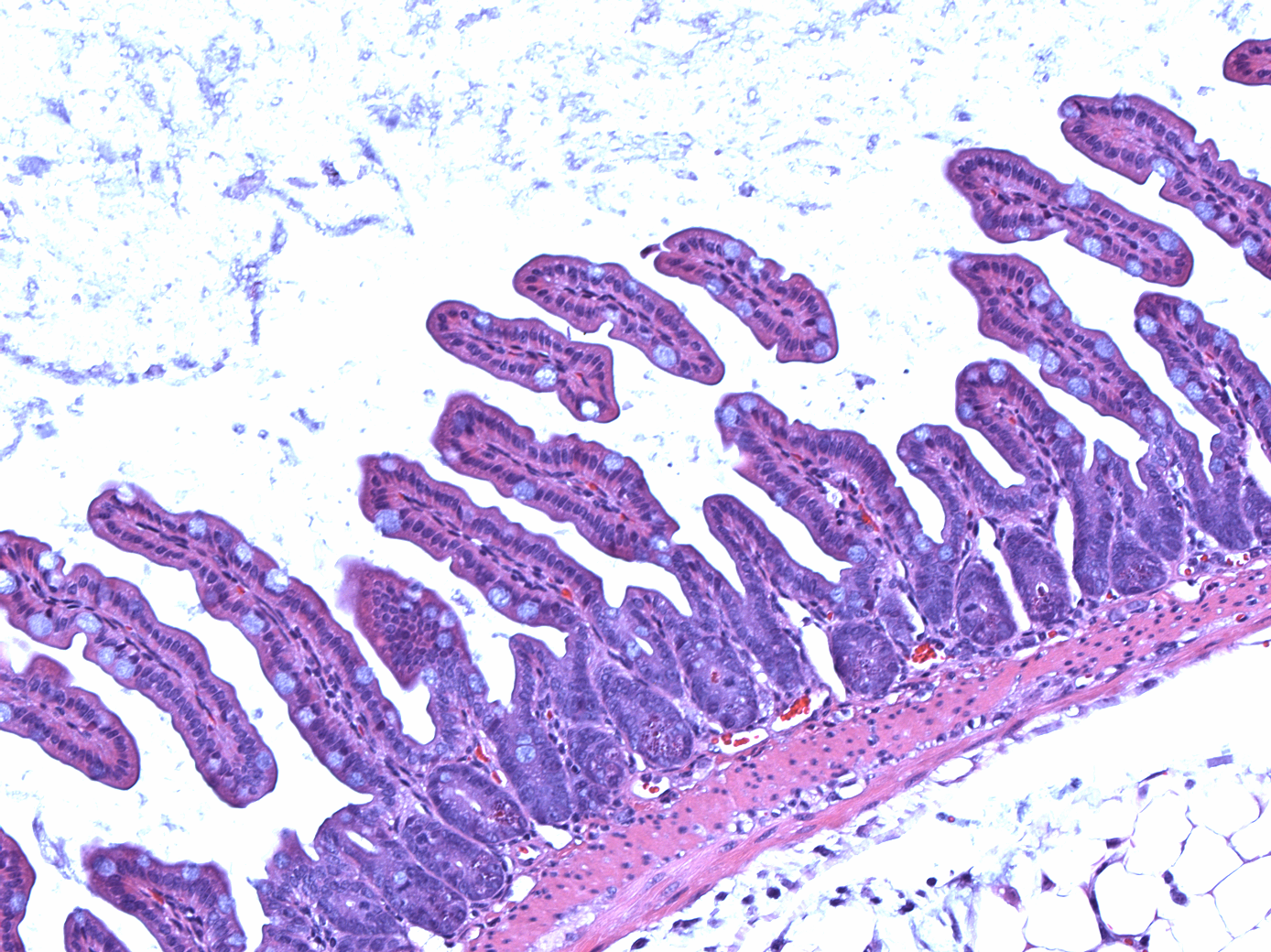

Supplement: Figure 1—figure supplement 1—source data 1. [file elife-88686-fig1-figsupp1-data1.zip › Ileum NAD+/Ileum_H&E_20x_NAD_3_JI_(c1).TIF]

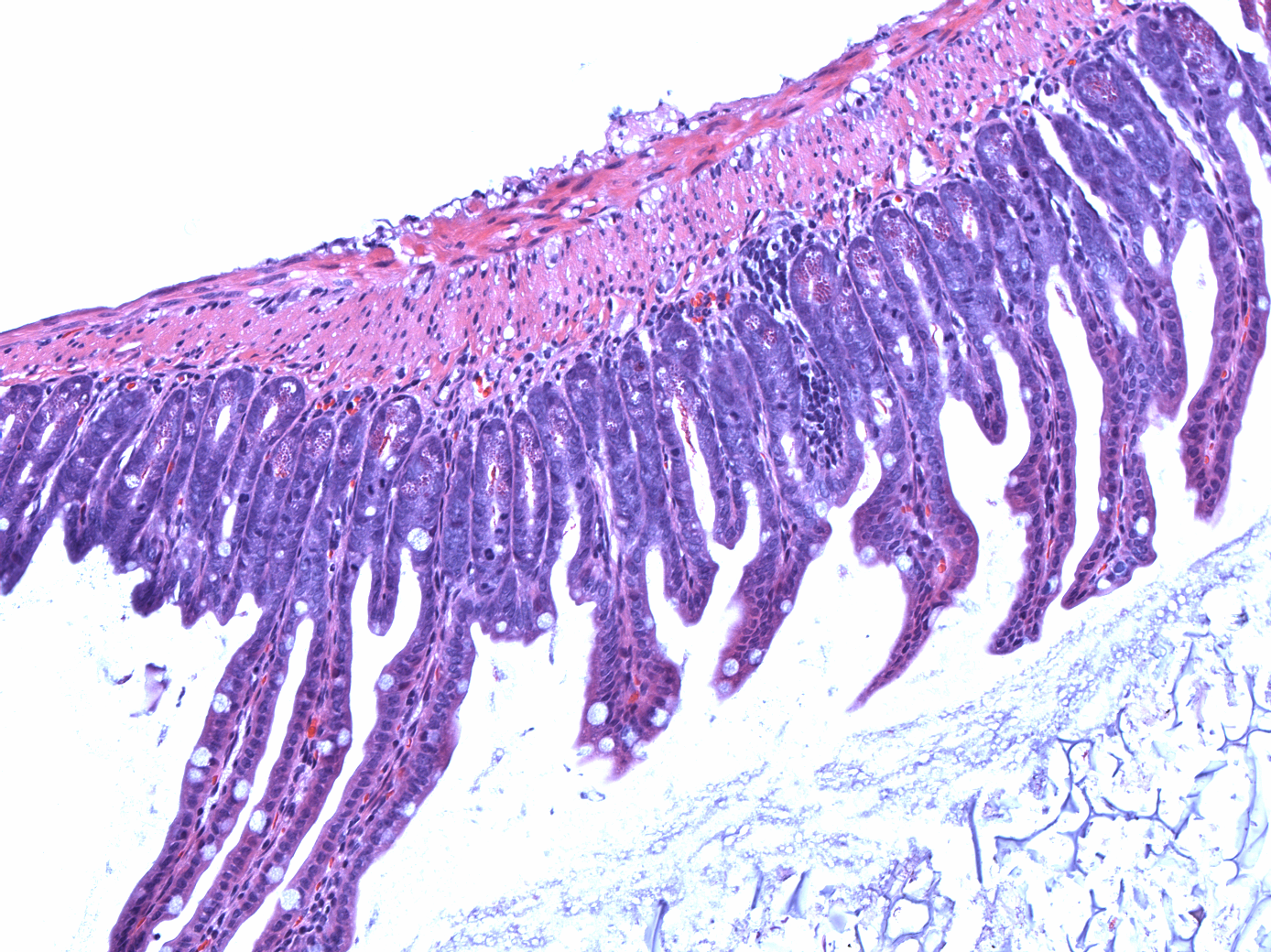

Supplement: Figure 1—figure supplement 1—source data 1. [file elife-88686-fig1-figsupp1-data1.zip › Ileum NAD+/Ileum_H&E_20x_NAD_4_JI_(c1).TIF]

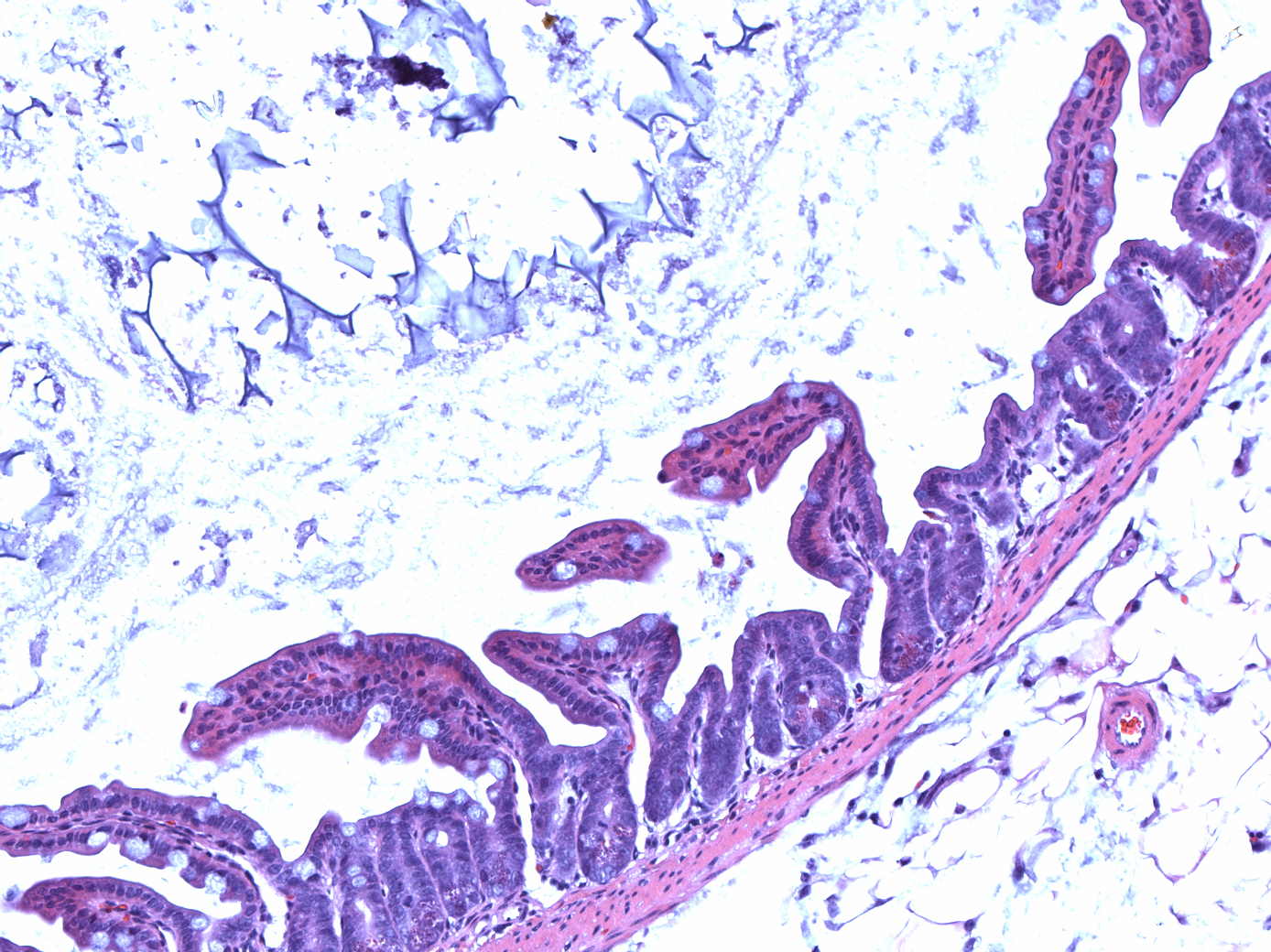

Supplement: Figure 1—figure supplement 1—source data 1. [file elife-88686-fig1-figsupp1-data1.zip › Ileum NAD+/Ileum_H&E_20x_NAD_5_JI_(c1).TIF]

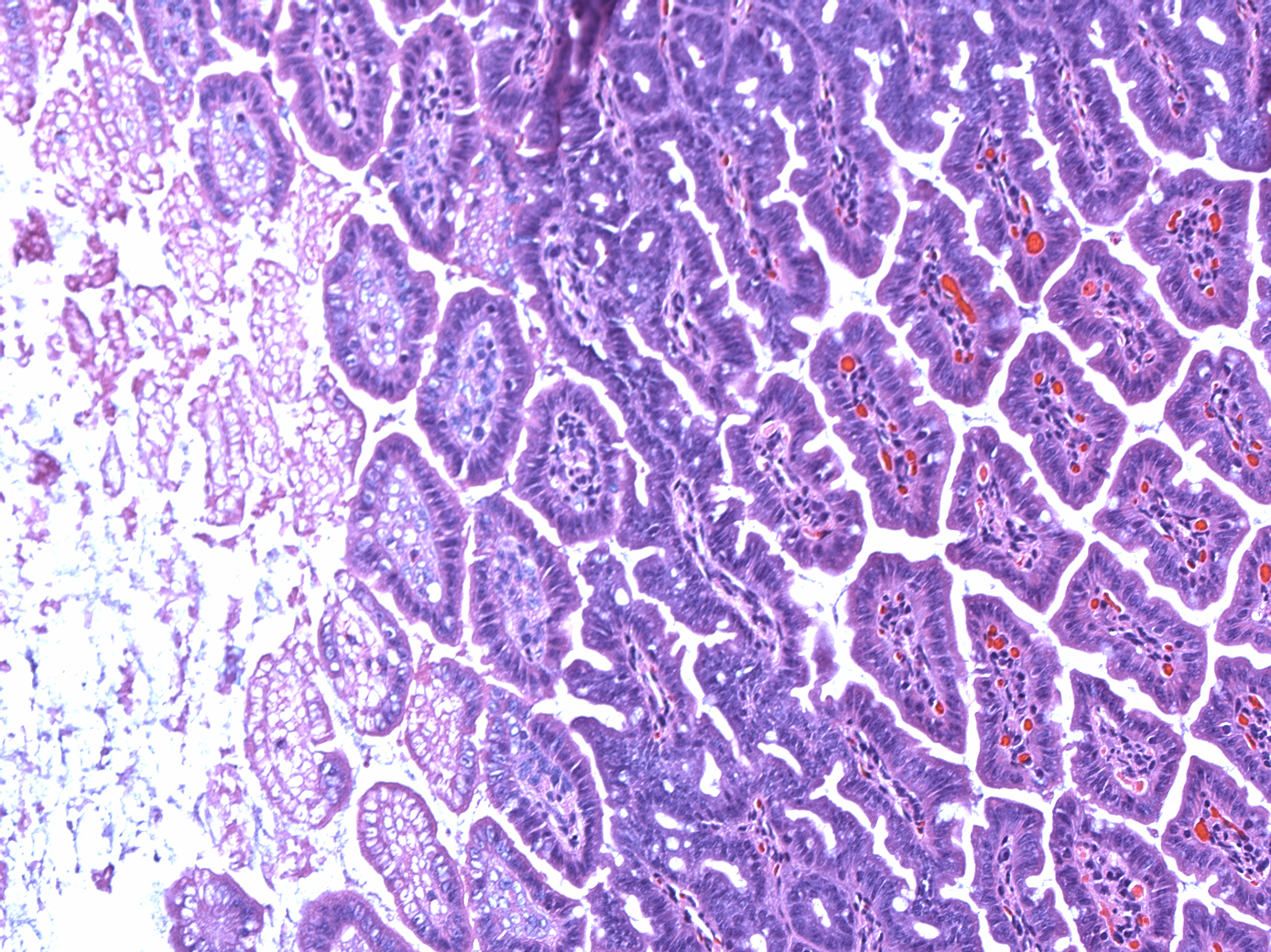

Supplement: Figure 1—figure supplement 1—source data 1. [file elife-88686-fig1-figsupp1-data1.zip › Ileum PBS/Ileum_H&E_20x_LPS_1_JI_(c1).TIF]

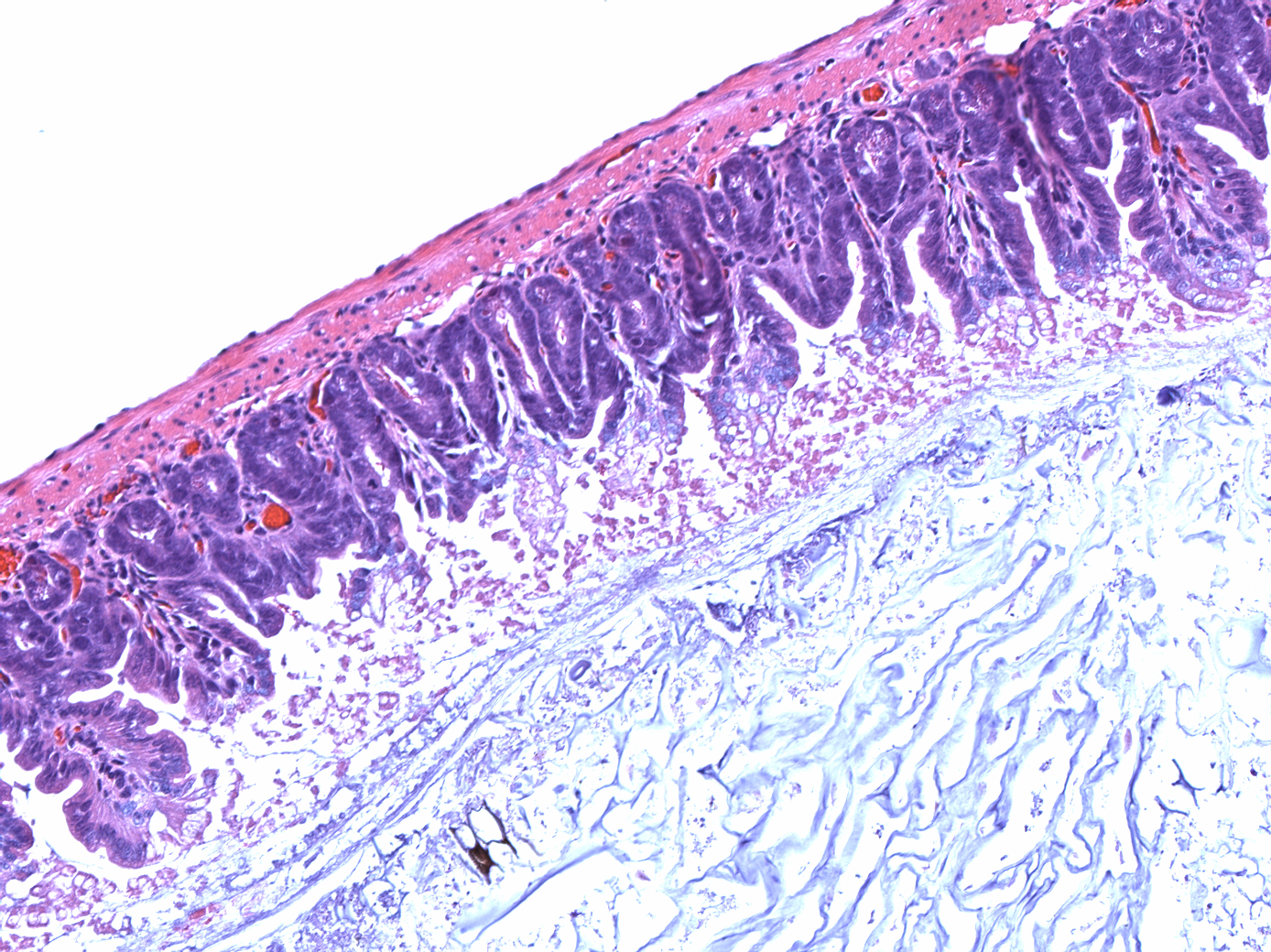

Supplement: Figure 1—figure supplement 1—source data 1. [file elife-88686-fig1-figsupp1-data1.zip › Ileum PBS/Ileum_H&E_20x_LPS_2_JI_(c1).TIF]

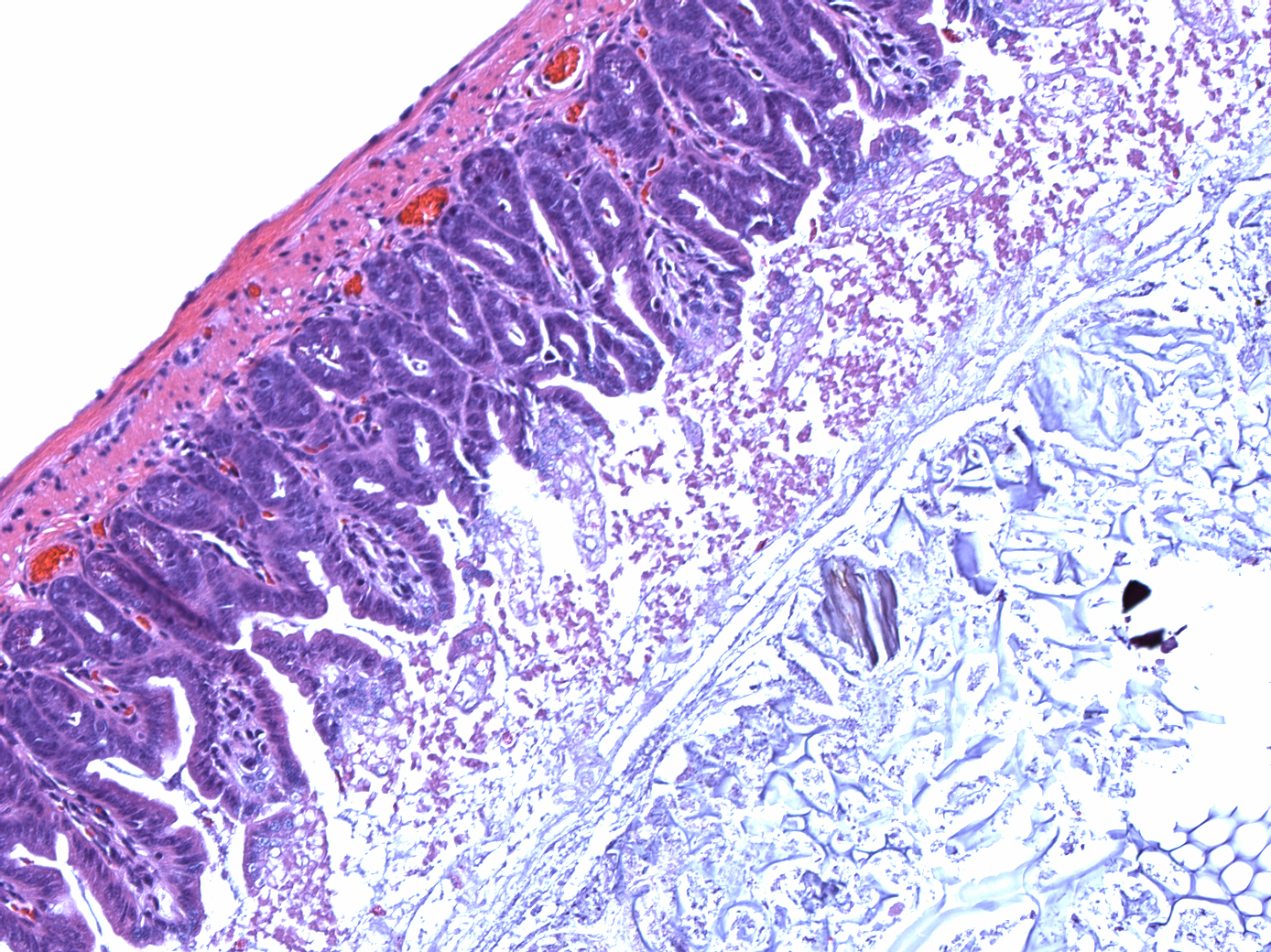

Supplement: Figure 1—figure supplement 1—source data 1. [file elife-88686-fig1-figsupp1-data1.zip › Ileum PBS/Ileum_H&E_20x_PBS_3_JI_(c1).TIF]

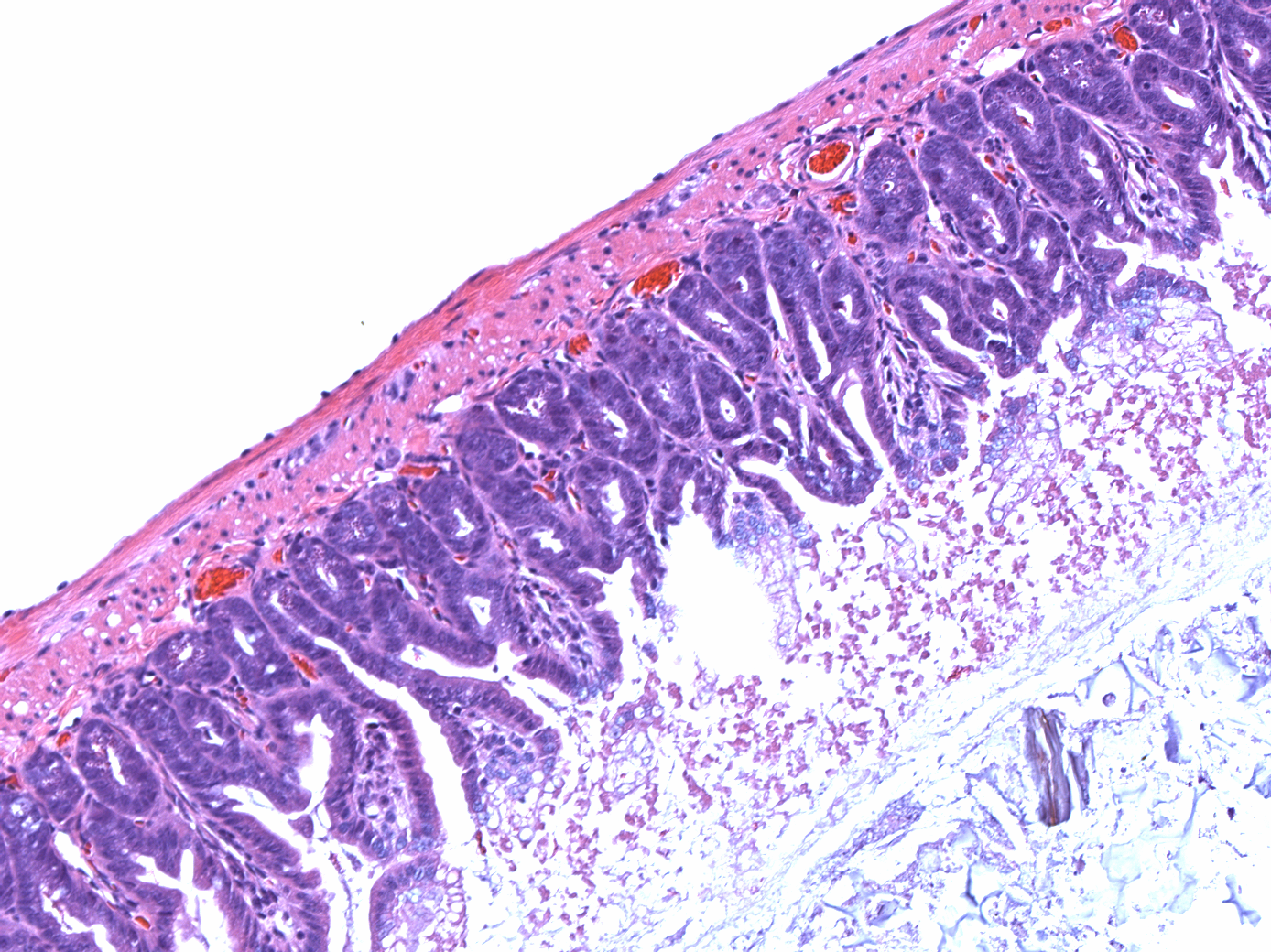

Supplement: Figure 1—figure supplement 1—source data 1. [file elife-88686-fig1-figsupp1-data1.zip › Ileum PBS/Ileum_H&E_20x_PBS_4_JI_(c1).TIF]

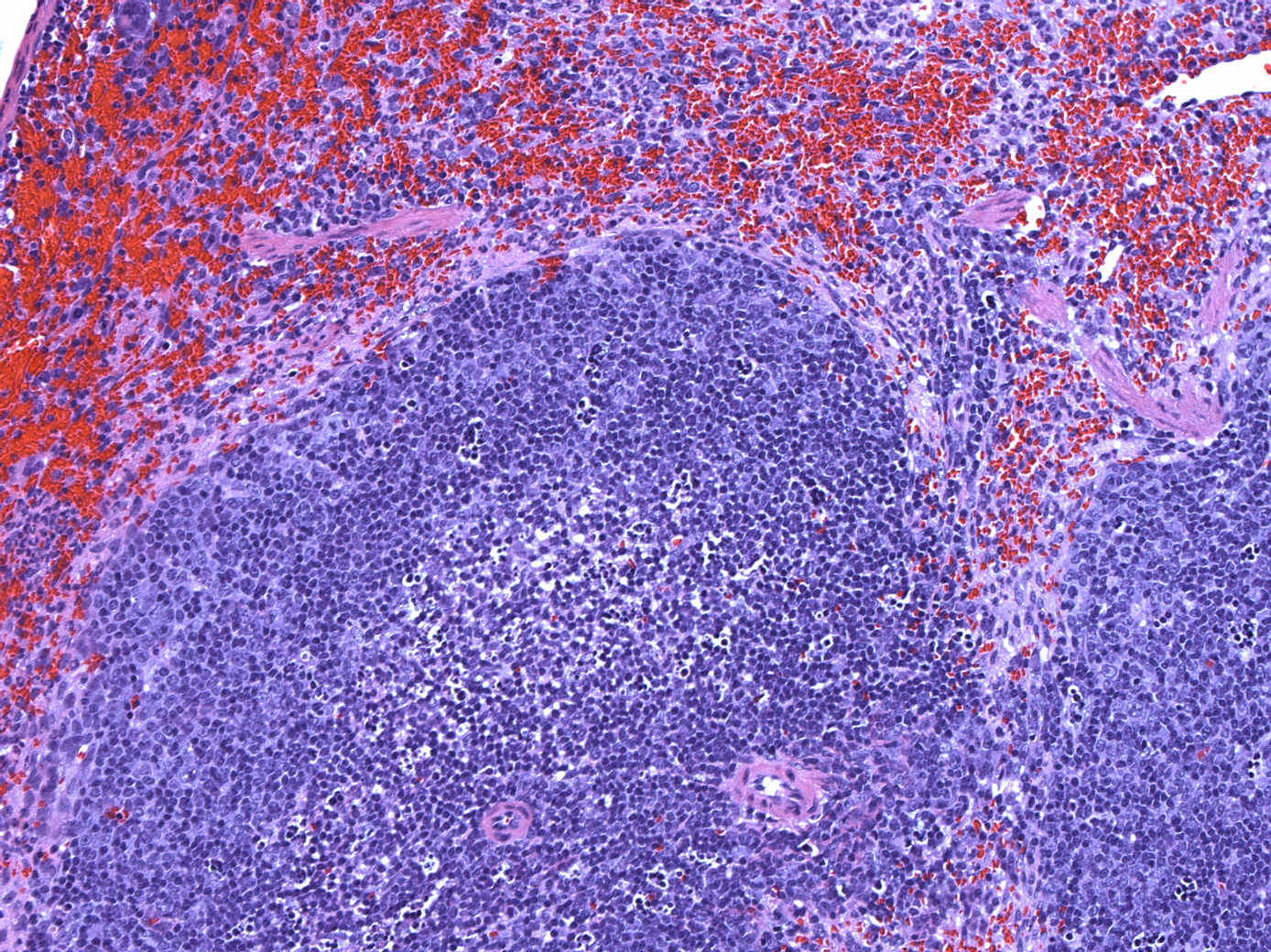

Supplement: Figure 1—figure supplement 1—source data 1. [file elife-88686-fig1-figsupp1-data1.zip › Spleen NAD+/Spleen_H&E_20x_NAD_1_JI_(c1).TIF]

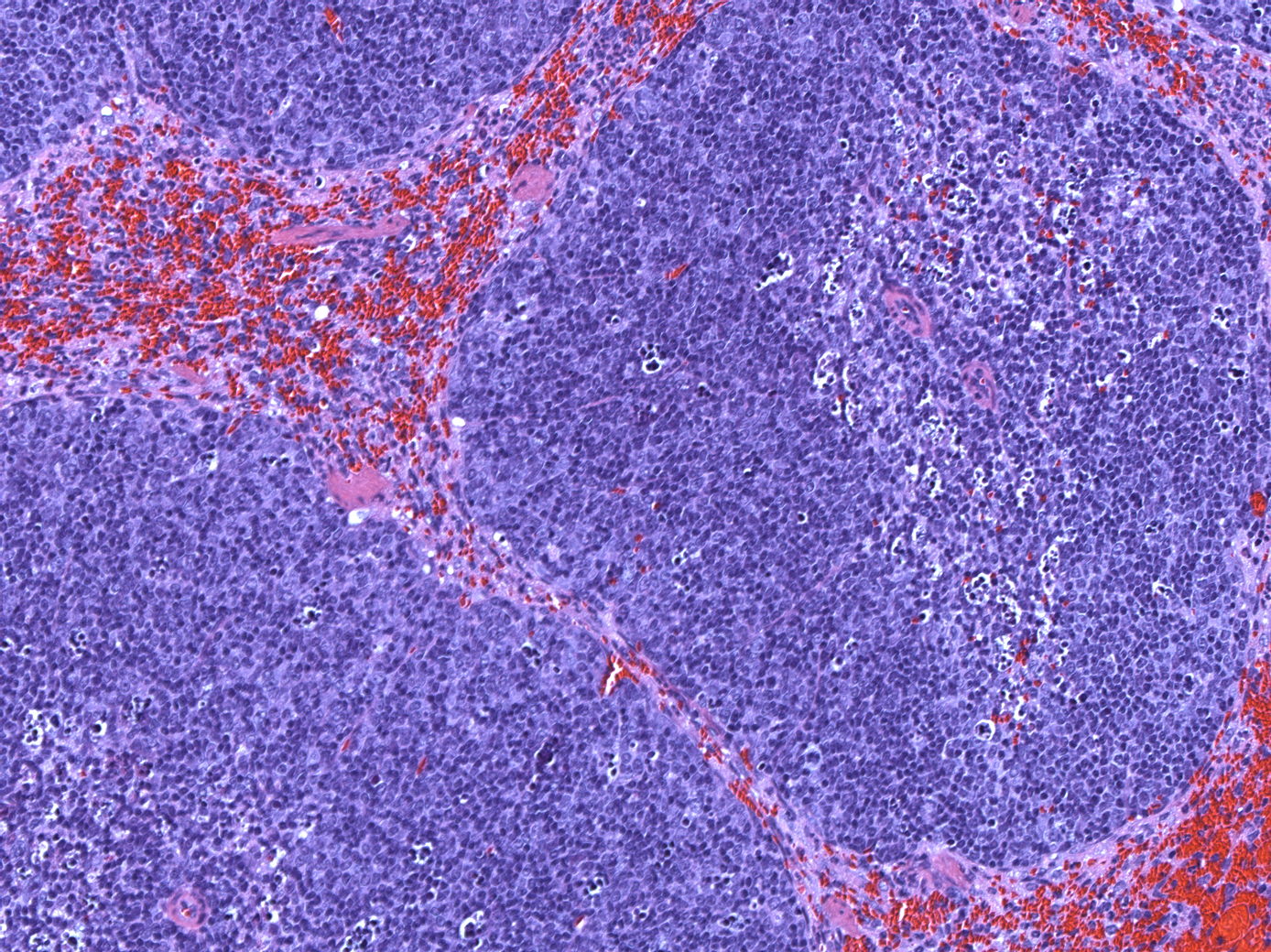

Supplement: Figure 1—figure supplement 1—source data 1. [file elife-88686-fig1-figsupp1-data1.zip › Spleen NAD+/Spleen_H&E_20x_NAD_2_JI_(c1).TIF]

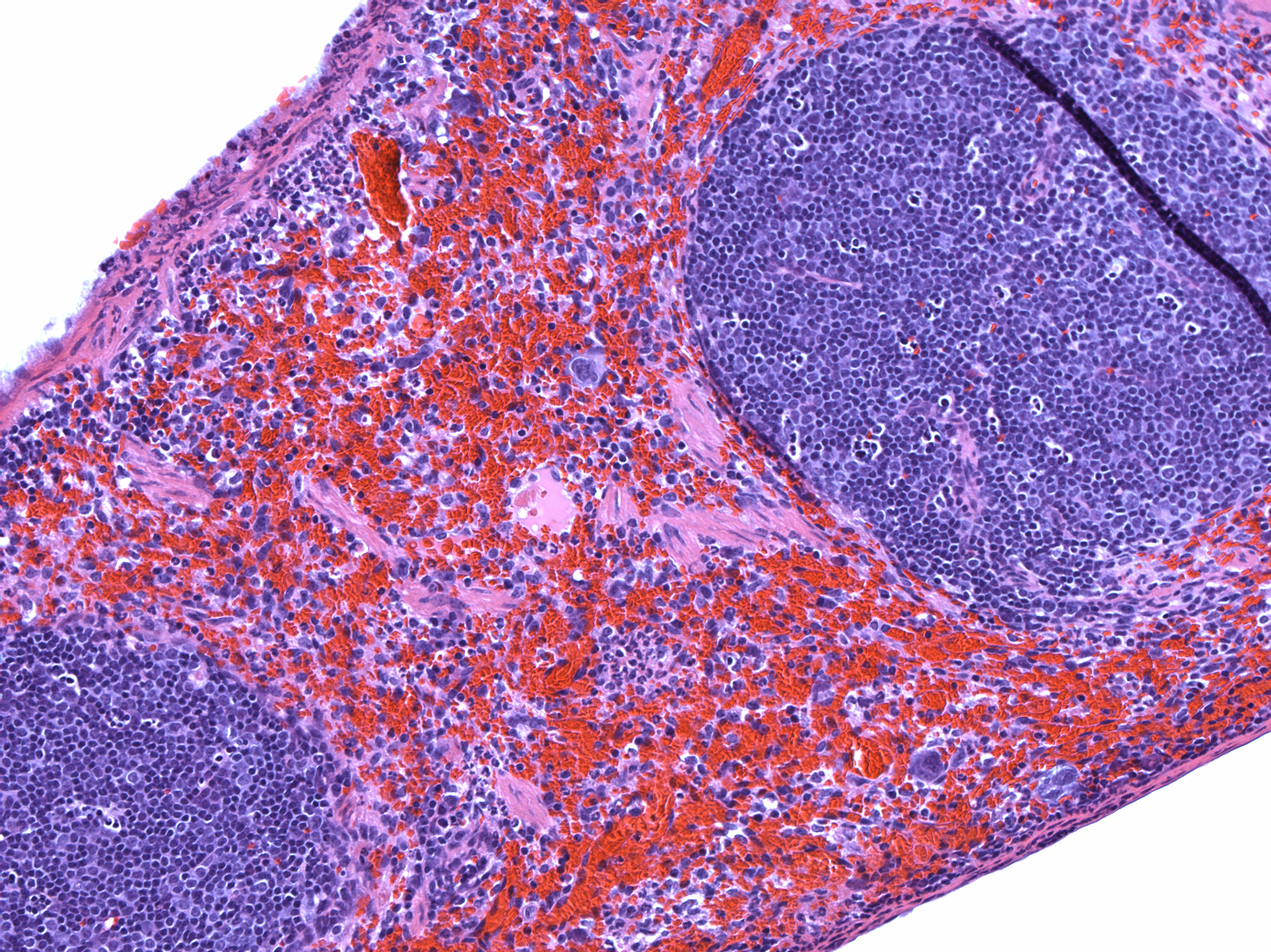

Supplement: Figure 1—figure supplement 1—source data 1. [file elife-88686-fig1-figsupp1-data1.zip › Spleen NAD+/Spleen_H&E_20x_NAD_3_JI_(c1).TIF]

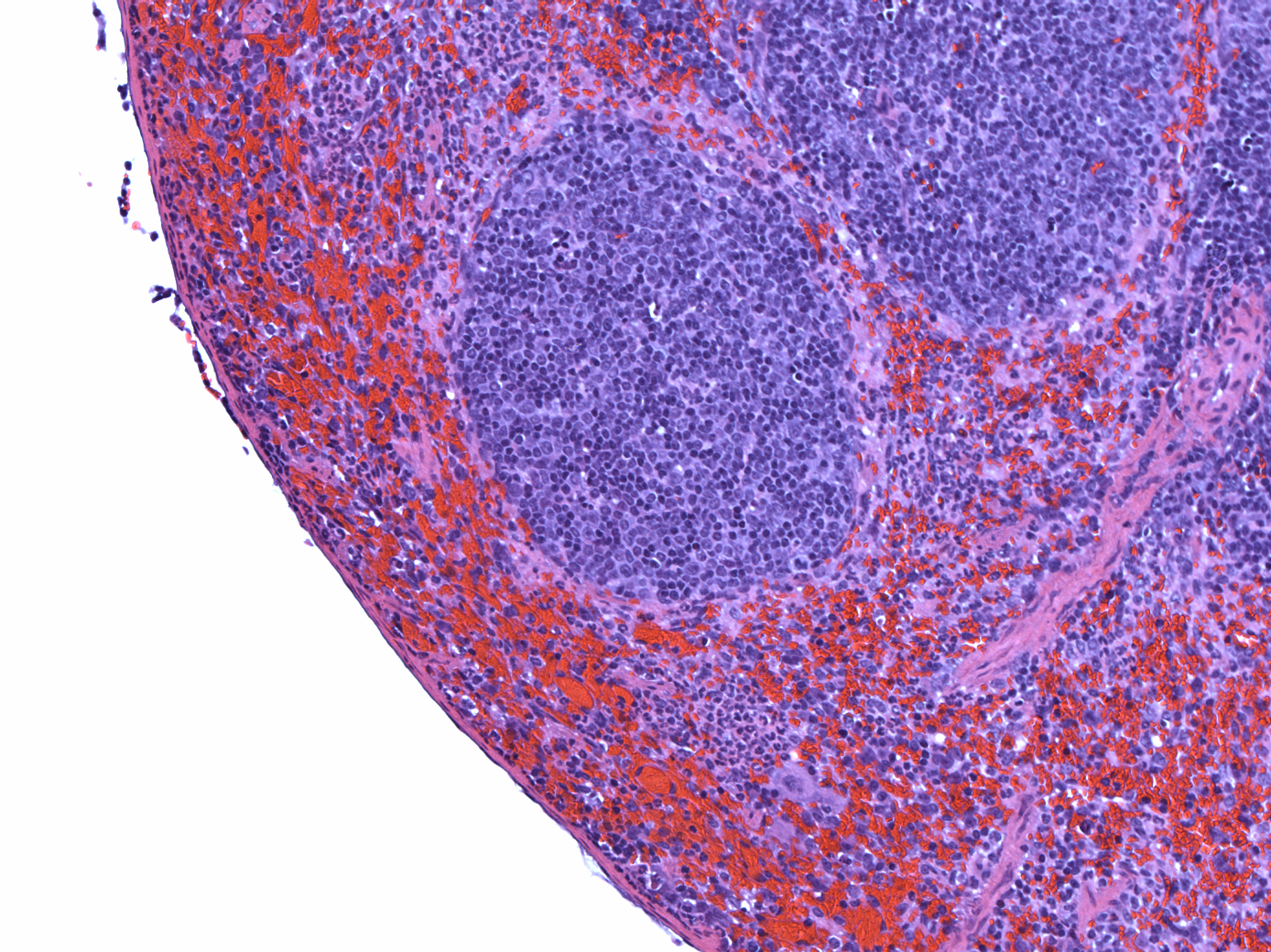

Supplement: Figure 1—figure supplement 1—source data 1. [file elife-88686-fig1-figsupp1-data1.zip › Spleen NAD+/Spleen_H&E_20x_NAD_4_JI_(c1).TIF]

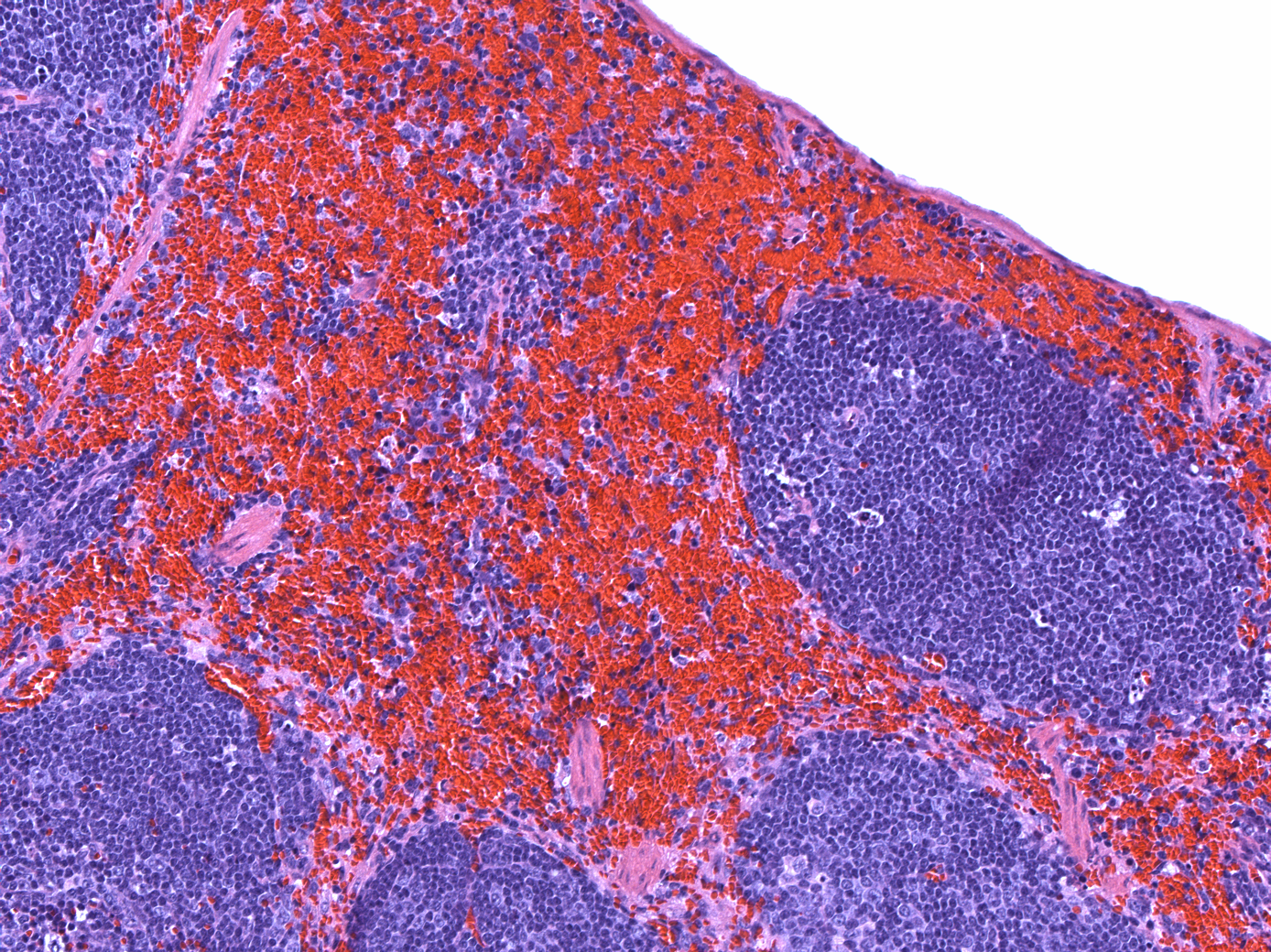

Supplement: Figure 1—figure supplement 1—source data 1. [file elife-88686-fig1-figsupp1-data1.zip › Spleen PBS/Spleen_H&E_20x_PBS_1_JI_(c1).TIF]

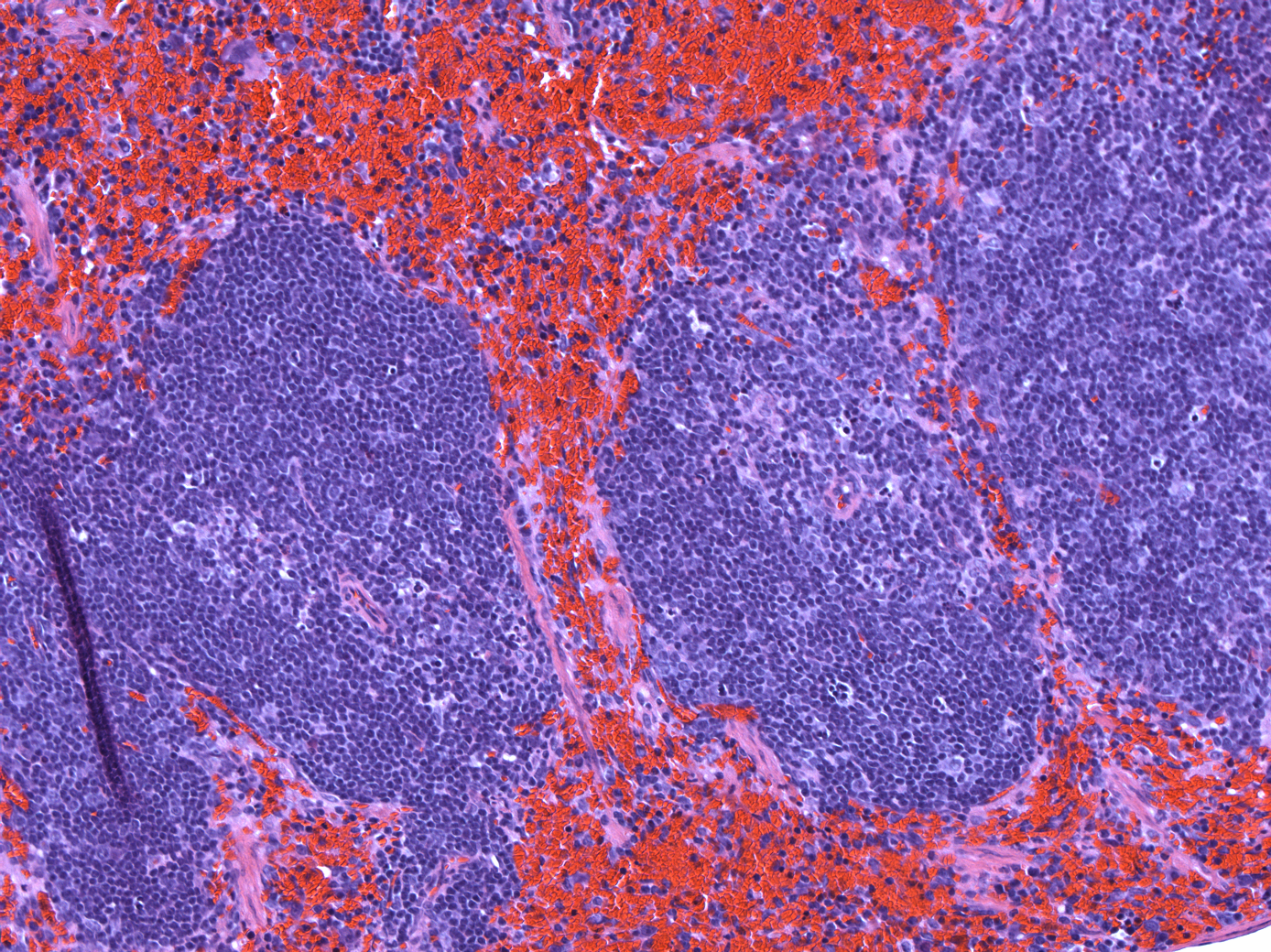

Supplement: Figure 1—figure supplement 1—source data 1. [file elife-88686-fig1-figsupp1-data1.zip › Spleen PBS/Spleen_H&E_20x_PBS_2_JI_(c1).TIF]

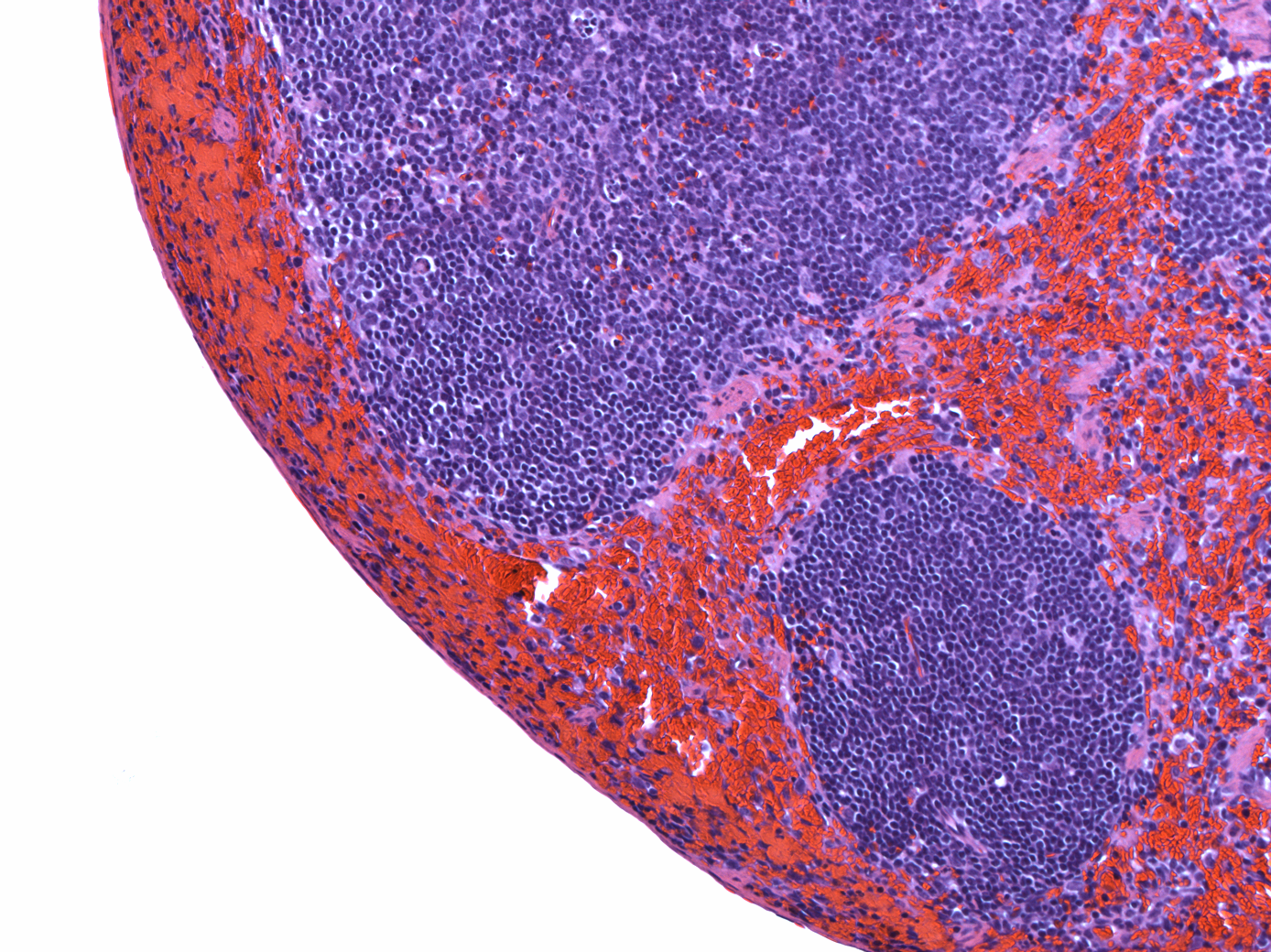

Supplement: Figure 1—figure supplement 1—source data 1. [file elife-88686-fig1-figsupp1-data1.zip › Spleen PBS/Spleen_H&E_20x_PBS_3_JI_(c1).TIF]

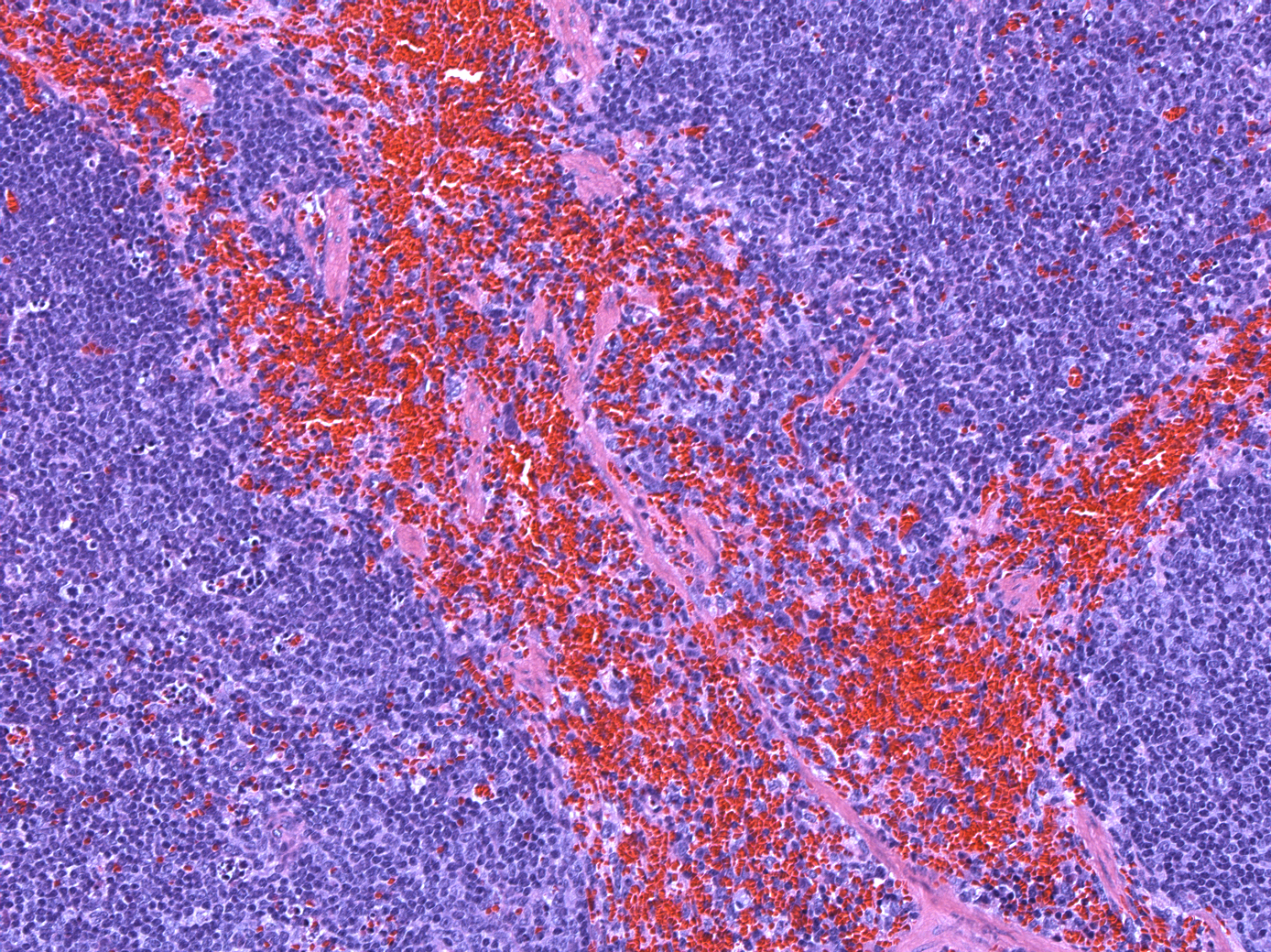

Supplement: Figure 1—figure supplement 1—source data 1. [file elife-88686-fig1-figsupp1-data1.zip › Spleen PBS/Spleen_H&E_20x_PBS_4_JI_(c1).TIF]

Actin

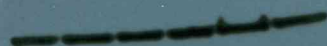

L

- . 250
- . 130
- . 95
- \* 72
- . 55
- . 36
- \* 28
- ┘

Supplement: Figure 2—source data 1. [file elife-88686-fig2-data1.zip › Figure 2A. Canonical Inflammasome Actin.pdf]

KDa <sup>page</sup>  
 ruler <sup>miles</sup>  
 72 \*  
 55 -  
 43 -  
 34 -  
 26 -  
 17 -

1 2 3 4 5 6 Novex

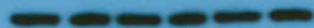

^p

72 \*  
 55 .  
 43 .  
 34 .

1 2 3 4 5 6 Nov

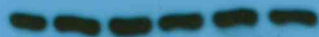

①

Supplement: Figure 2—source data 1. [file elife-88686-fig2-data1.zip › Figure 2A. Canonical Inflammasome Casp 1,IL-1beta.pdf]

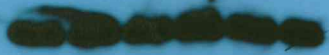

7

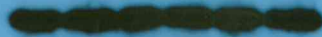

7

GDD

L

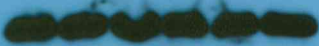

910 ->  
11 ->

7

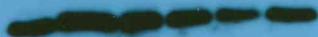

7

- (H-100)  
- (H-100)  
- (H-100)  
- (H-100)

Supplement: Figure 2—source data 1. [file elife-88686-fig2-data1.zip › Figure 2A. Canonical Inflammasome GSDMD, Pro-Casp 11, Casp 11.pdf]

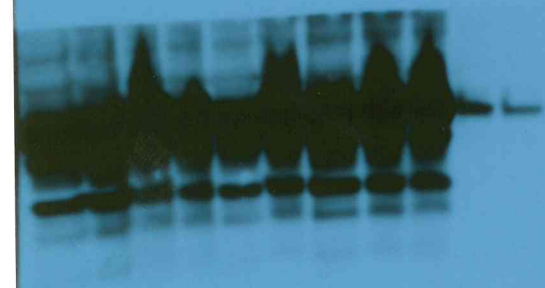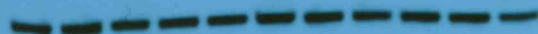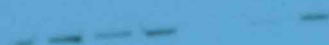

Supplement: Figure 2—source data 1. [file elife-88686-fig2-data1.zip › Figure 2A. Non Canonical Inflammasome Actin.pdf]

9D

$$\frac{N_A - N_S}{N} \quad \frac{C_1 - C_2}{C} \times \times$$

4 3 2 1 4 3 2 1 x x

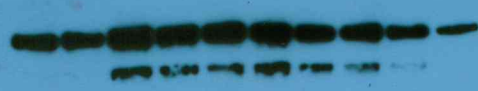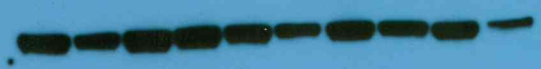

.95  
.82  
.55  
(1)  
.34  
.26  
.17

6SPms

Supplement: Figure 2—source data 1. [file elife-88686-fig2-data1.zip › Figure 2A. Non Canonical Inflammasome GSDMD.pdf]

C1-C4    N1-N5    N1-N5

55 •  
35 •  
25 •  
15 •  
10 •

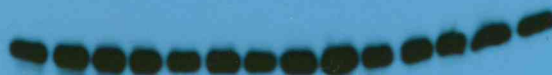

Pro-  
CASPASE  
Ab cam  
anti 3

Pro Casp 1

Supplement: Figure 2—source data 1. [file elife-88686-fig2-data1.zip › Figure 2A. Non Canonical Inflammasome Pro-Casp 1.pdf]

# Actin

Actin

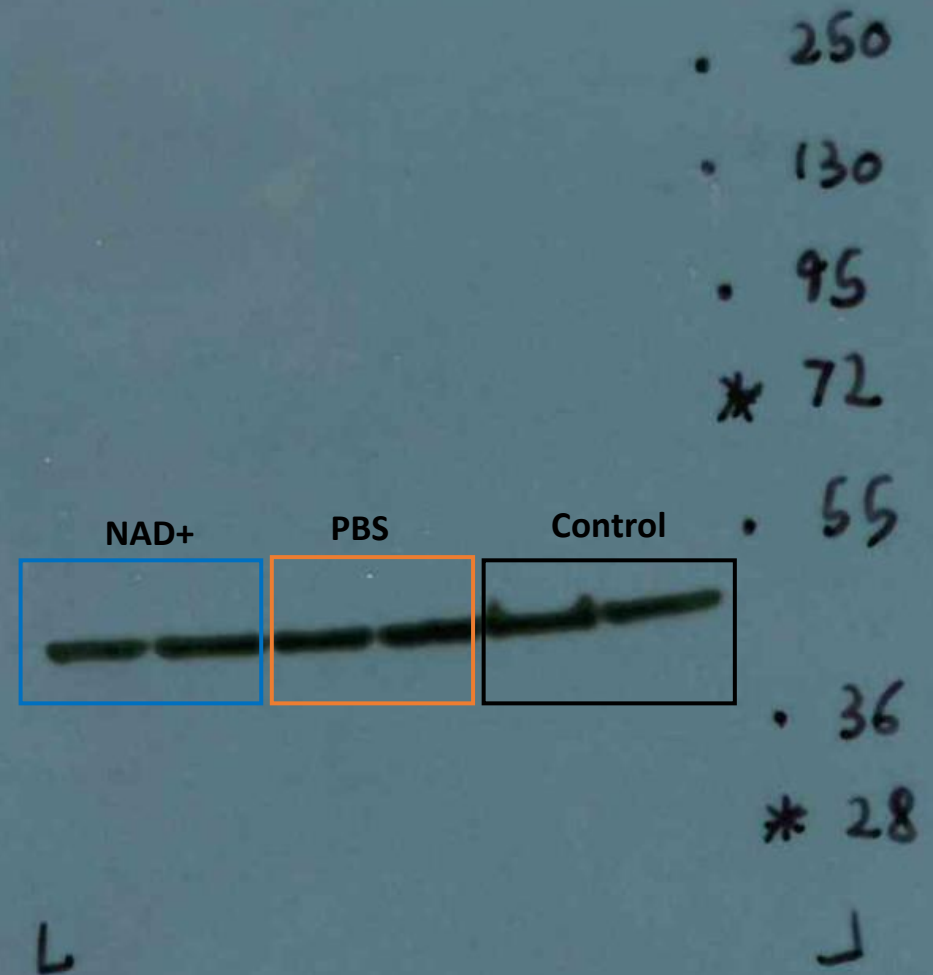

Supplement: Figure 2—source data 2. [file elife-88686-fig2-data2.zip › Figure 2A. Canonical Inflammasome Actin Highlighted.pdf]

# IL-1beta & Casp 1

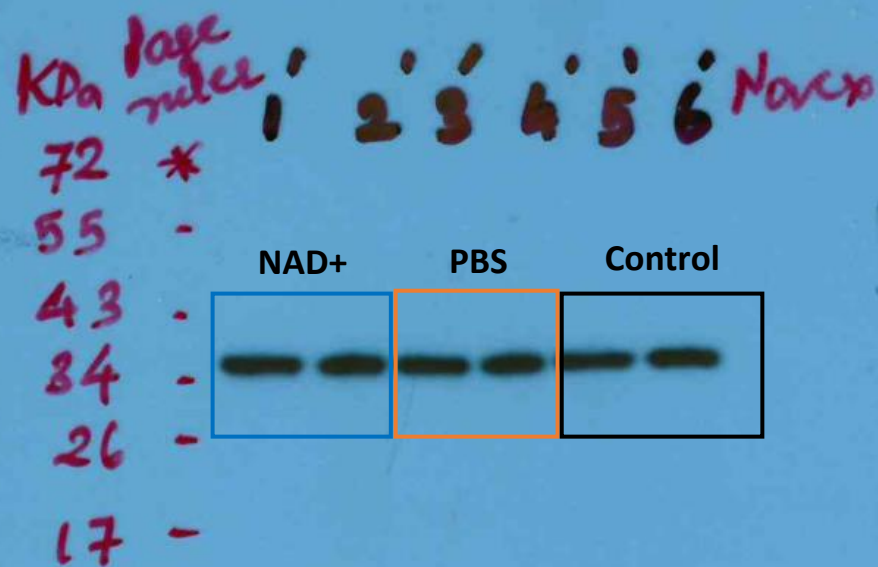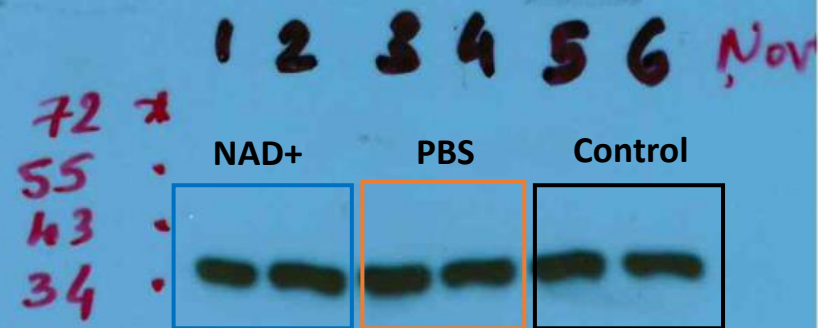

^ B

①

Supplement: Figure 2—source data 2. [file elife-88686-fig2-data2.zip › Figure 2A. Canonical Inflammasome Caspase 1,IL-1beta Highlighted.pdf]

# GSDMD

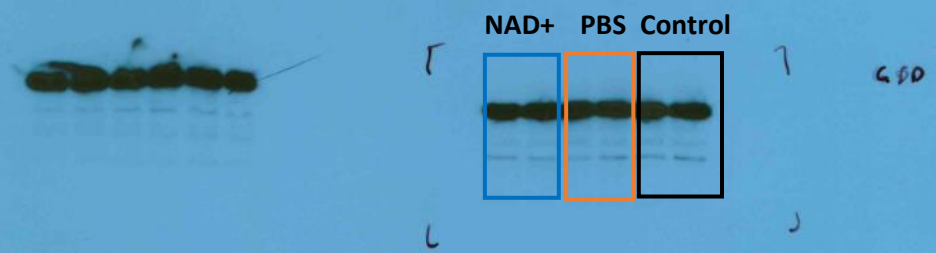

# Pro Casp 11 & Casp 11

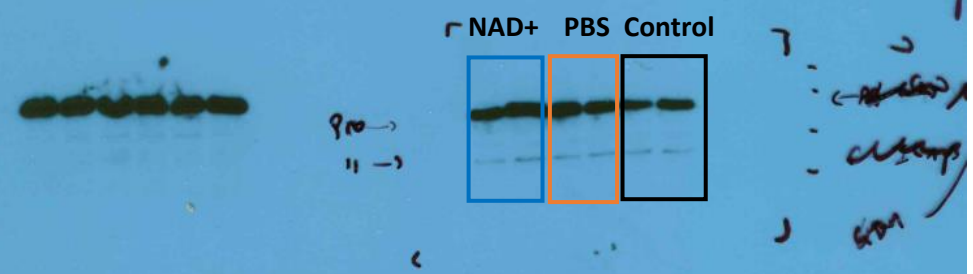

Supplement: Figure 2—source data 2. [file elife-88686-fig2-data2.zip › Figure 2A. Canonical Inflammasome GSDMD, Pro-Casp 11, Casp 11 Highlighted.pdf]

# Casp 1

NAD+    PBS    Control

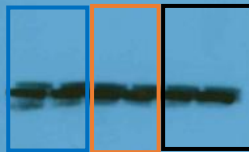

# NLRP3

NAD+    PBS    Control

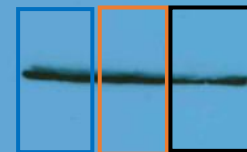

Supplement: Figure 2—source data 2. [file elife-88686-fig2-data2.zip › Figure 2A. Canonical Inflammasome NLRP3 & Pro-Caspase 1 Highlighted.pdf]

# Actin

NAD+

PBS

Control

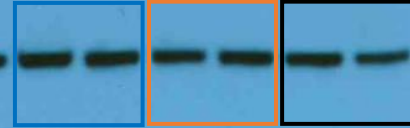

Supplement: Figure 2—source data 2. [file elife-88686-fig2-data2.zip › Figure 2A. Non Canonical Inflammasome Actin Highlighted.pdf]

# Casp 1

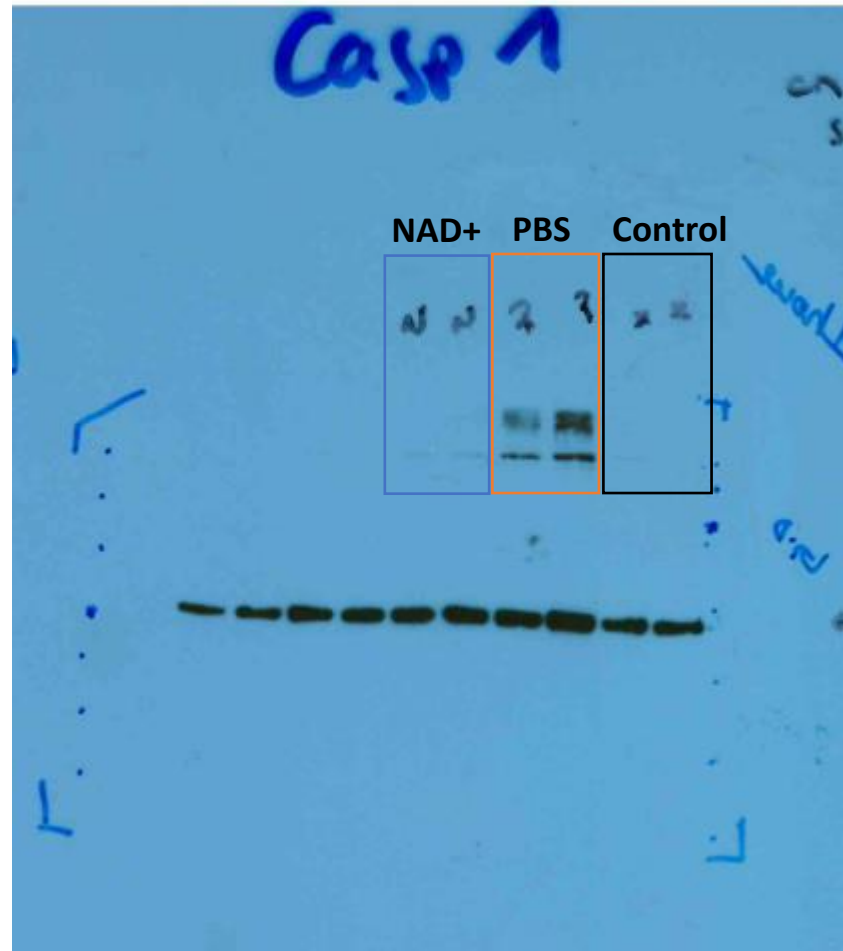

Supplement: Figure 2—source data 2. [file elife-88686-fig2-data2.zip › Figure 2A. Non Canonical Inflammasome Caspase 1 Highlighted.pdf]

# GSDMD

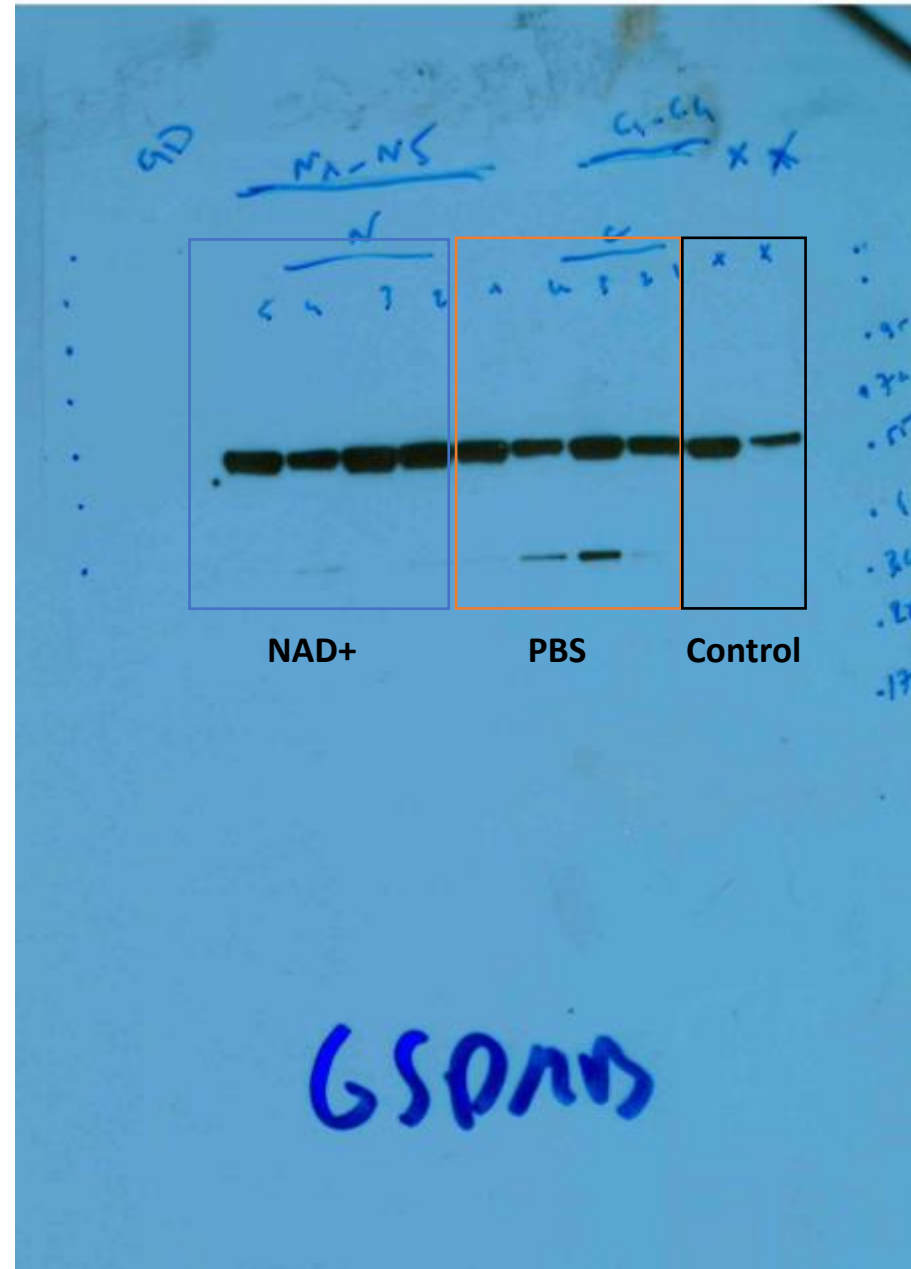

Supplement: Figure 2—source data 2. [file elife-88686-fig2-data2.zip › Figure 2A. Non Canonical Inflammasome GSDMD Highlighted.pdf]

# IL-1beta

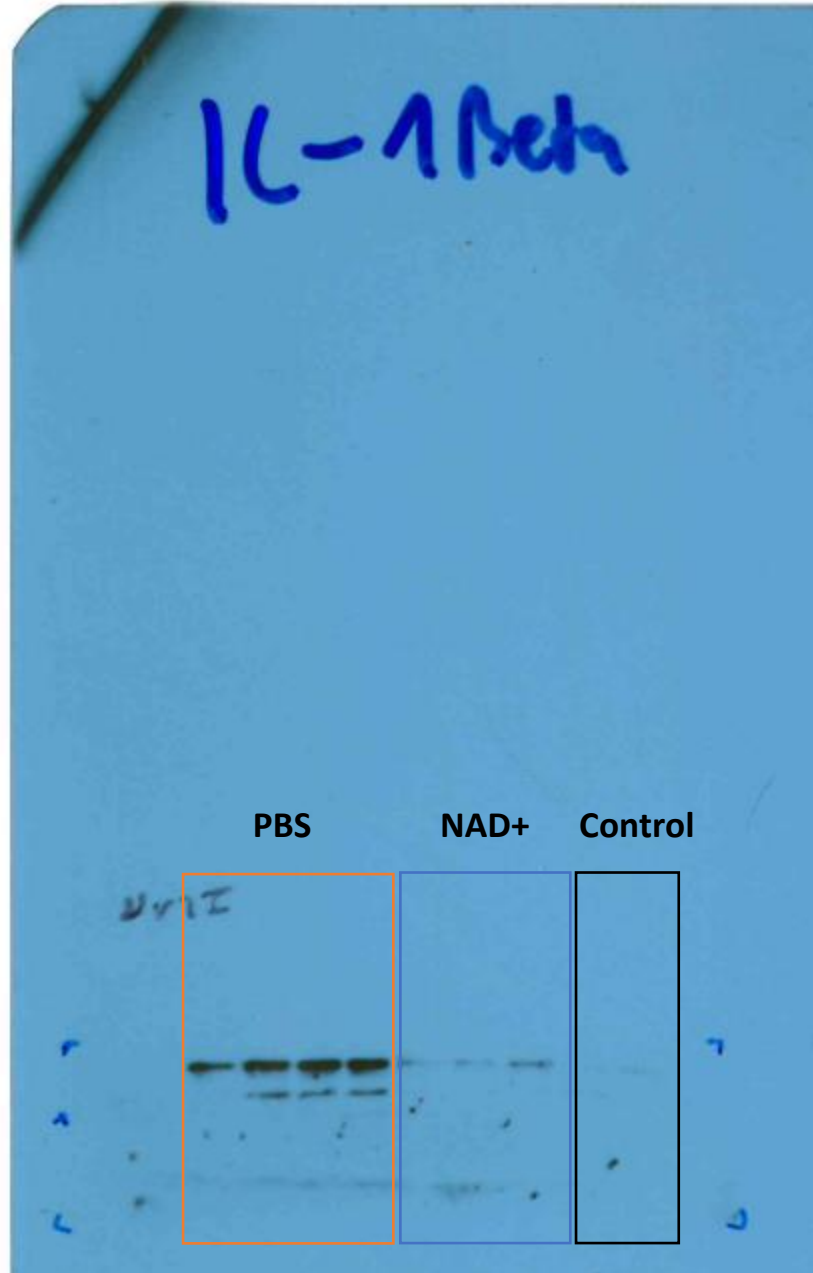

Supplement: Figure 2—source data 2. [file elife-88686-fig2-data2.zip › Figure 2A. Non Canonical Inflammasome IL-1beta Highlighted.pdf]

# NLRP3

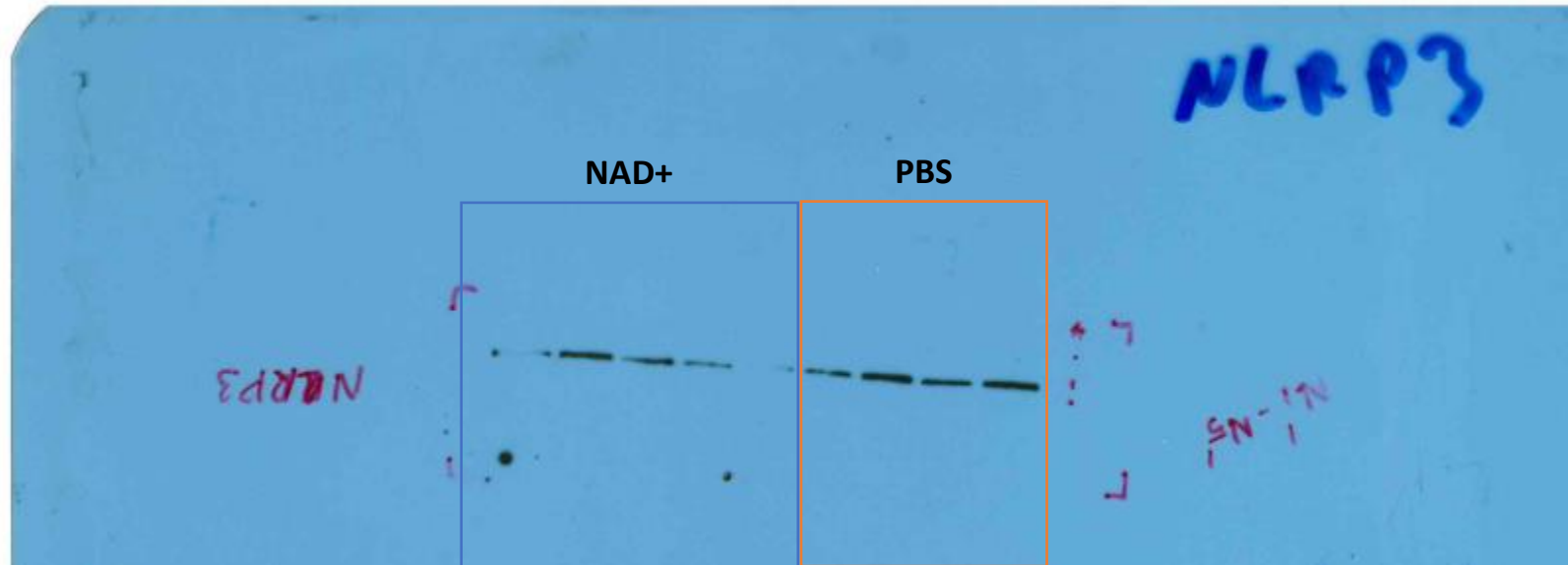

Supplement: Figure 2—source data 2. [file elife-88686-fig2-data2.zip › Figure 2A. Non Canonical Inflammasome NLRP3 Highlighted.pdf]

# Pro Casp 1

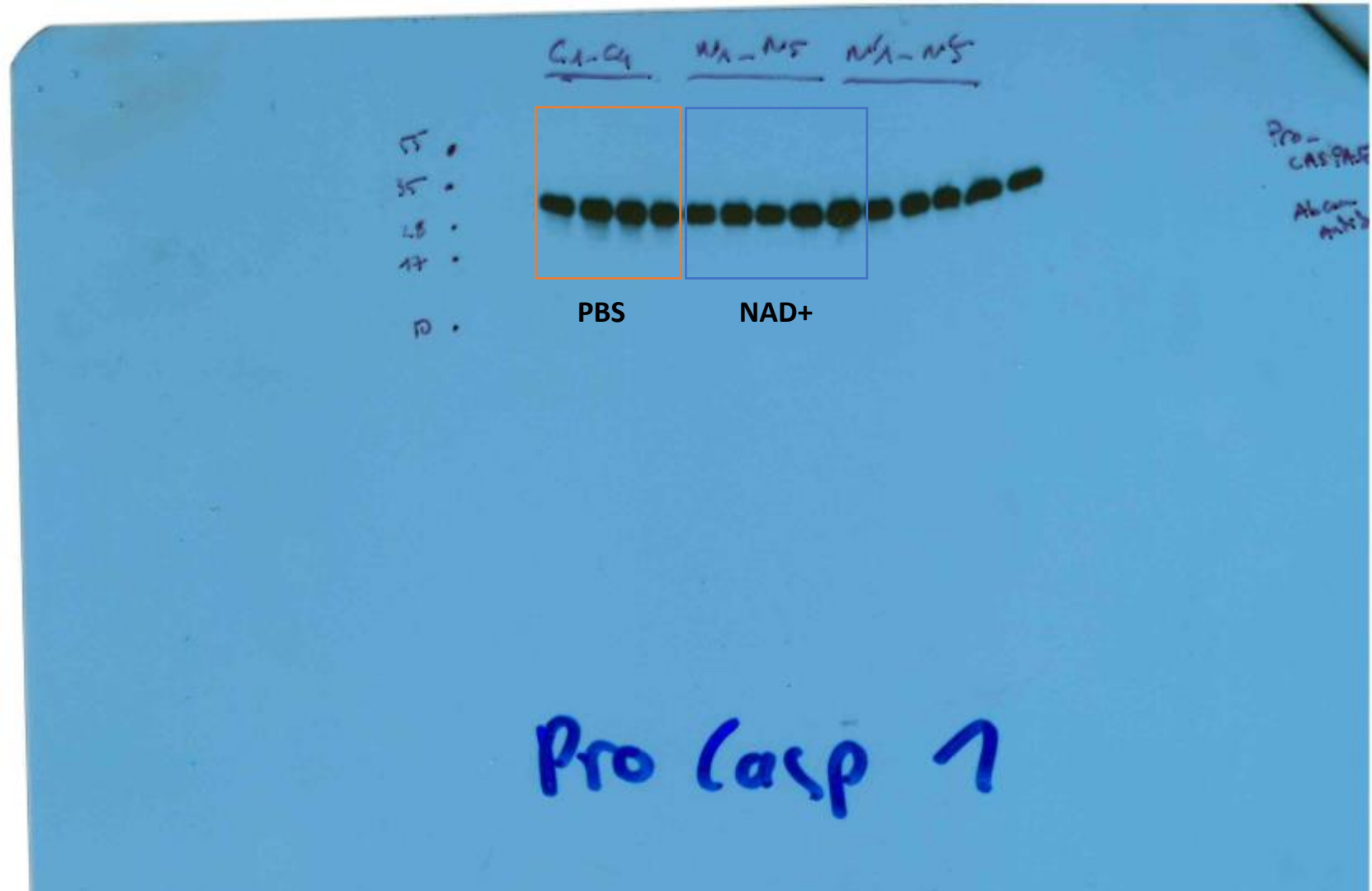

Supplement: Figure 2—source data 2. [file elife-88686-fig2-data2.zip › Figure 2A. Non Canonical Inflammasome Pro Casp 1 Highlighted.pdf]

# Casp 11

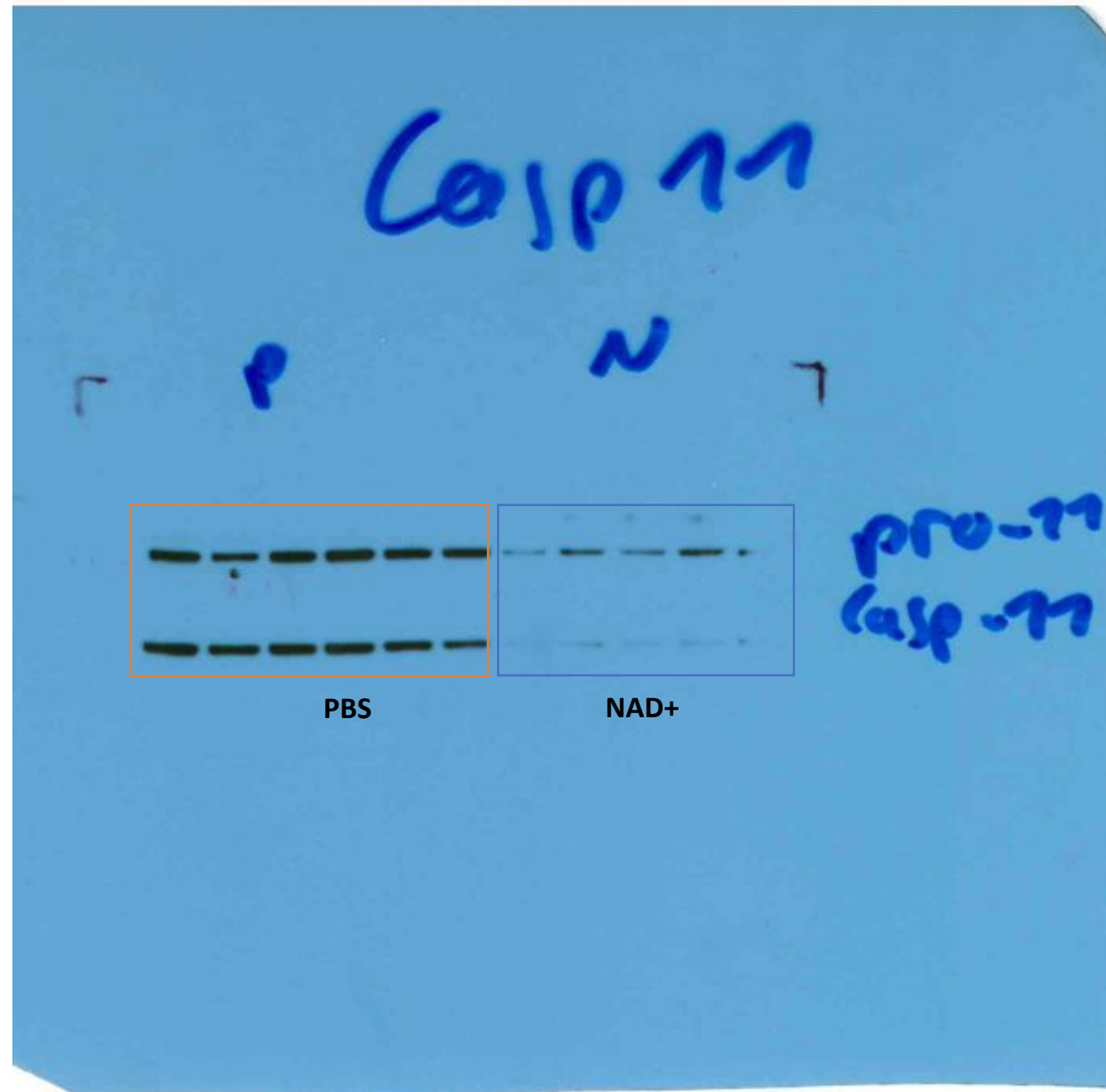

Supplement: Figure 2—source data 2. [file elife-88686-fig2-data2.zip › Figure 2A. Non Canonical Inflammasome Pro-Caspase 11 and Caspase 11.pdf]

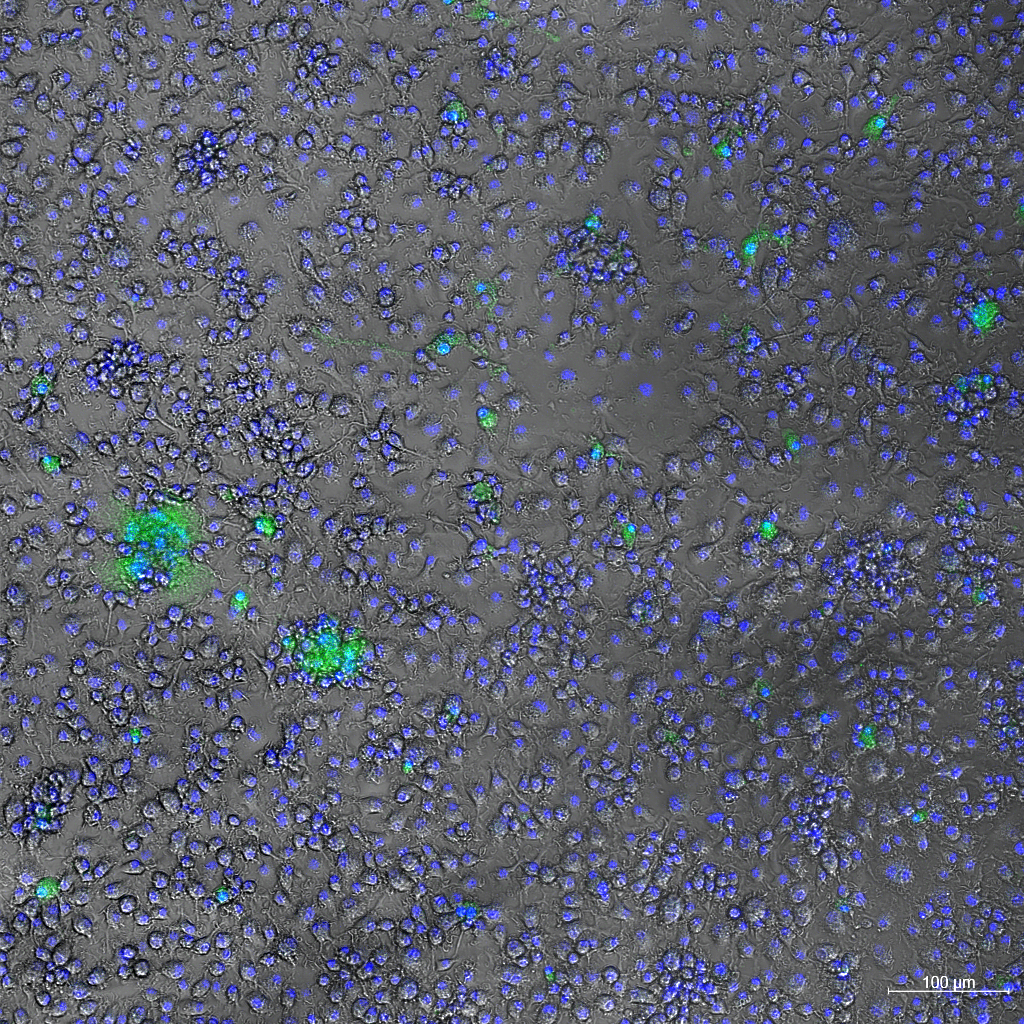

Supplement: Figure 2—source data 4. [file elife-88686-fig2-data4.zip › NAD_16h_10x.tif]

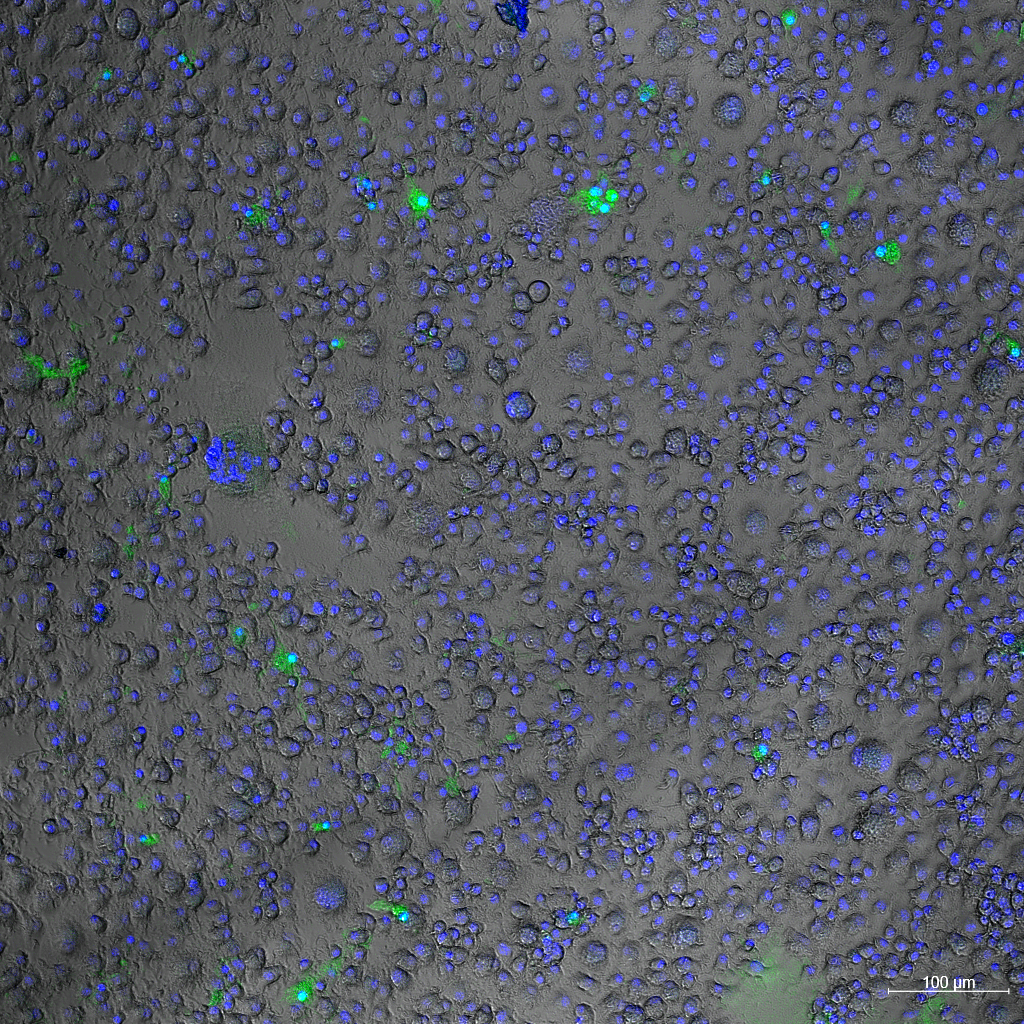

Supplement: Figure 2—source data 4. [file elife-88686-fig2-data4.zip › NAD_4h_10x.tif]

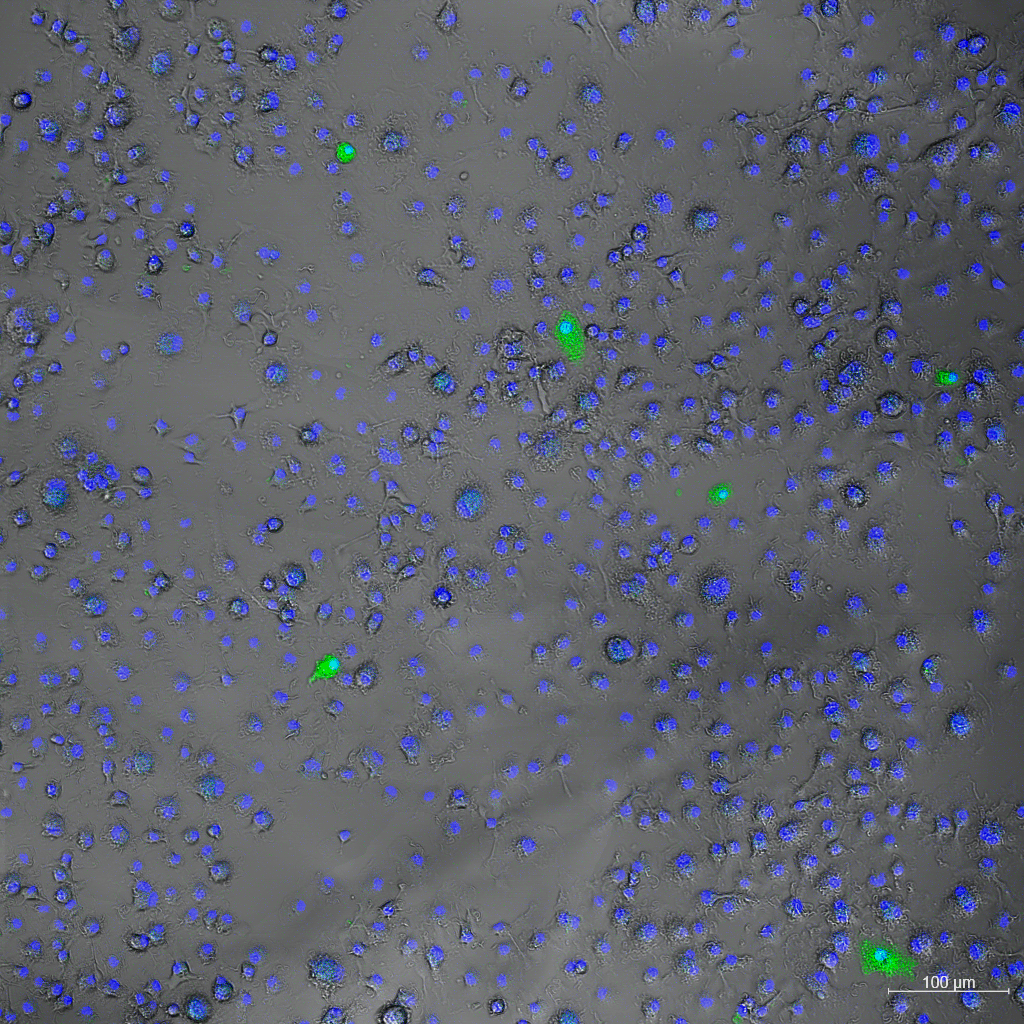

Supplement: Figure 2—source data 4. [file elife-88686-fig2-data4.zip › PBS_16h_10x.tif]

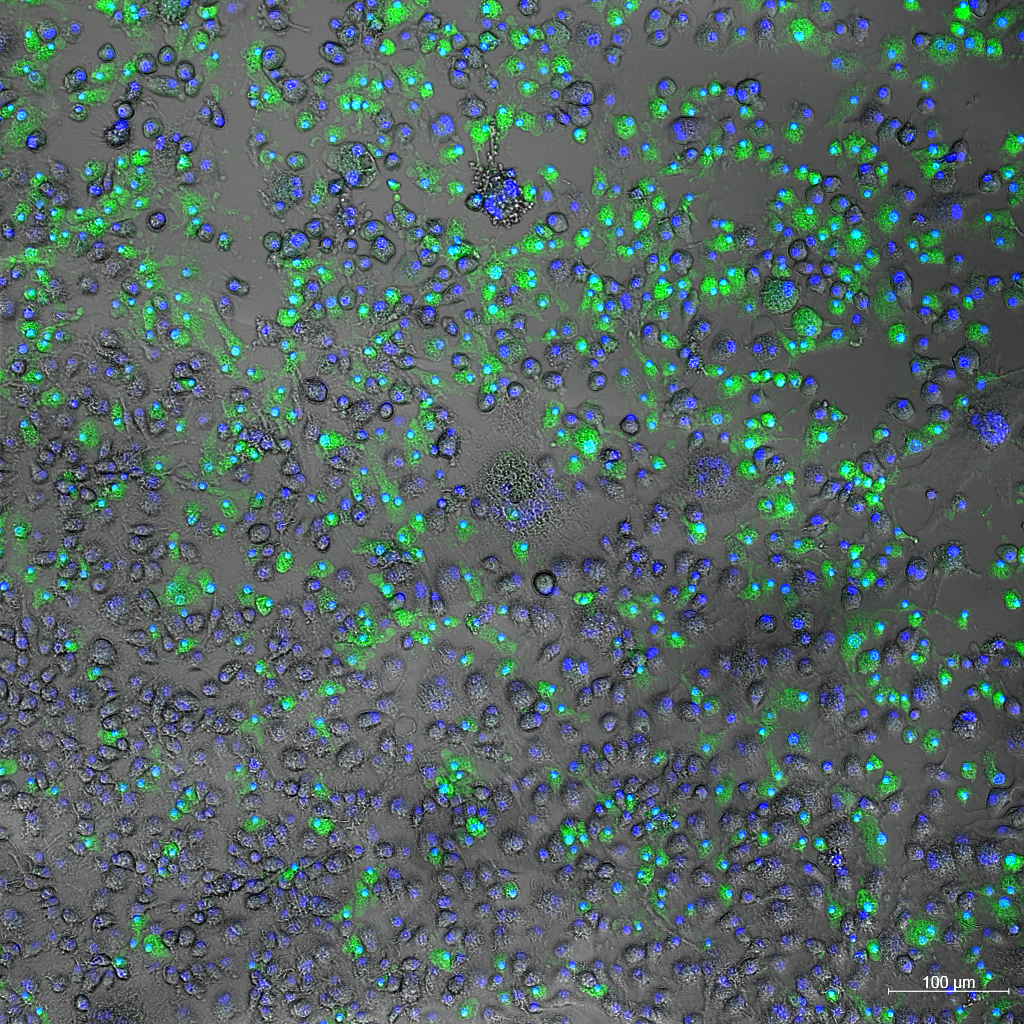

Supplement: Figure 2—source data 4. [file elife-88686-fig2-data4.zip › PBS_4h_10x.tif]

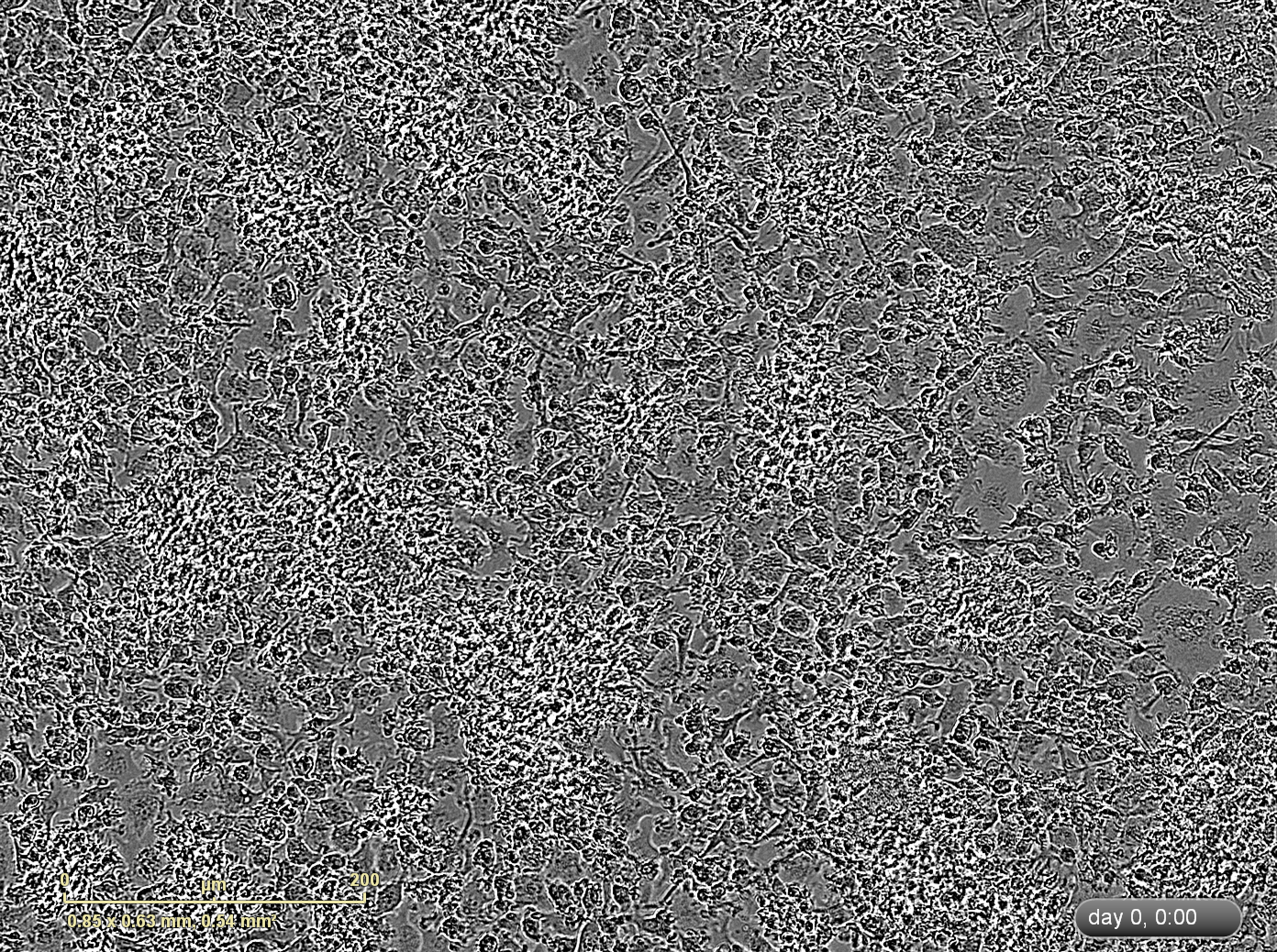

Supplement: Figure 2—source data 5. [file elife-88686-fig2-data5.zip › NAD 0h.tif]

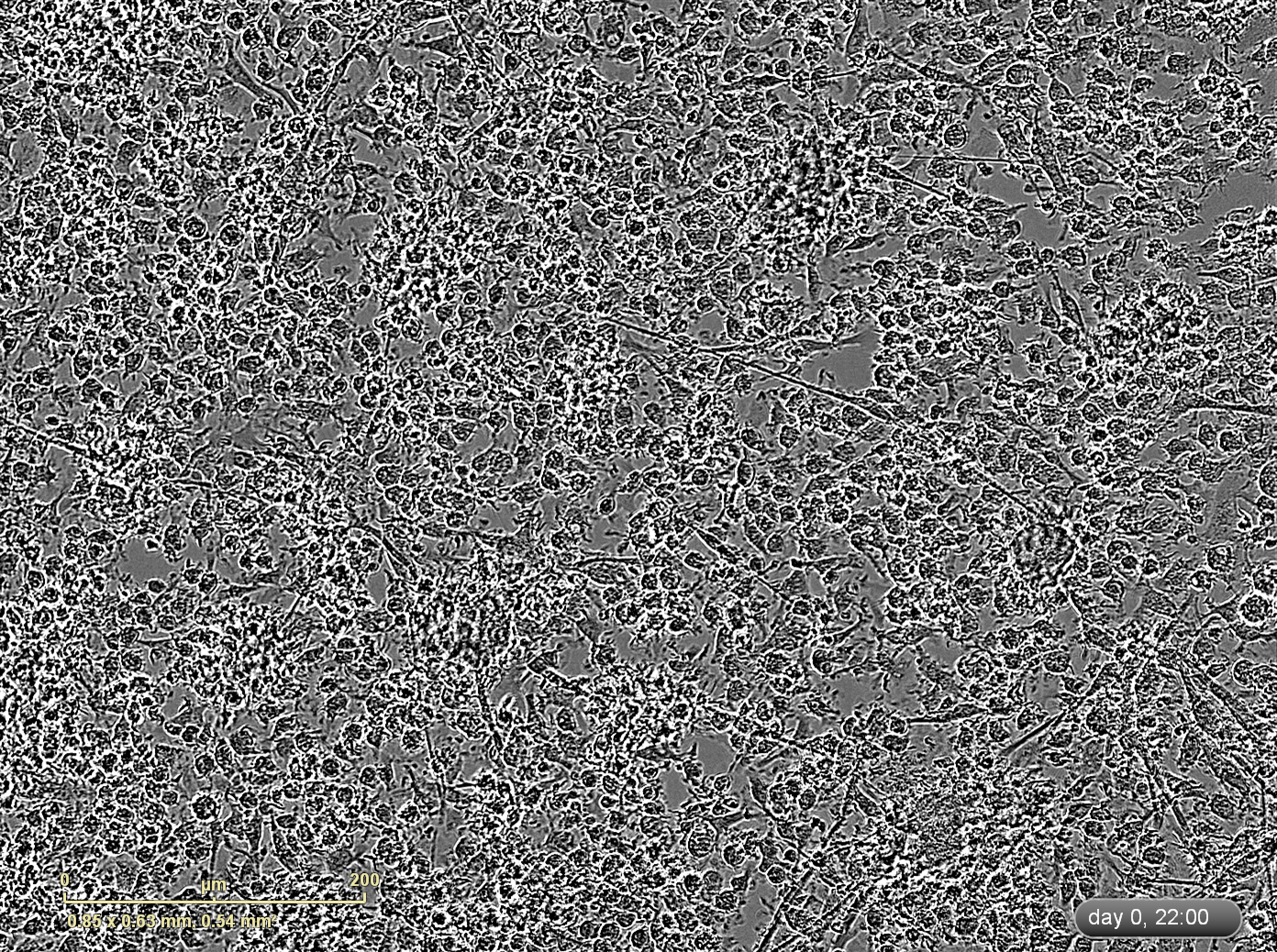

Supplement: Figure 2—source data 5. [file elife-88686-fig2-data5.zip › NAD 24h.tif]

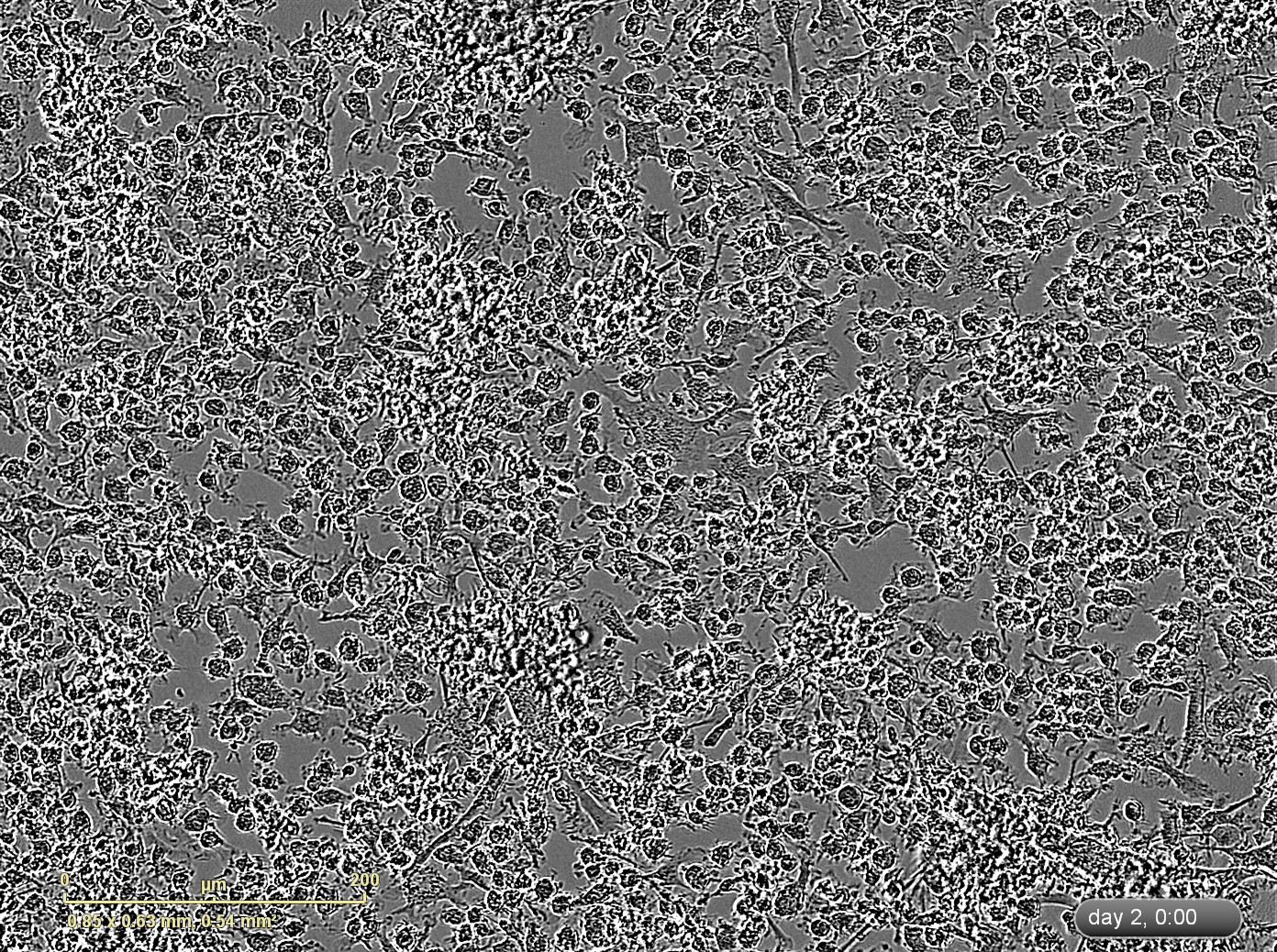

Supplement: Figure 2—source data 5. [file elife-88686-fig2-data5.zip › NAD 48h.tif]

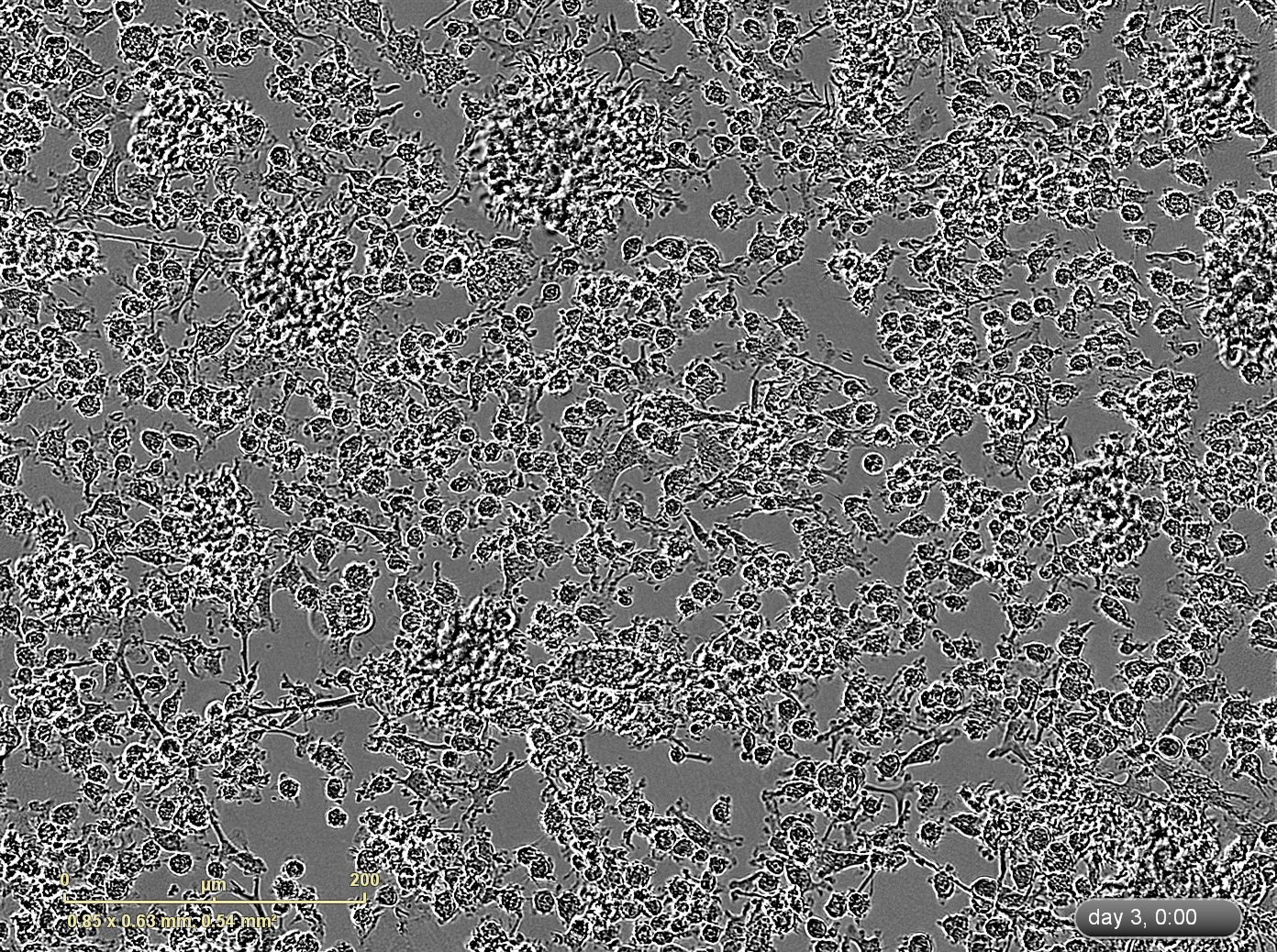

Supplement: Figure 2—source data 5. [file elife-88686-fig2-data5.zip › NAD 72.tif]

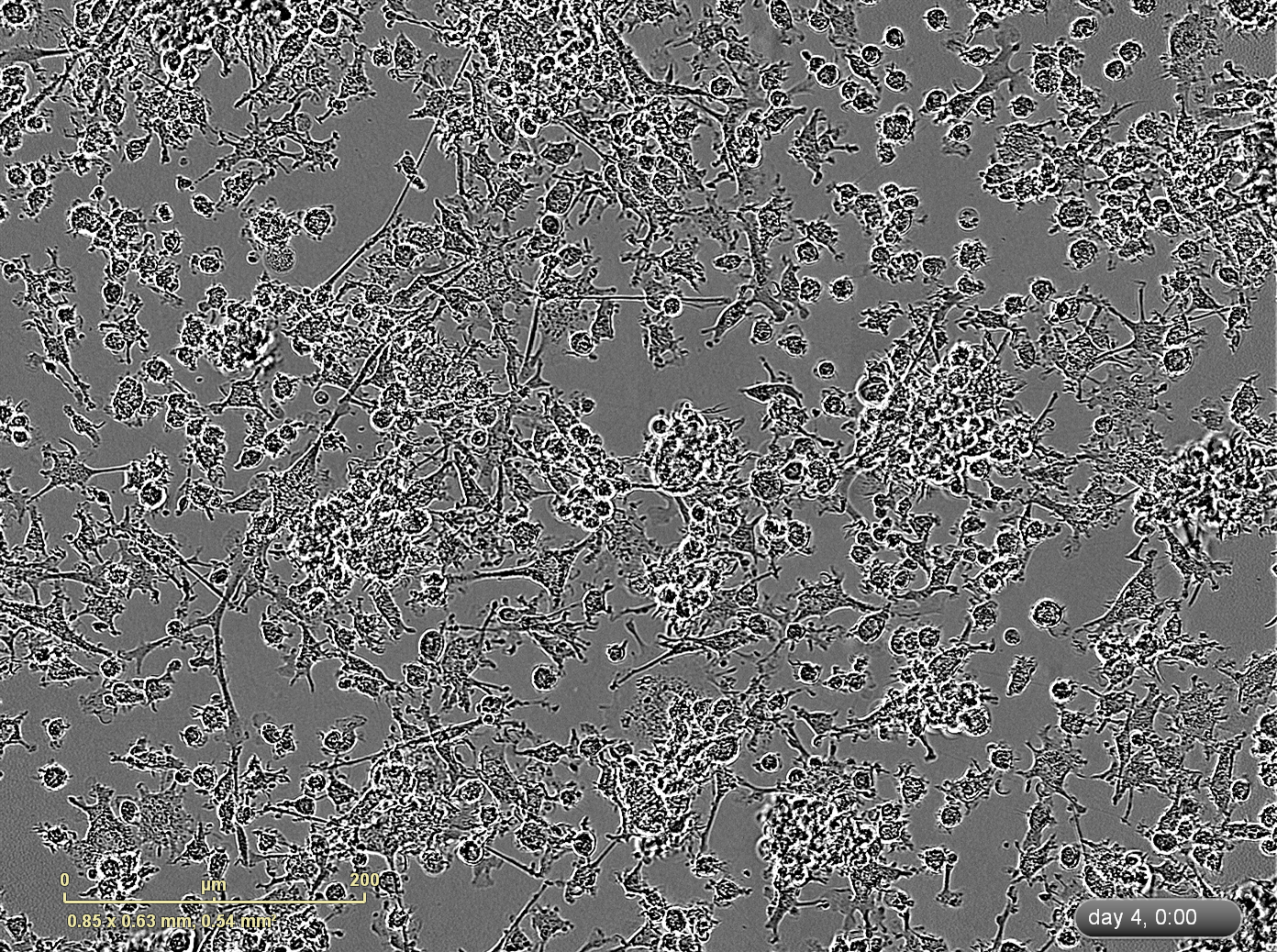

Supplement: Figure 2—source data 5. [file elife-88686-fig2-data5.zip › NAD 96h.tif]

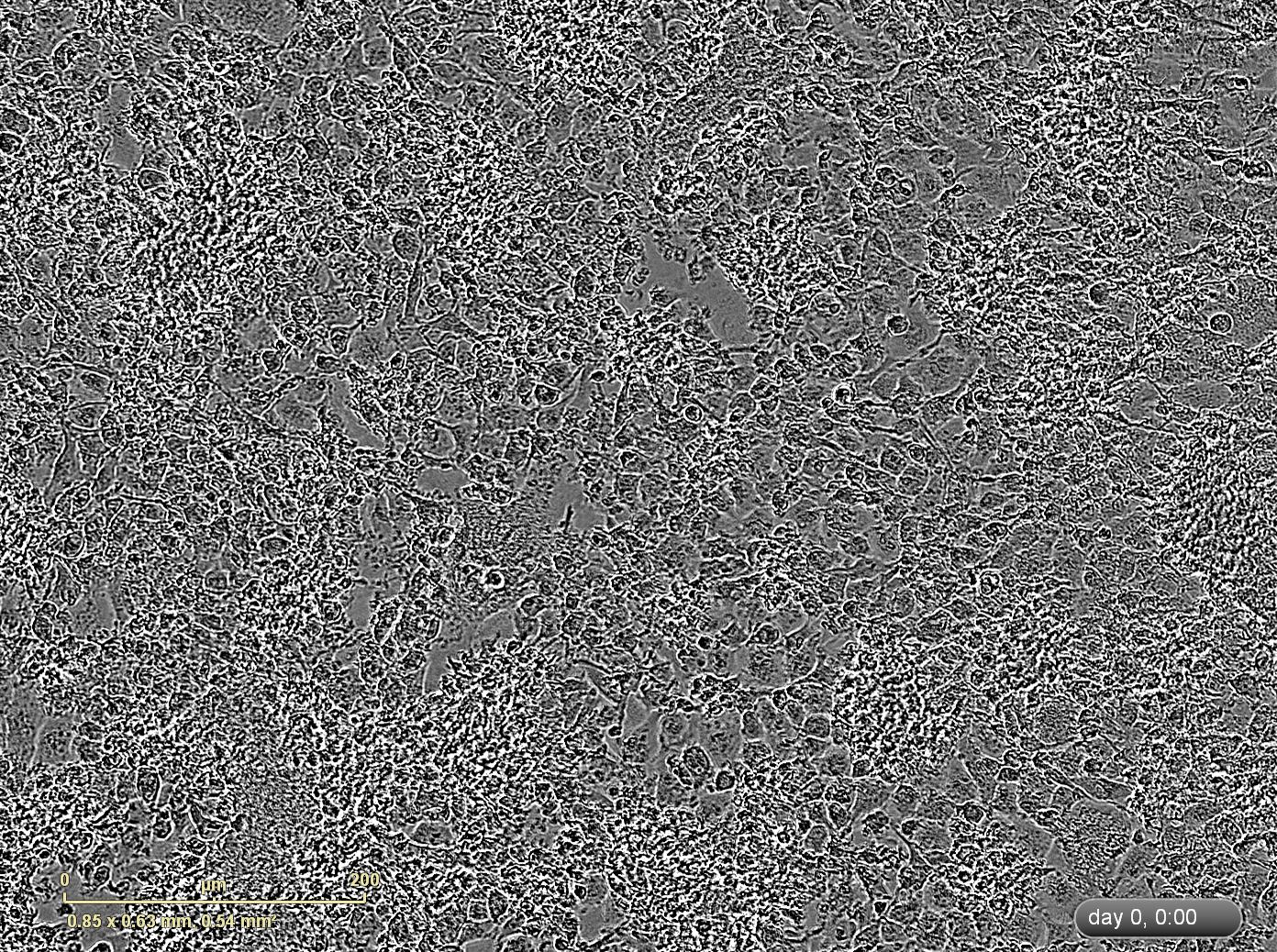

Supplement: Figure 2—source data 5. [file elife-88686-fig2-data5.zip › PBS 0h.tif]

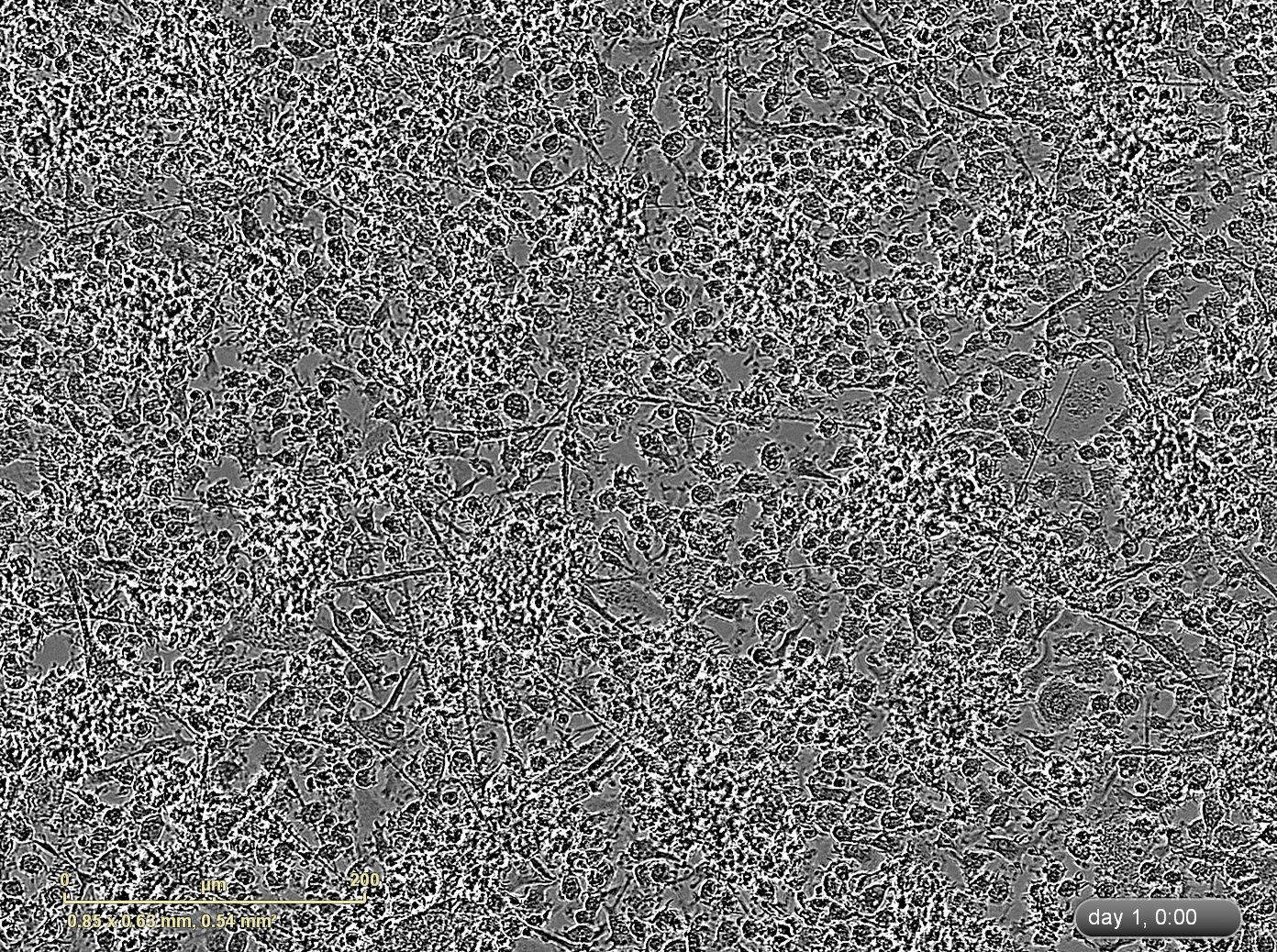

Supplement: Figure 2—source data 5. [file elife-88686-fig2-data5.zip › PBS 24h.tif]

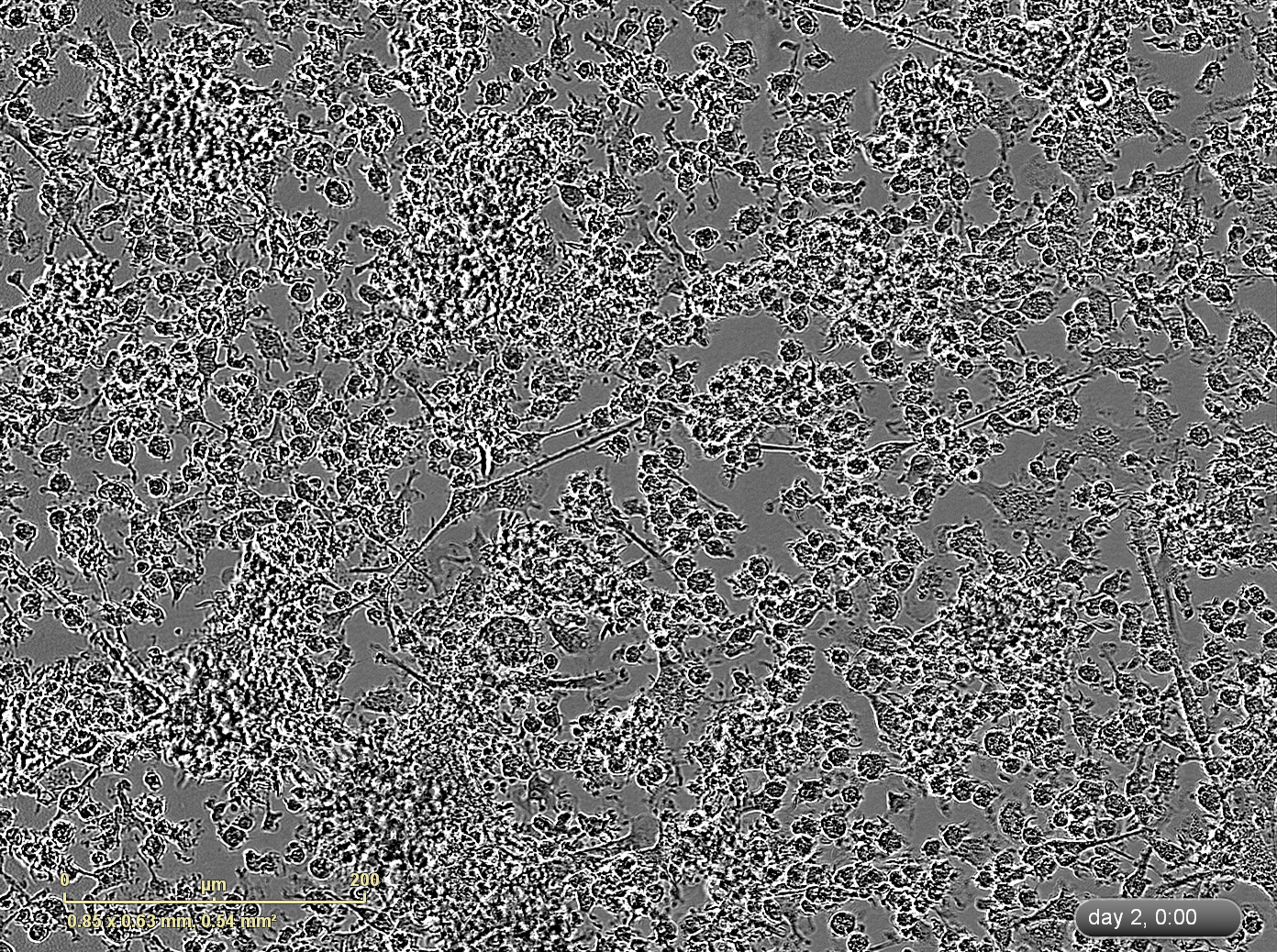

Supplement: Figure 2—source data 5. [file elife-88686-fig2-data5.zip › PBS 48h.tif]

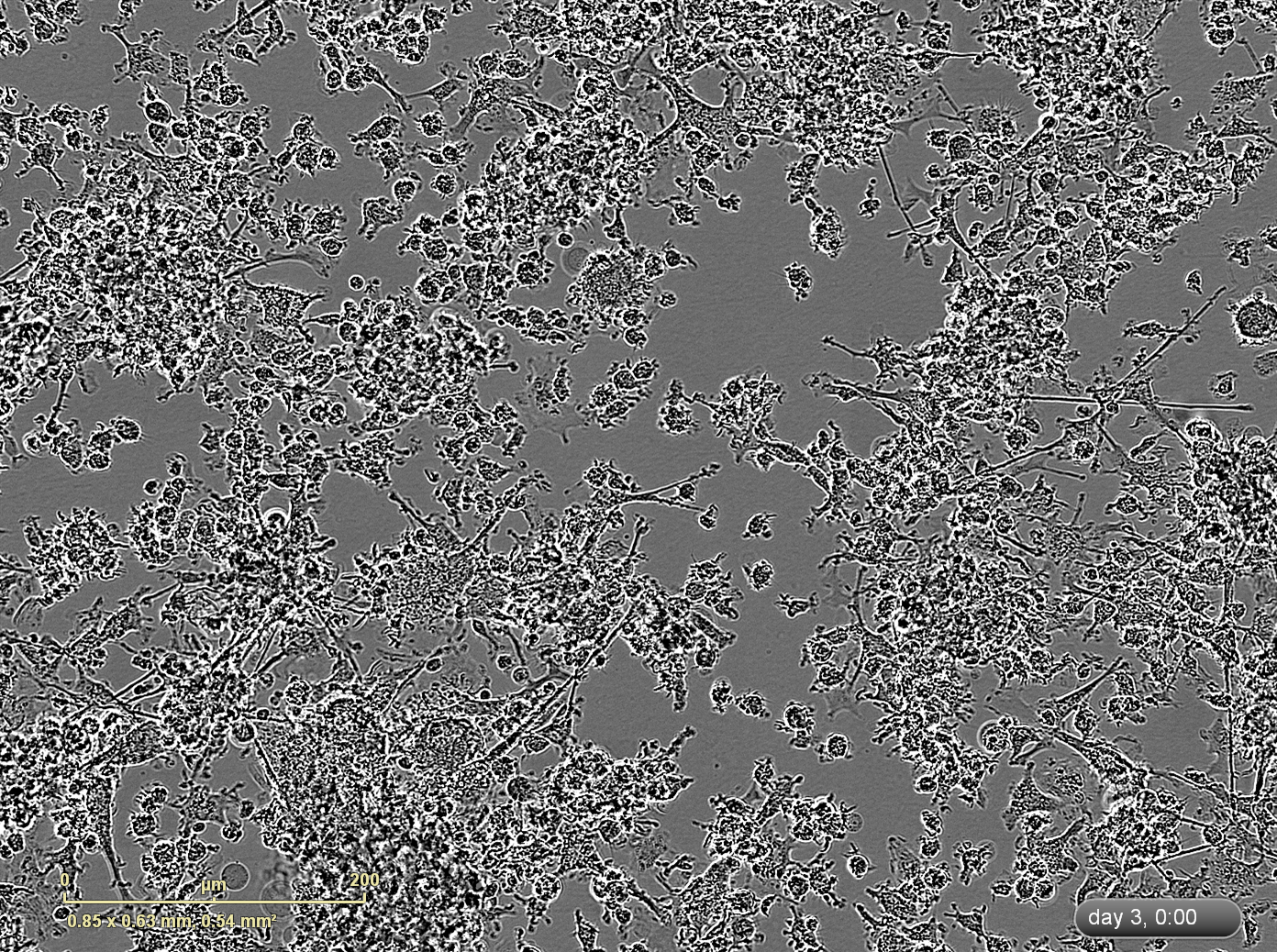

Supplement: Figure 2—source data 5. [file elife-88686-fig2-data5.zip › PBS 72h.tif]

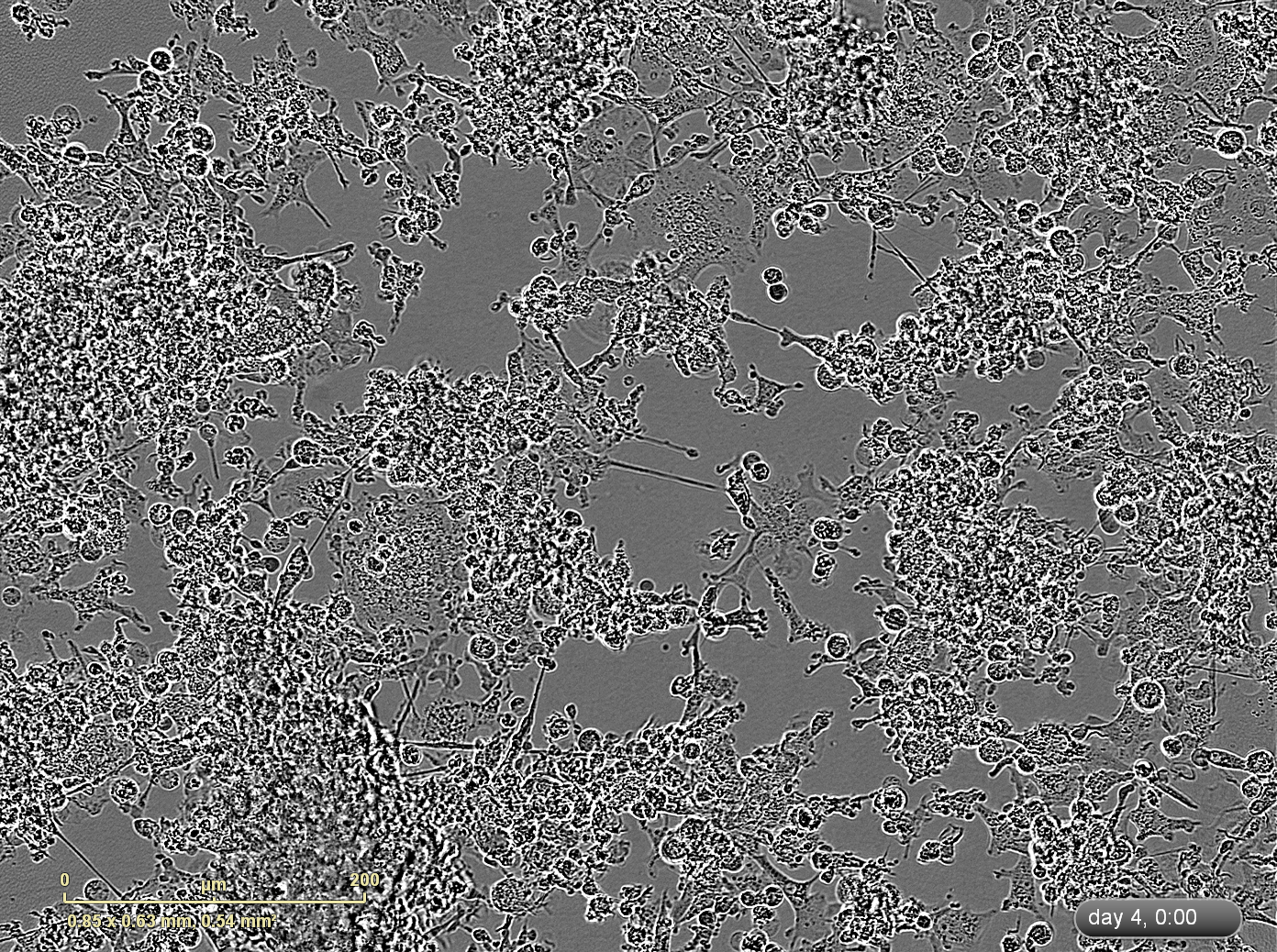

Supplement: Figure 2—source data 5. [file elife-88686-fig2-data5.zip › PBS 96h.tif]

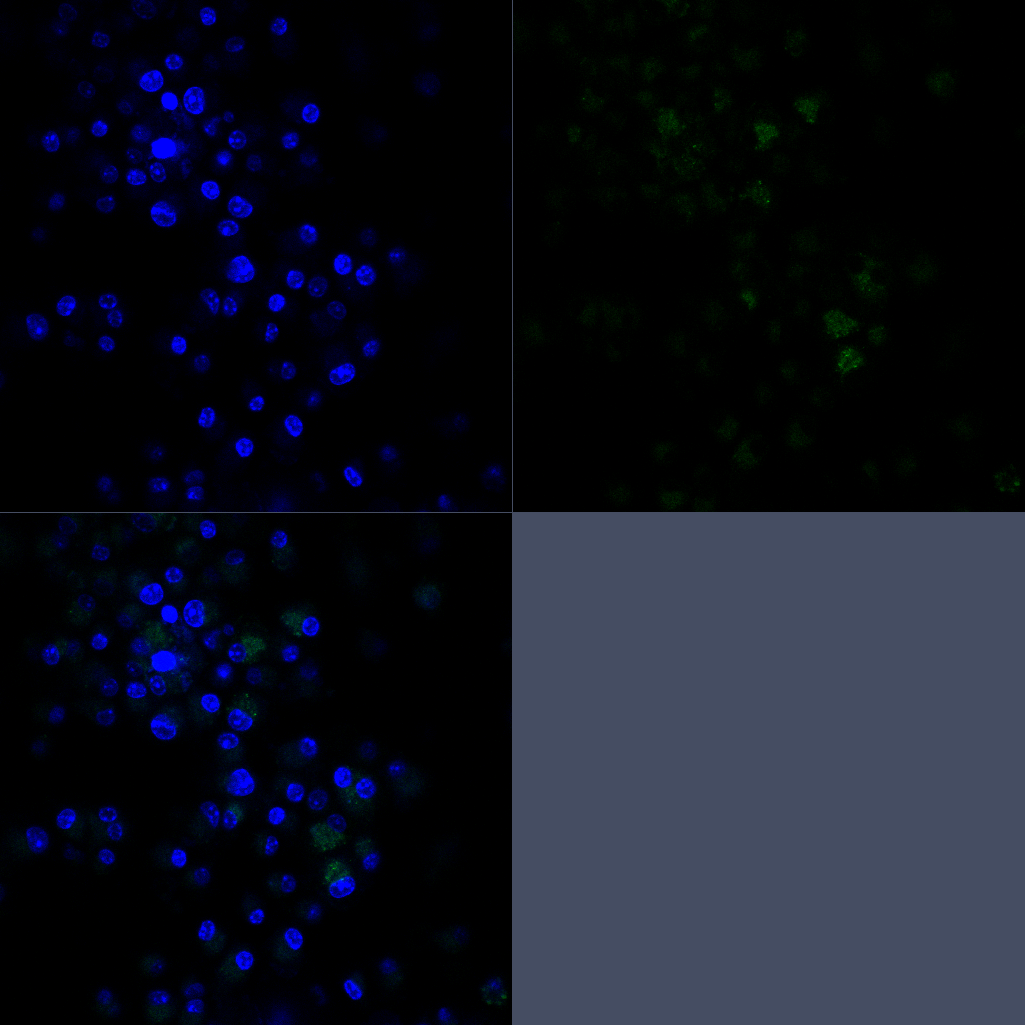

Supplement: Figure 2—source data 6. [file elife-88686-fig2-data6.zip › Figure NAD 63x 1.tif]

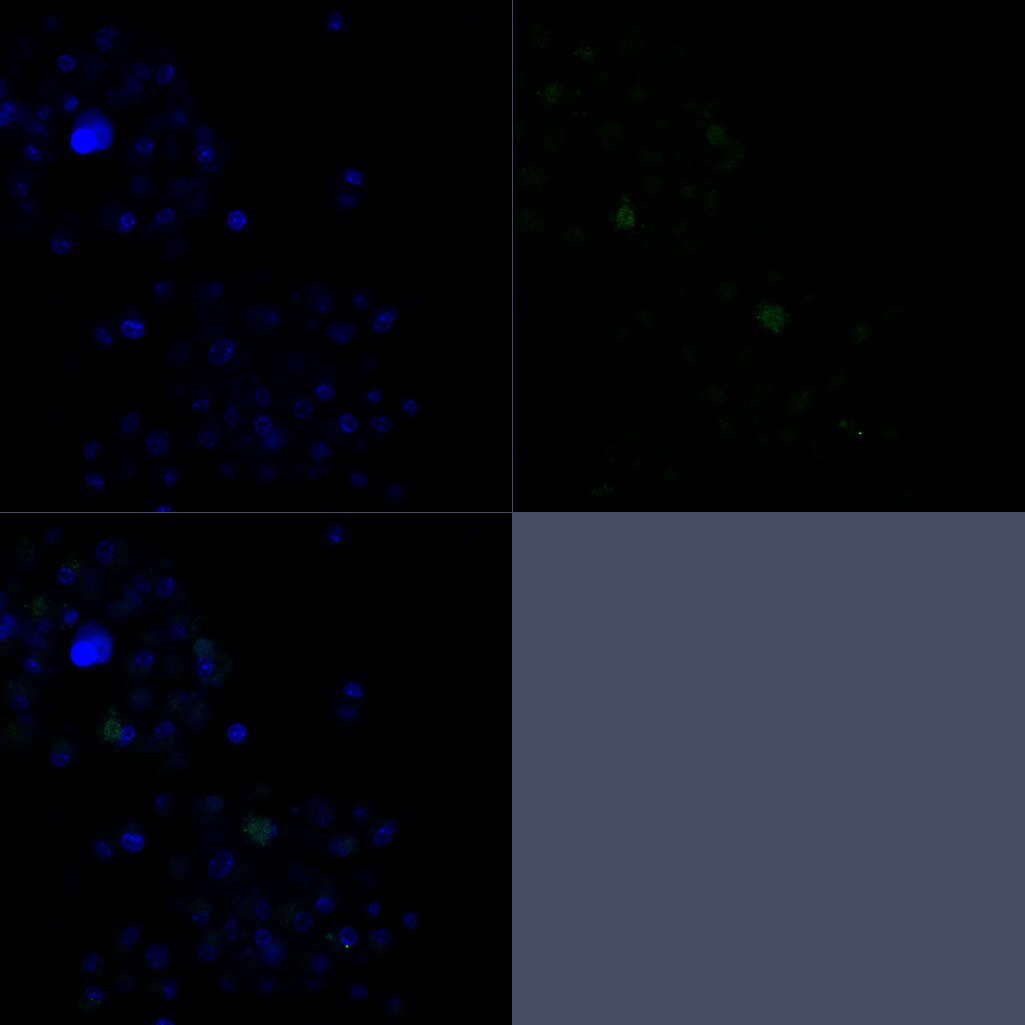

Supplement: Figure 2—source data 6. [file elife-88686-fig2-data6.zip › Figure NAD 63x 2.tif]

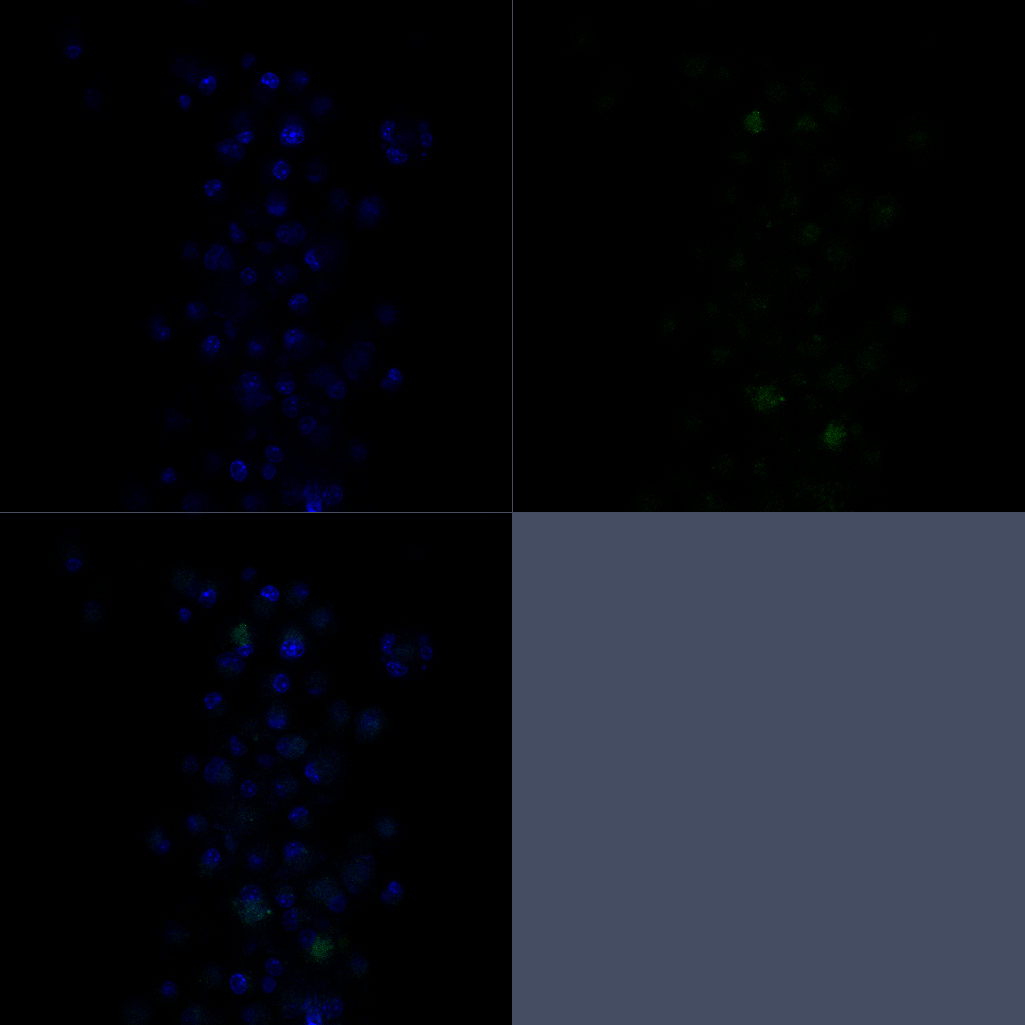

Supplement: Figure 2—source data 6. [file elife-88686-fig2-data6.zip › Figure NAD 63x 3.tif]

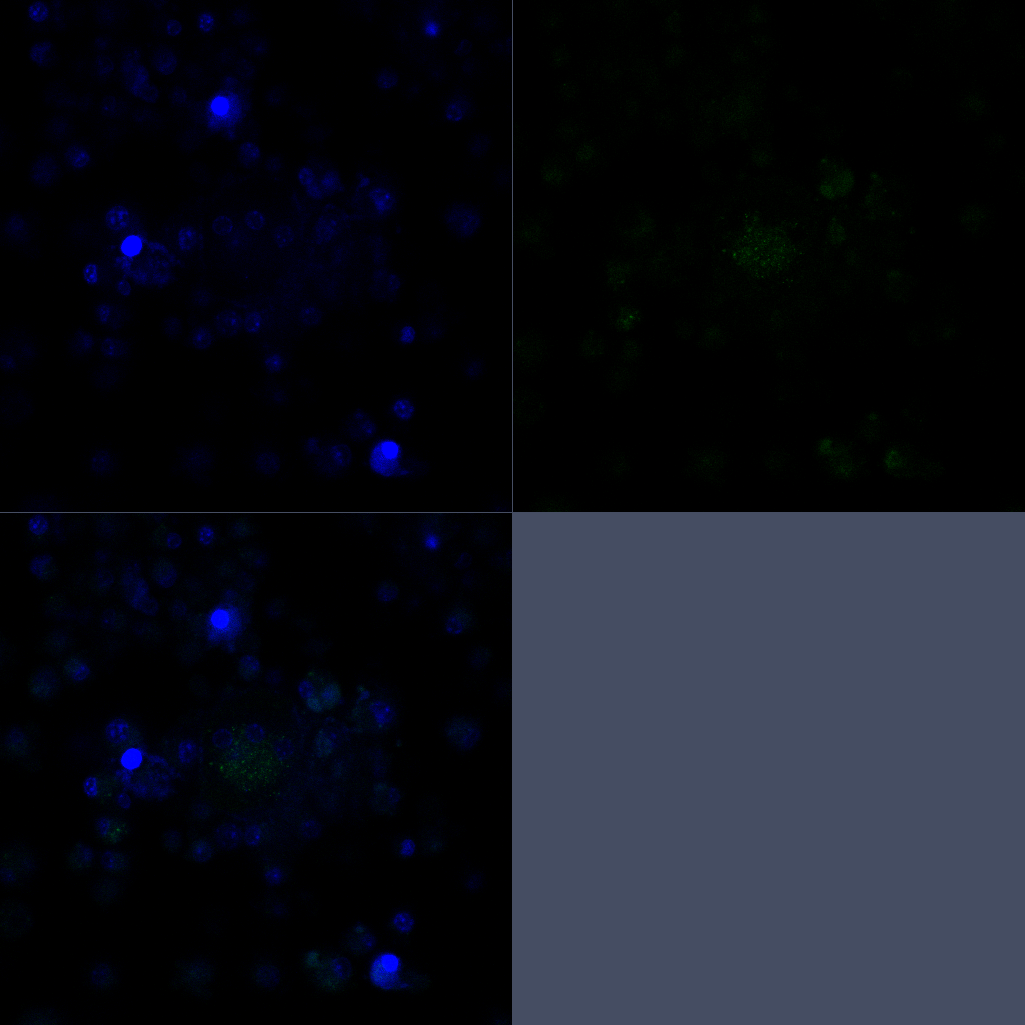

Supplement: Figure 2—source data 6. [file elife-88686-fig2-data6.zip › Figure NAD 63x 4.tif]

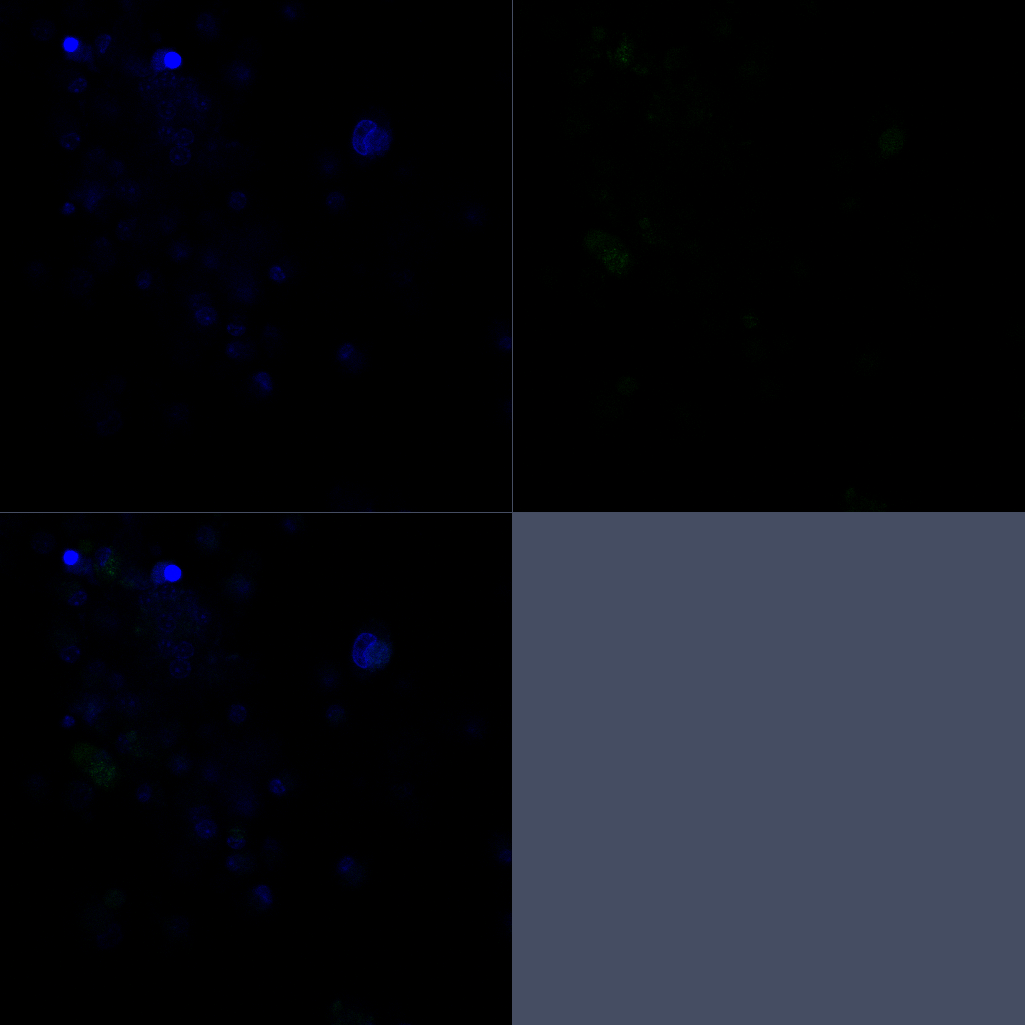

Supplement: Figure 2—source data 6. [file elife-88686-fig2-data6.zip › Figure NAD 63x 5.tif]

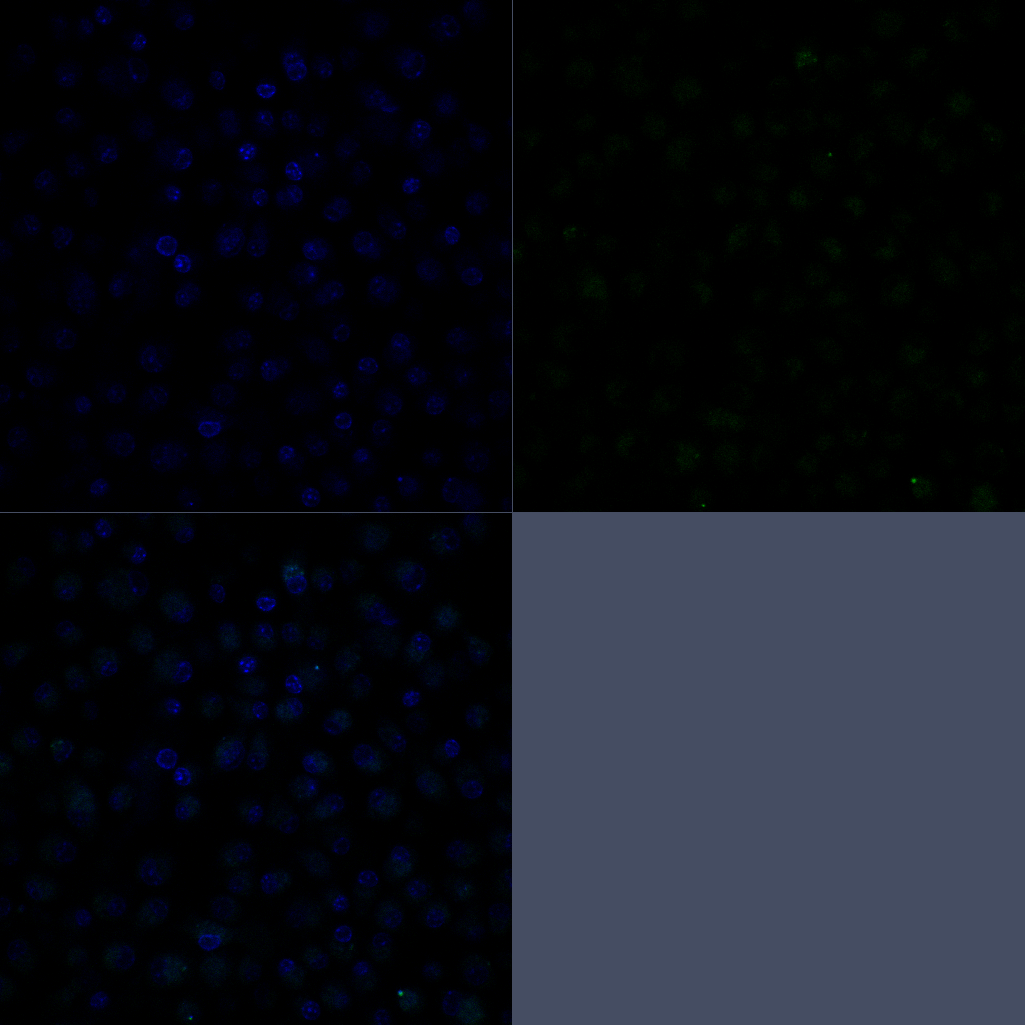

Supplement: Figure 2—source data 6. [file elife-88686-fig2-data6.zip › Figure NO CTB 63x.tif]

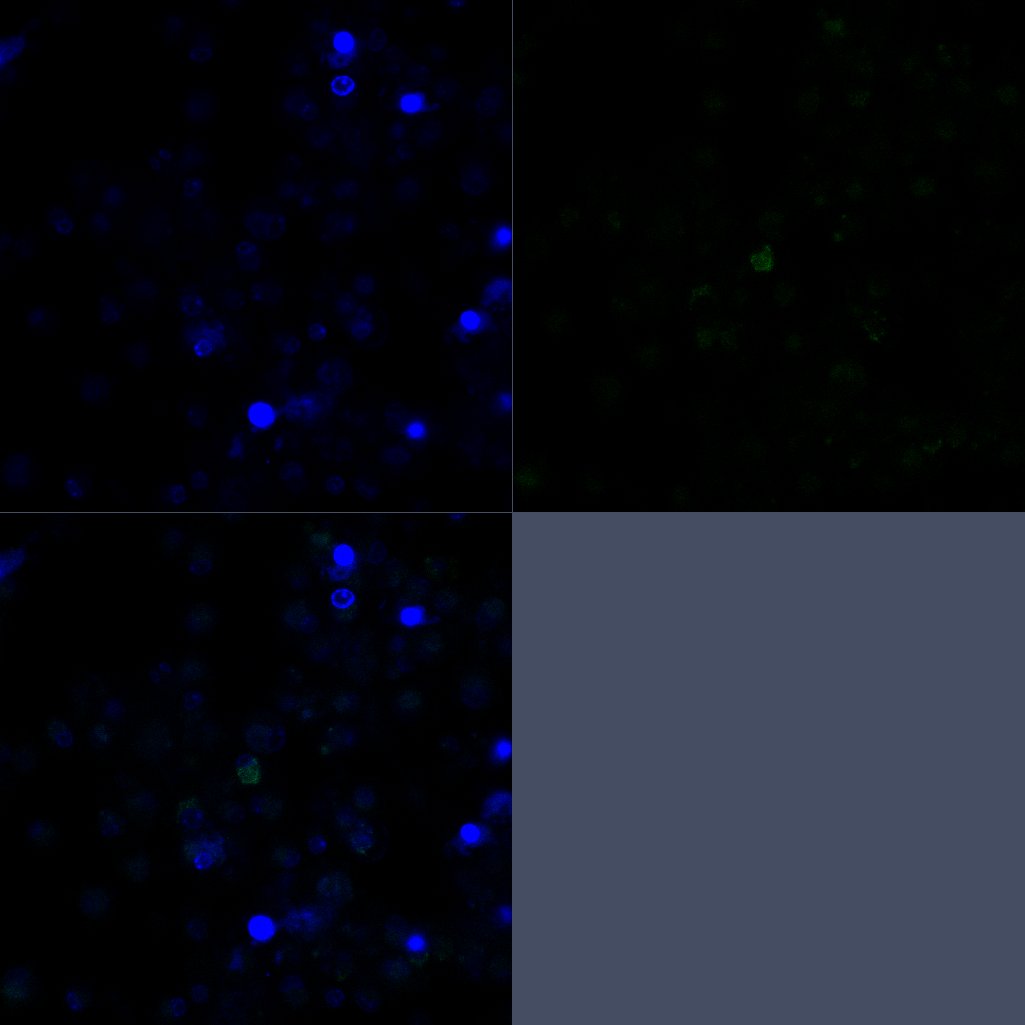

Supplement: Figure 2—source data 6. [file elife-88686-fig2-data6.zip › Figure PBS 63x 1.tif]

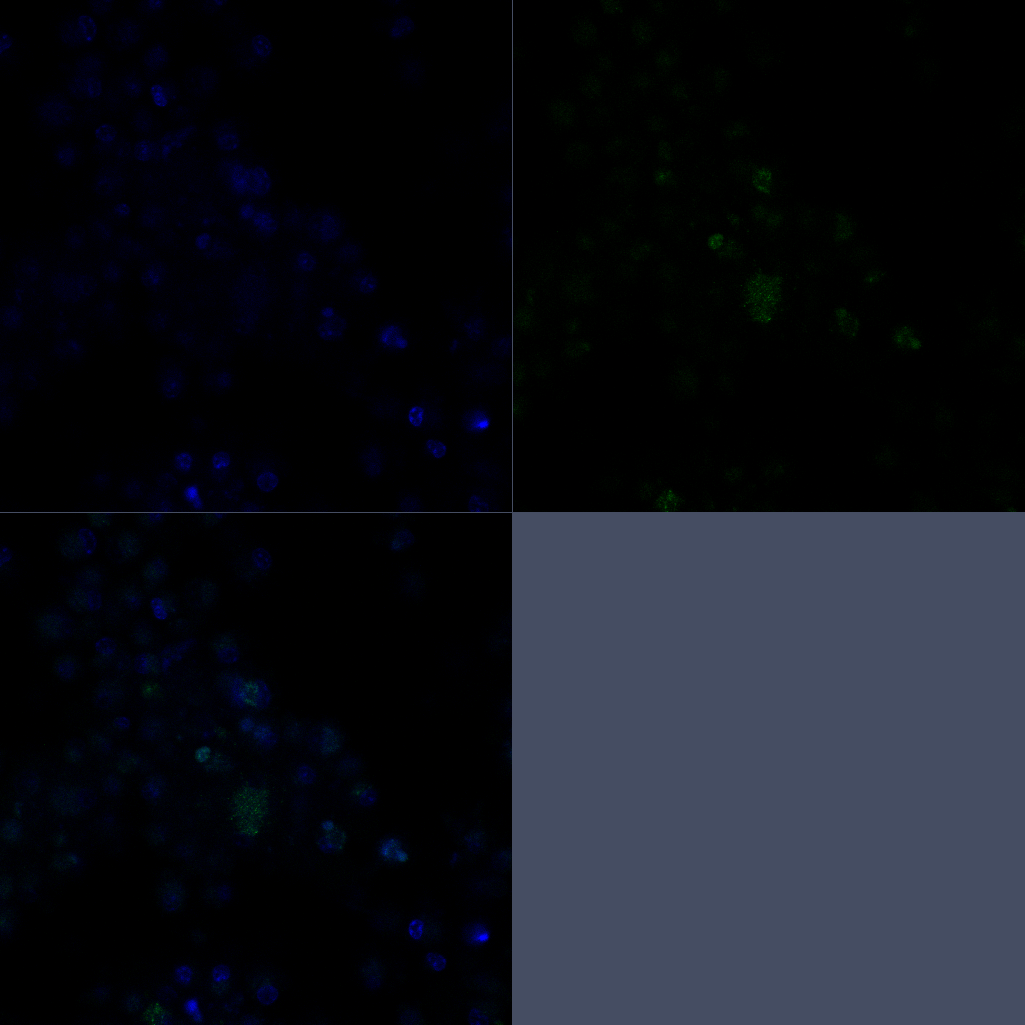

Supplement: Figure 2—source data 6. [file elife-88686-fig2-data6.zip › Figure PBS 63x 2.tif]

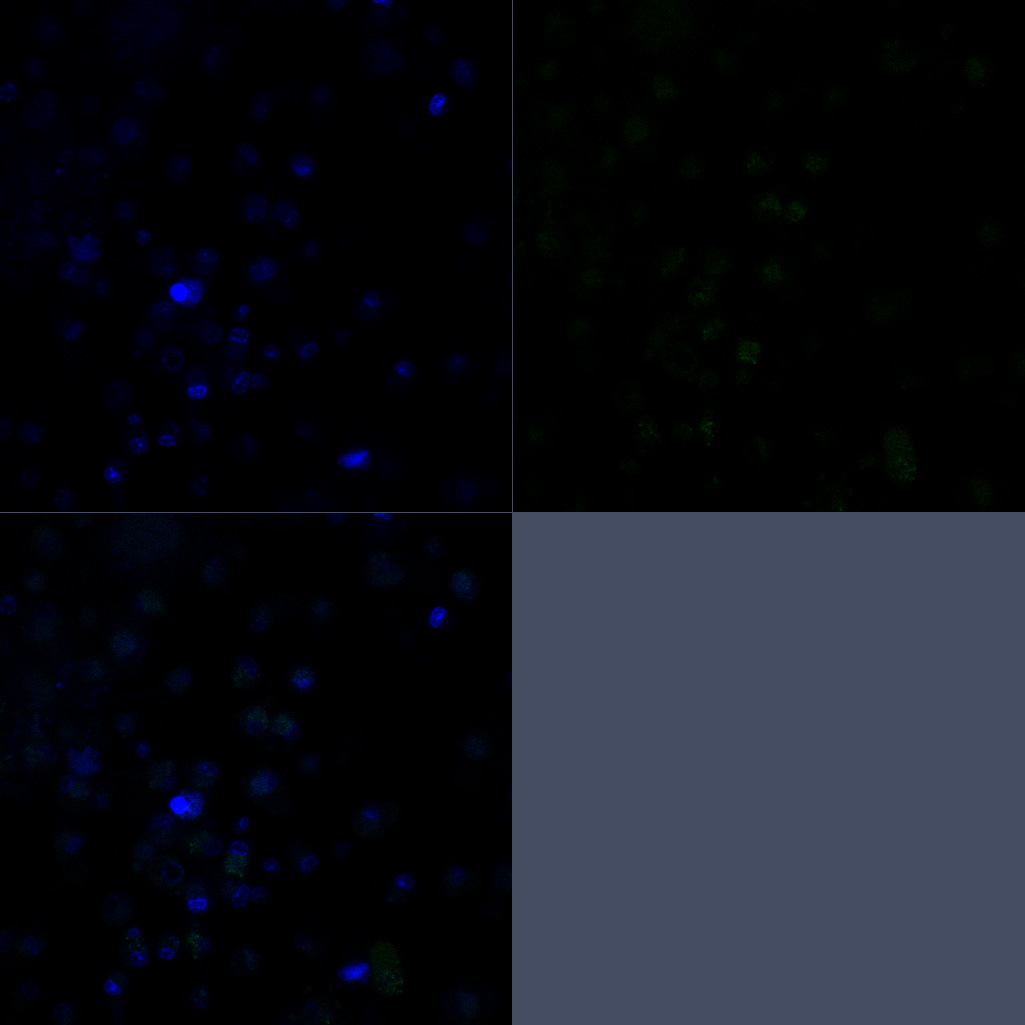

Supplement: Figure 2—source data 6. [file elife-88686-fig2-data6.zip › Figure PBS 63x 3.tif]

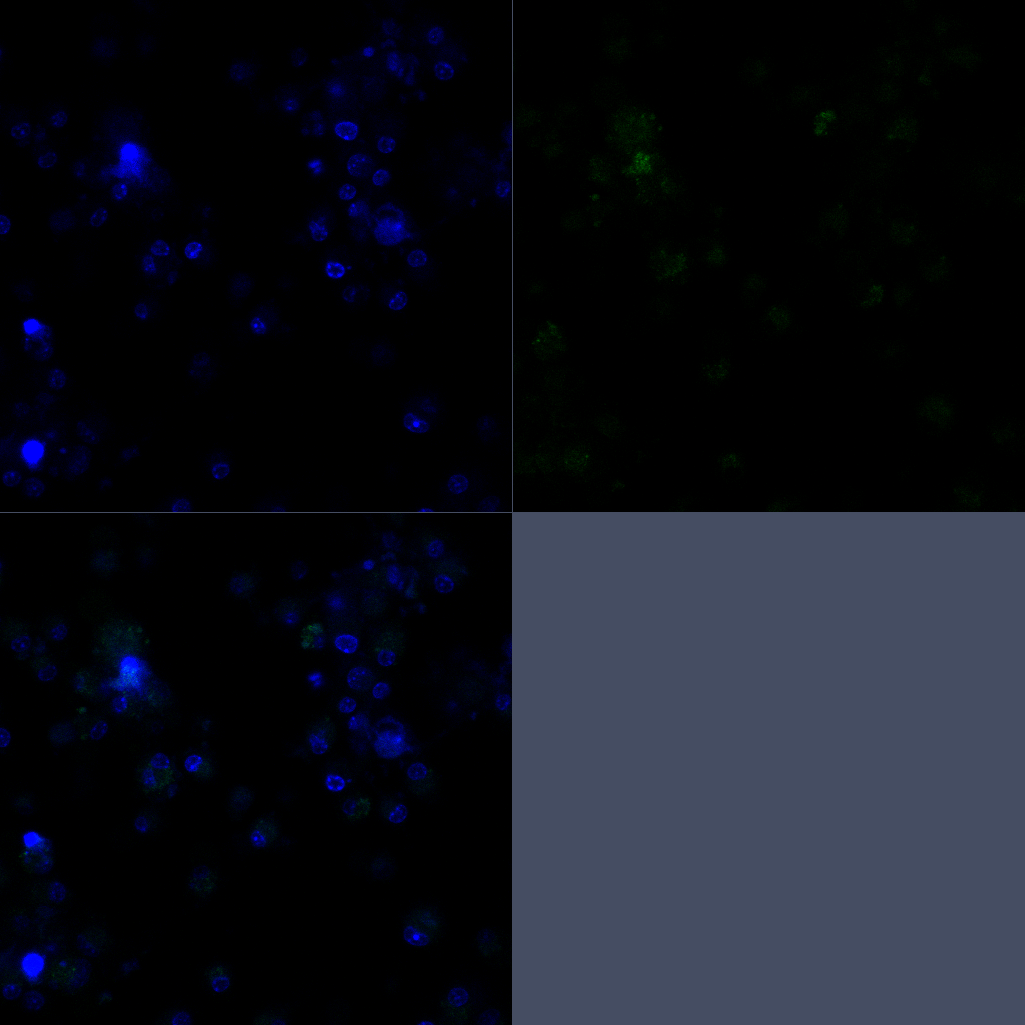

Supplement: Figure 2—source data 6. [file elife-88686-fig2-data6.zip › Figure PBS 63x 4.tif]

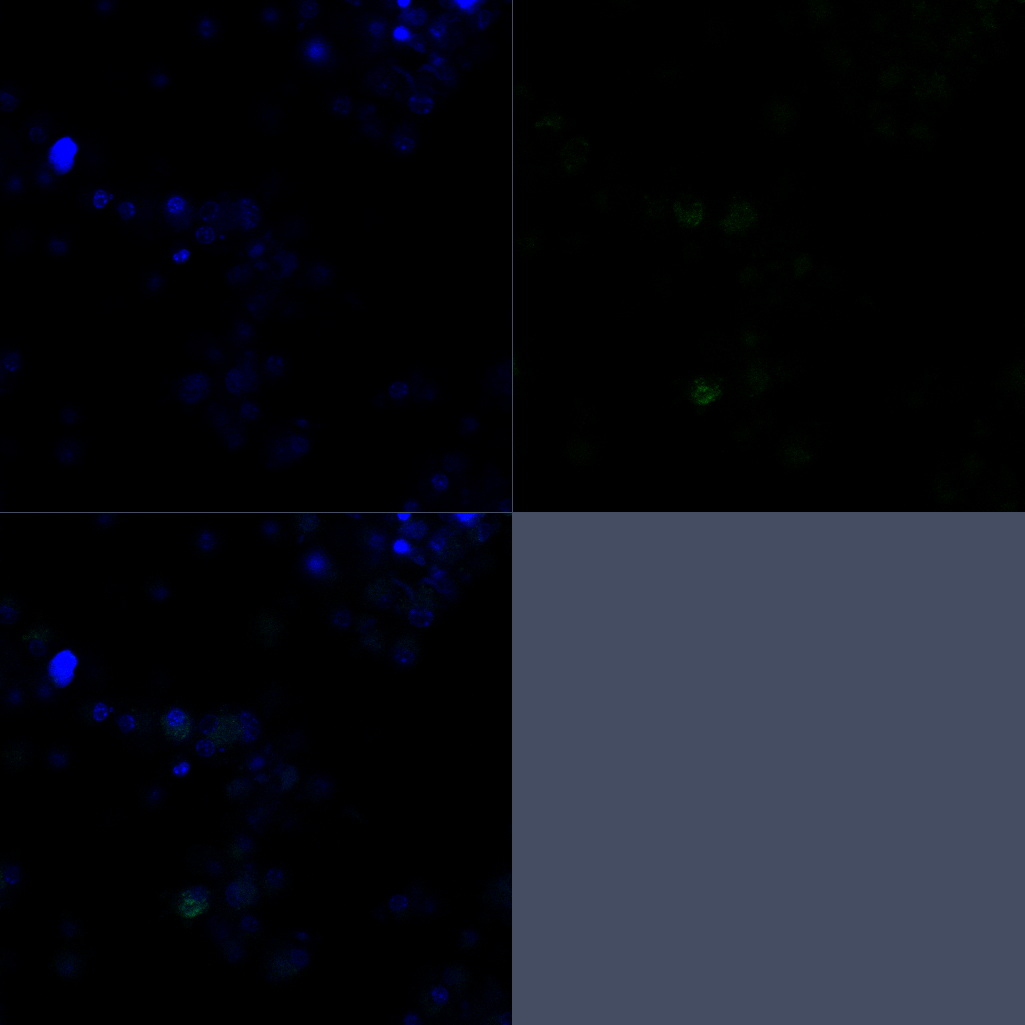

Supplement: Figure 2—source data 6. [file elife-88686-fig2-data6.zip › Figure PBS 63x 5.tif]

p52

Control PBS NAD<sup>+</sup>

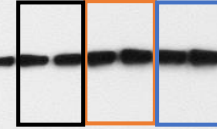

Supplement: Figure 2—figure supplement 1—source data 2. [file elife-88686-fig2-figsupp1-data2.zip › Figure 2 Supplement 2. Nf-kabba B p52 Highlighted.pdf]

# p65

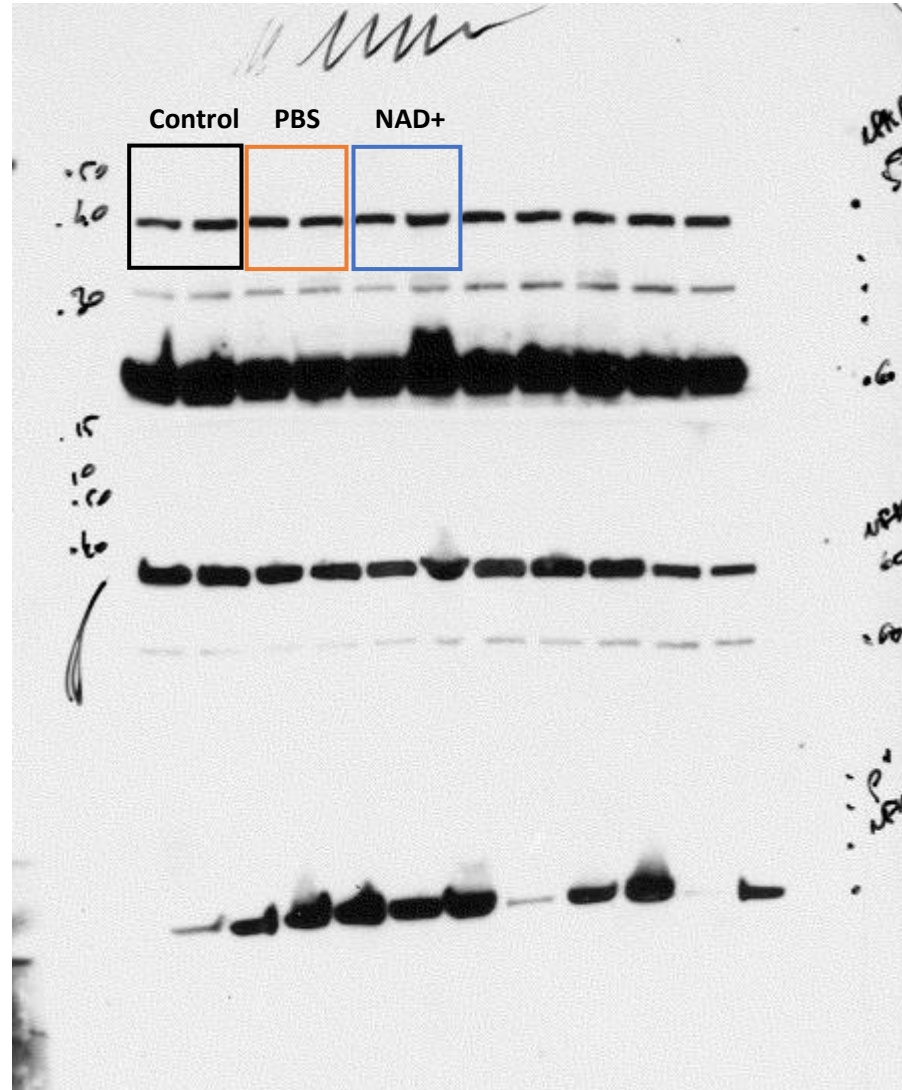

Supplement: Figure 2—figure supplement 1—source data 2. [file elife-88686-fig2-figsupp1-data2.zip › Figure 2 Supplement 2. Nf-kabba B p65 Highlighted.pdf]

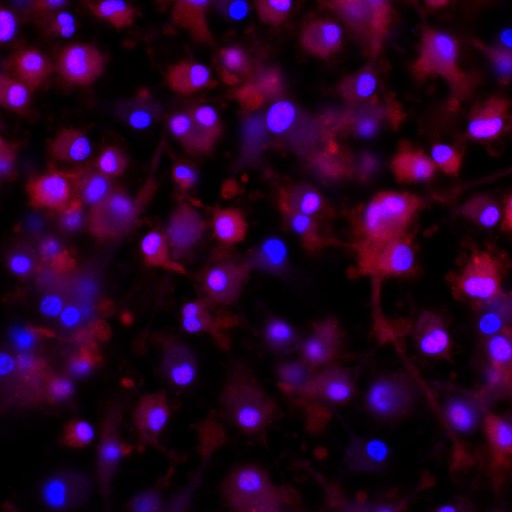

Supplement: Figure 2—figure supplement 1—source data 3. [file elife-88686-fig2-figsupp1-data3.zip › NFKB P65/NAD-NFKB-1.jpg]

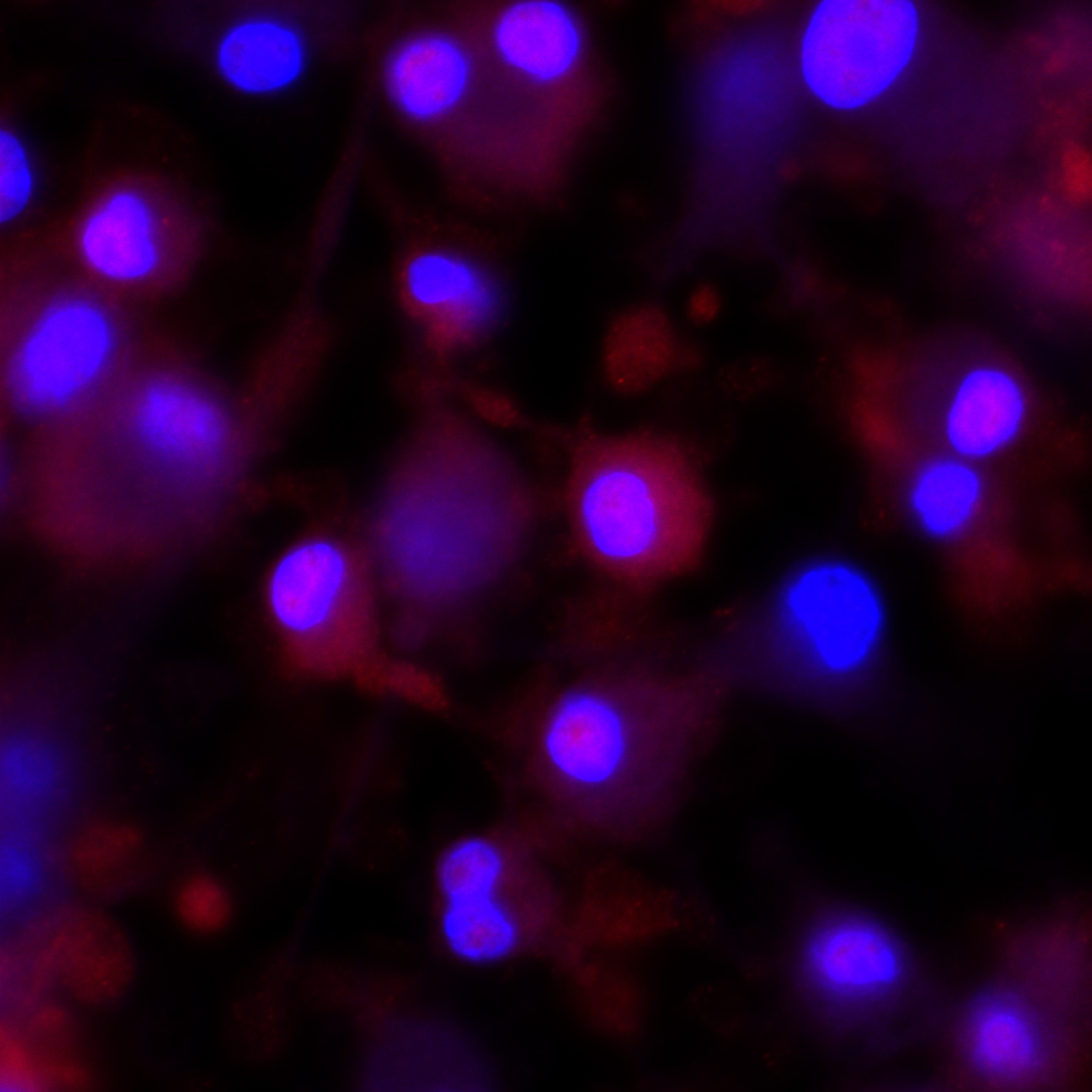

Supplement: Figure 2—figure supplement 1—source data 3. [file elife-88686-fig2-figsupp1-data3.zip › NFKB P65/NAD-NFKB-2.jpg]

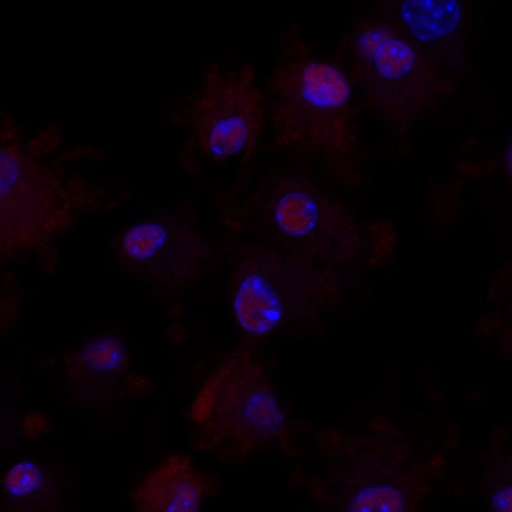

Supplement: Figure 2—figure supplement 1—source data 3. [file elife-88686-fig2-figsupp1-data3.zip › NFKB P65/NAD-NFKB-3.jpg]

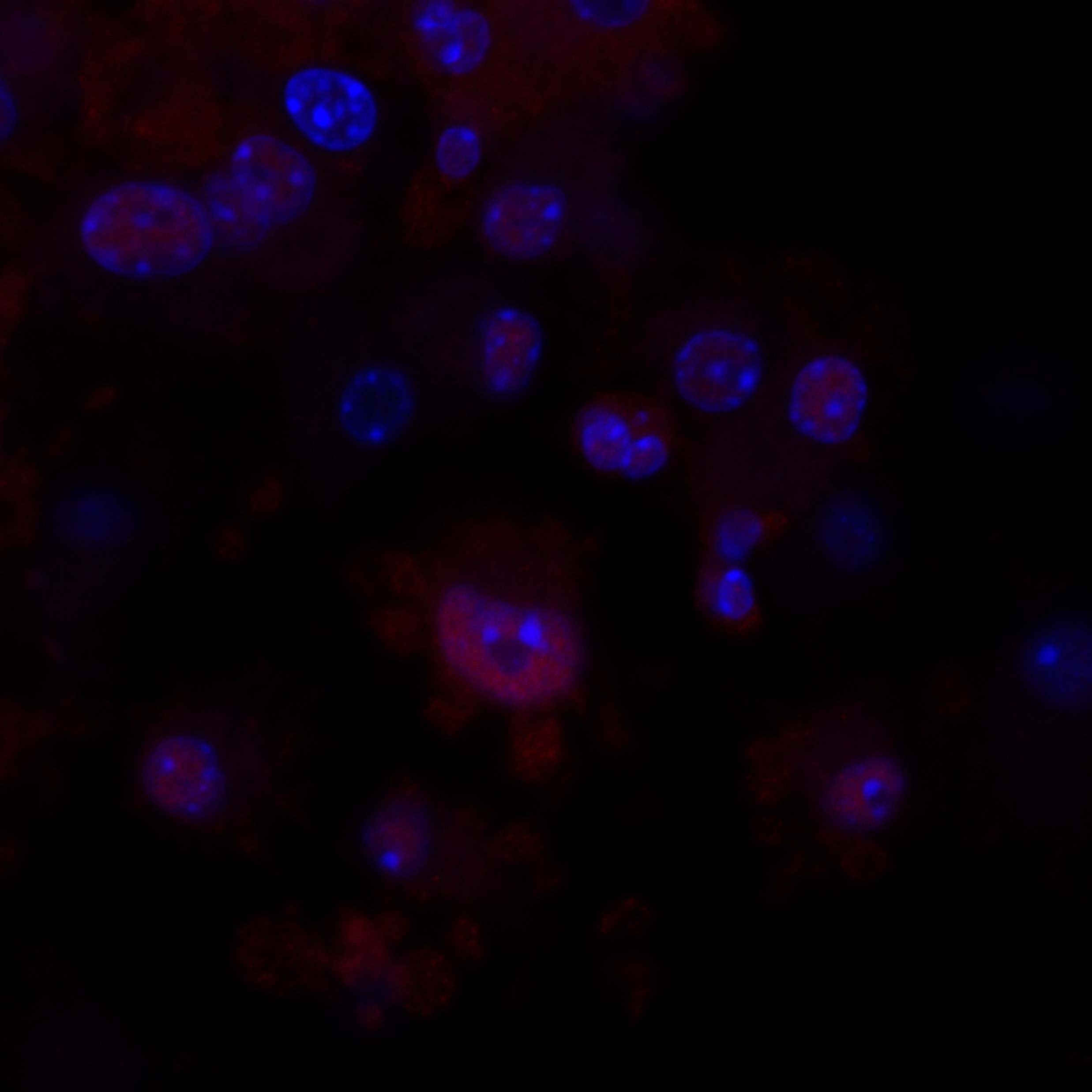

Supplement: Figure 2—figure supplement 1—source data 3. [file elife-88686-fig2-figsupp1-data3.zip › NFKB P65/NAD-NFKB-4.jpg]
