# Supplementary material for: NAD+ prevents septic shock-induced death by non-canonical inflammasome blockade and IL-10 cytokine production in macrophages
Source: eLife. 2024 Feb 19;12:RP88686. doi: 10.7554/eLife.88686 (PMC10942599; doi:10.7554/eLife.88686)
Supplement: Figure 2—source data 1. [file elife-88686-fig2-data1.zip › Figure 2A. Non Canonical Inflammasome NLRP3.pdf]

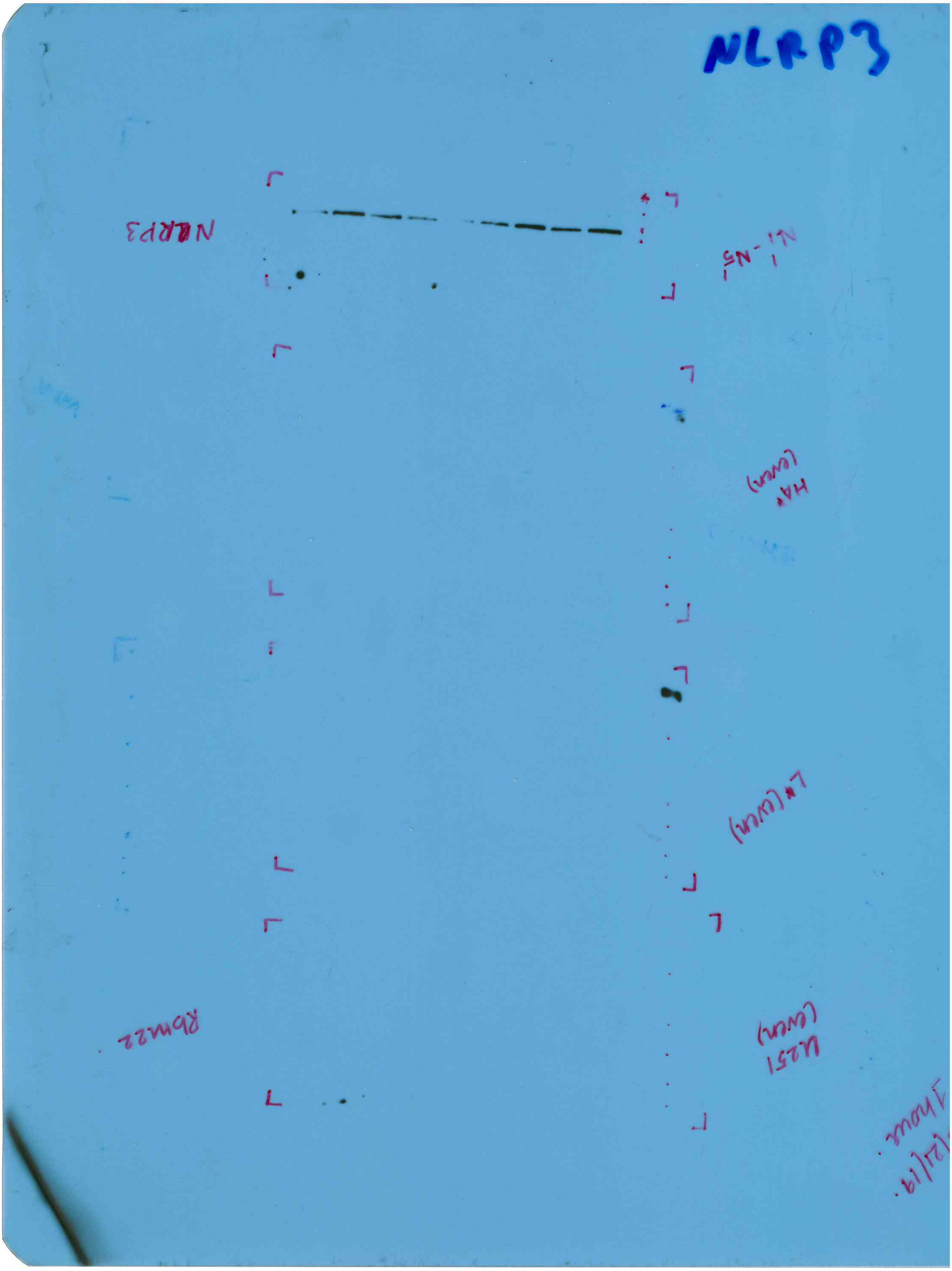
NLRP3
