# Supplementary figures and images for: NAD+ prevents septic shock-induced death by non-canonical inflammasome blockade and IL-10 cytokine production in macrophages (part 2 of 2)
Source: eLife. 2024 Feb 19;12:RP88686. doi: 10.7554/eLife.88686 (PMC10942599; doi:10.7554/eLife.88686)

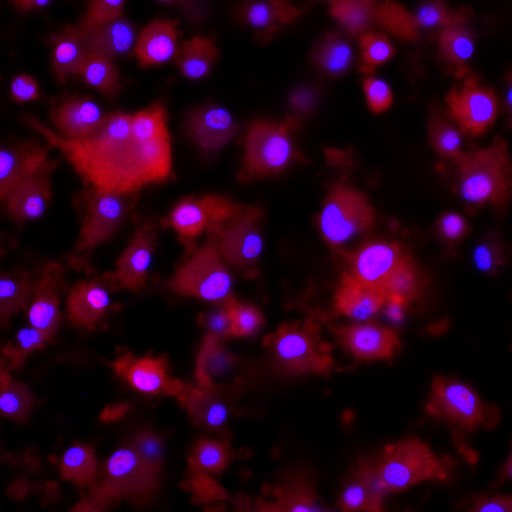

Supplement: Figure 2—figure supplement 1—source data 3. [file elife-88686-fig2-figsupp1-data3.zip › NFKB P65/PBS-NFKB-1.jpg]

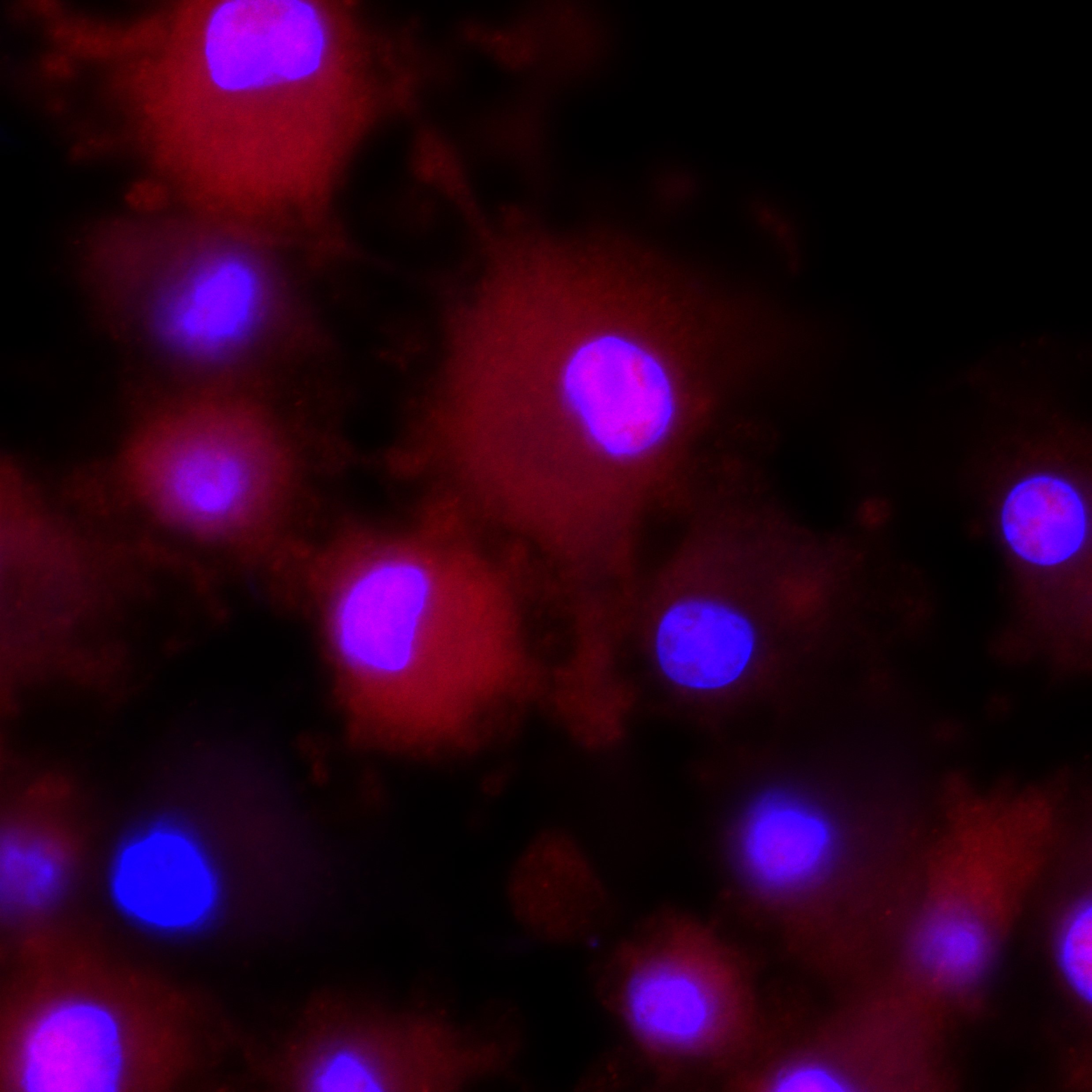

Supplement: Figure 2—figure supplement 1—source data 3. [file elife-88686-fig2-figsupp1-data3.zip › NFKB P65/PBS-NFKB-2.jpg]

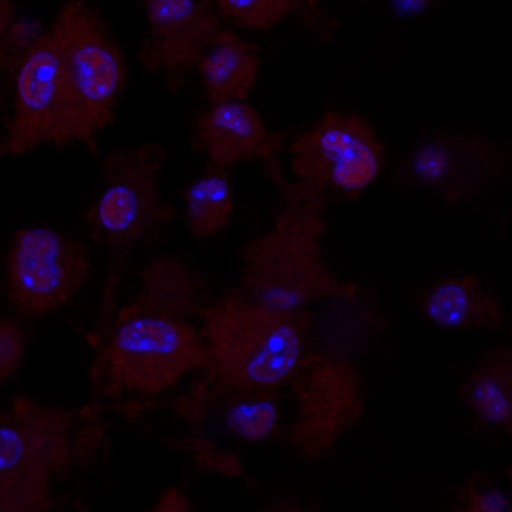

Supplement: Figure 2—figure supplement 1—source data 3. [file elife-88686-fig2-figsupp1-data3.zip › NFKB P65/PBS-NFKB-3.jpg]

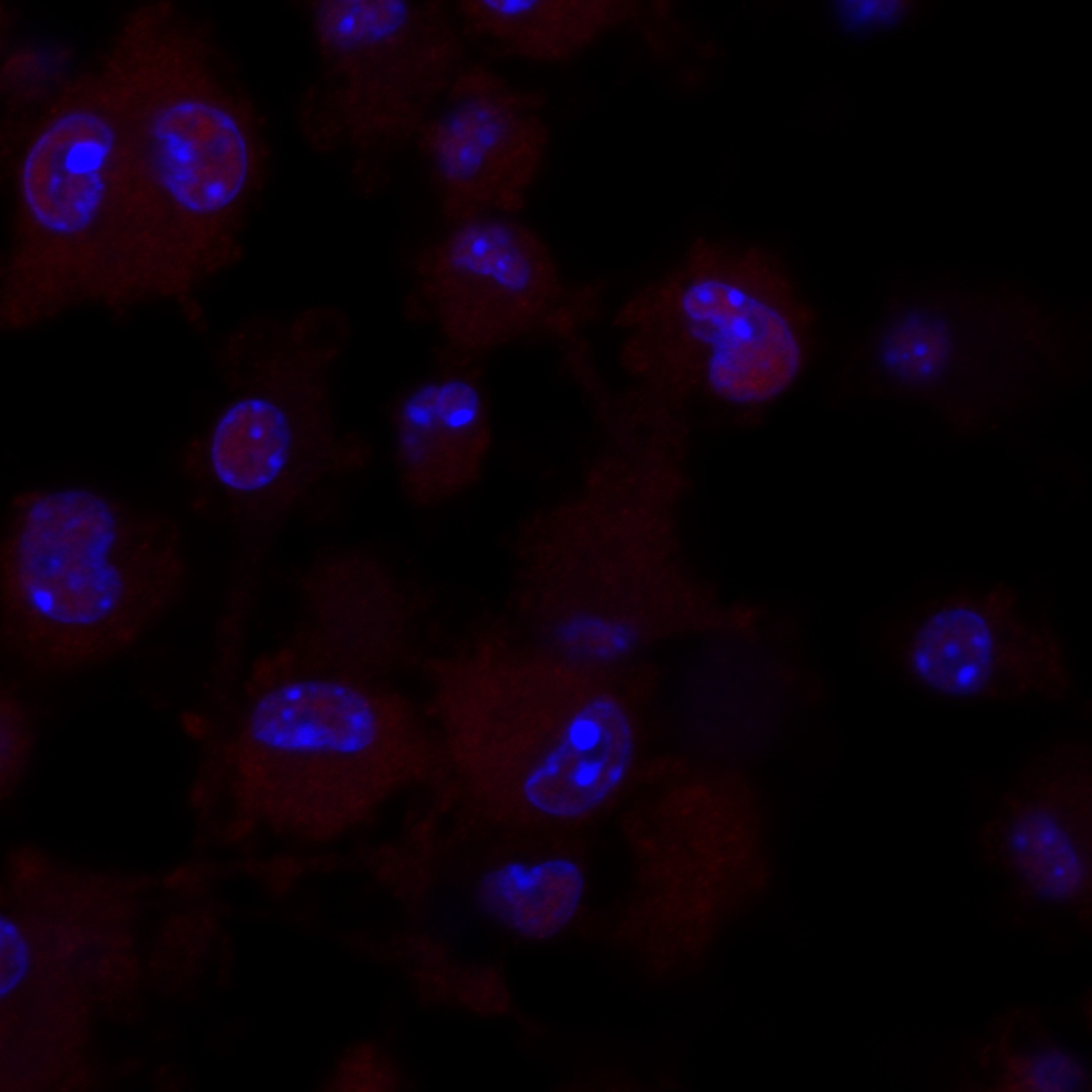

Supplement: Figure 2—figure supplement 1—source data 3. [file elife-88686-fig2-figsupp1-data3.zip › NFKB P65/PBS-NFKB-4.jpg]

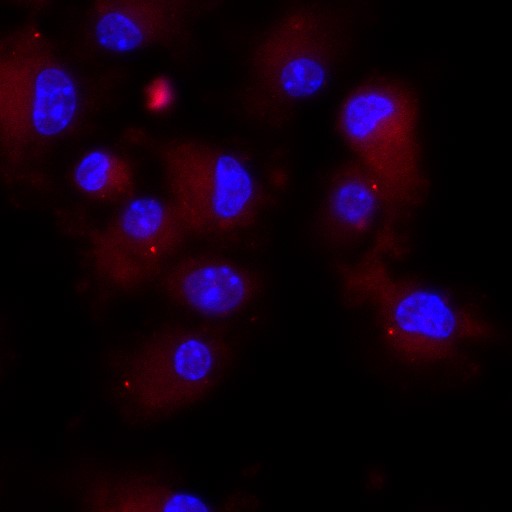

Supplement: Figure 2—figure supplement 1—source data 3. [file elife-88686-fig2-figsupp1-data3.zip › Phospho NFKB-p65/NAD-NFKB.jpg]

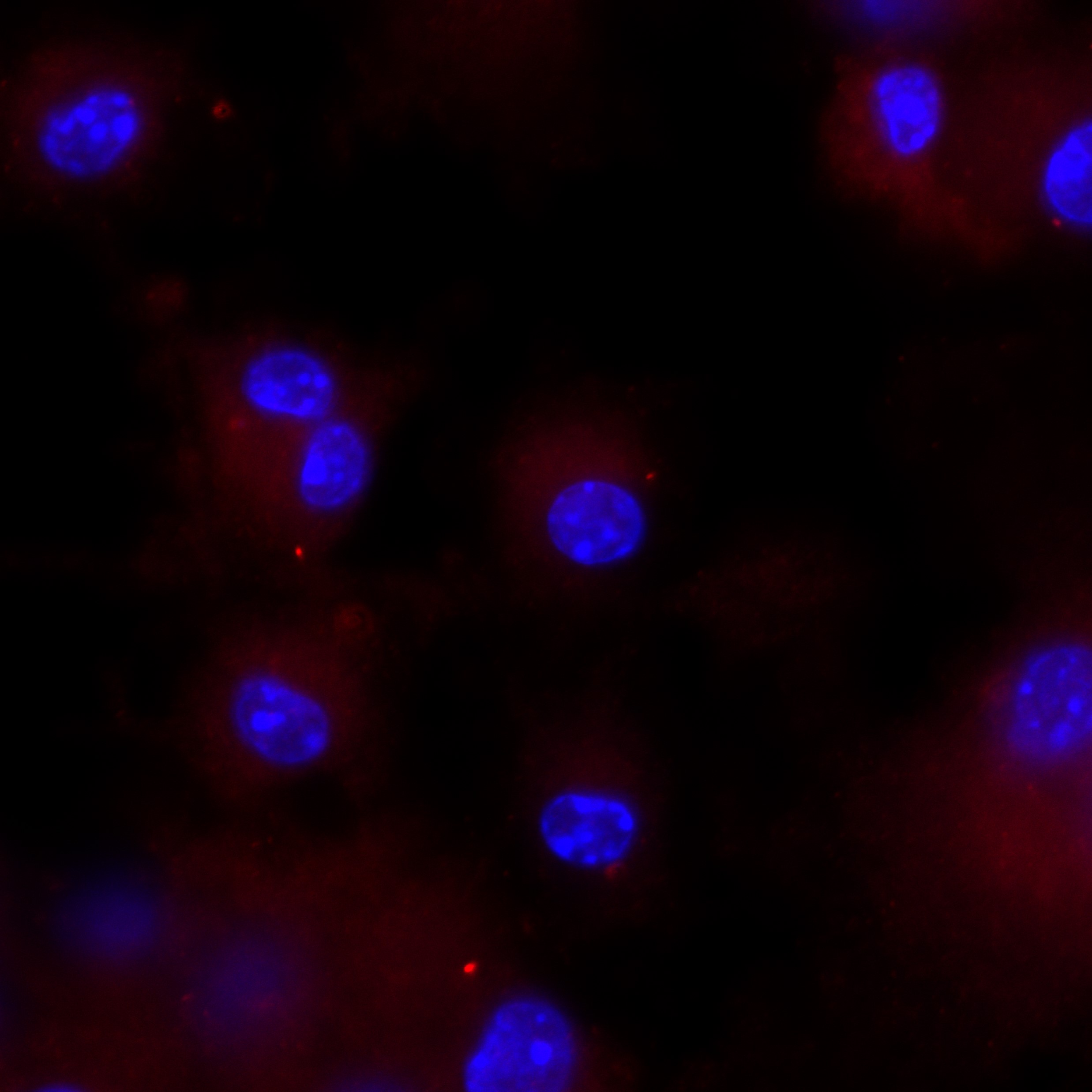

Supplement: Figure 2—figure supplement 1—source data 3. [file elife-88686-fig2-figsupp1-data3.zip › Phospho NFKB-p65/PBS-NFKB.jpg]

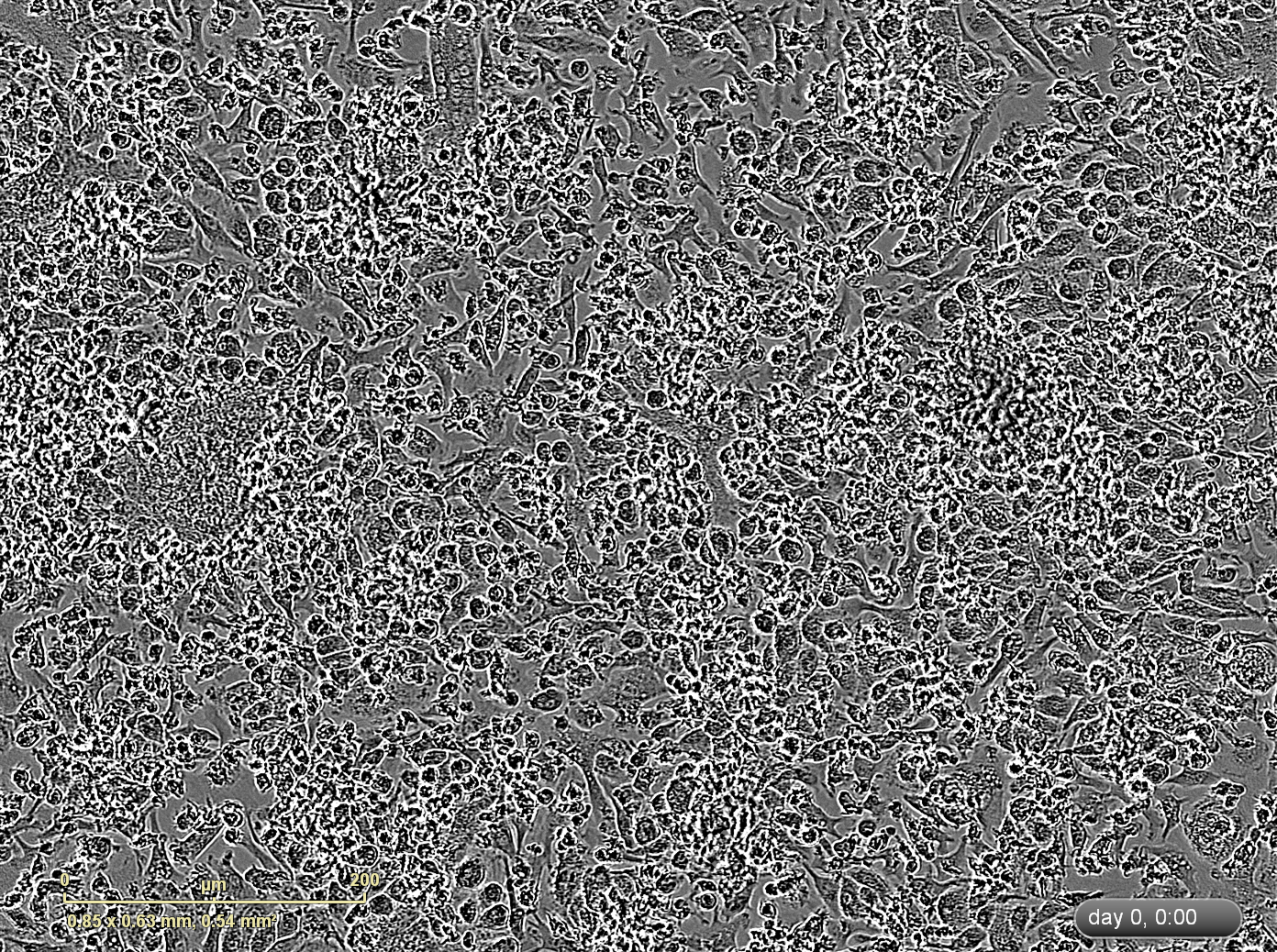

Supplement: Figure 2—figure supplement 2—source data 1. [file elife-88686-fig2-figsupp2-data1.zip › Unstim 0h.tif]

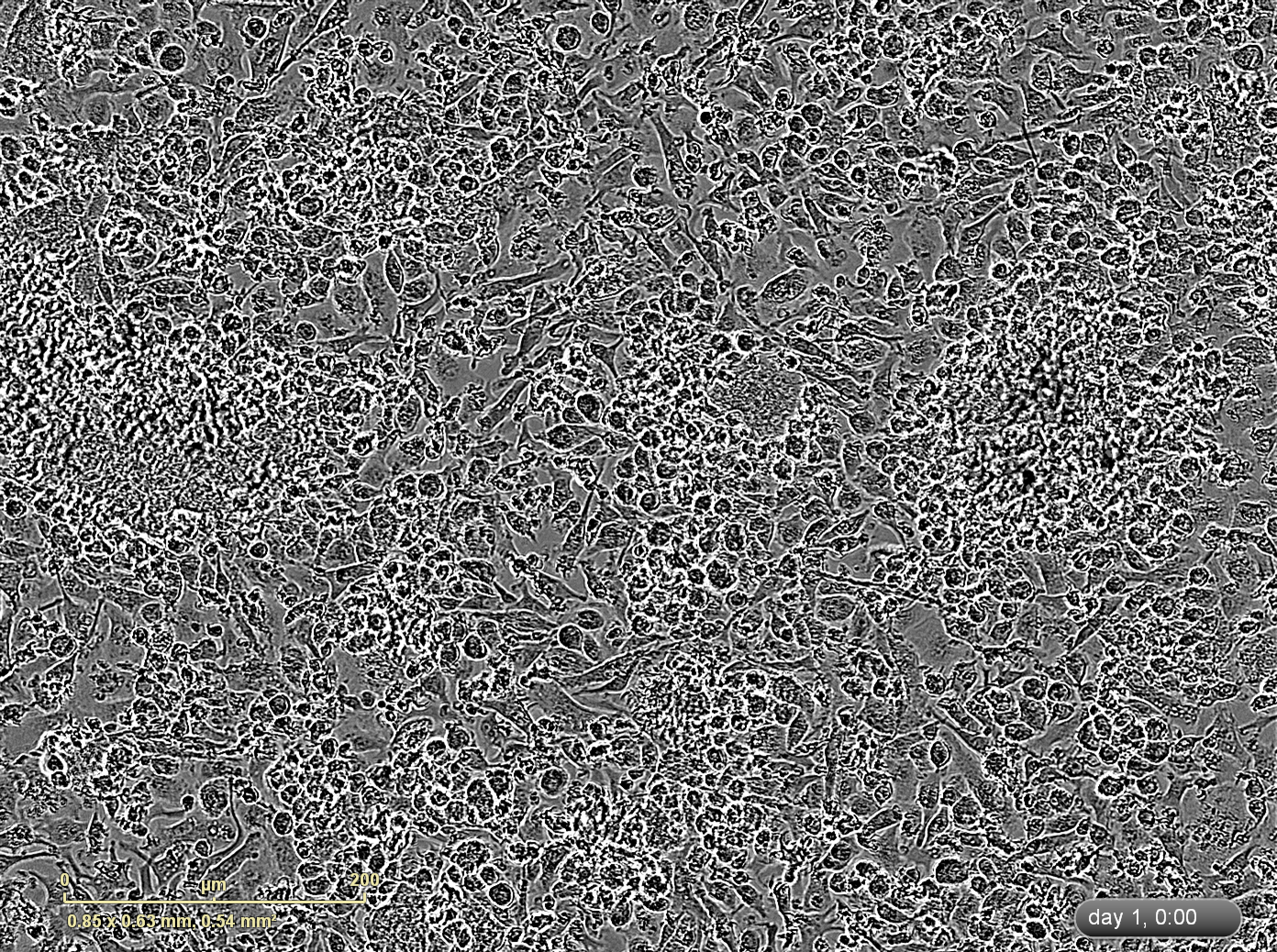

Supplement: Figure 2—figure supplement 2—source data 1. [file elife-88686-fig2-figsupp2-data1.zip › Unstim 24h.tif]

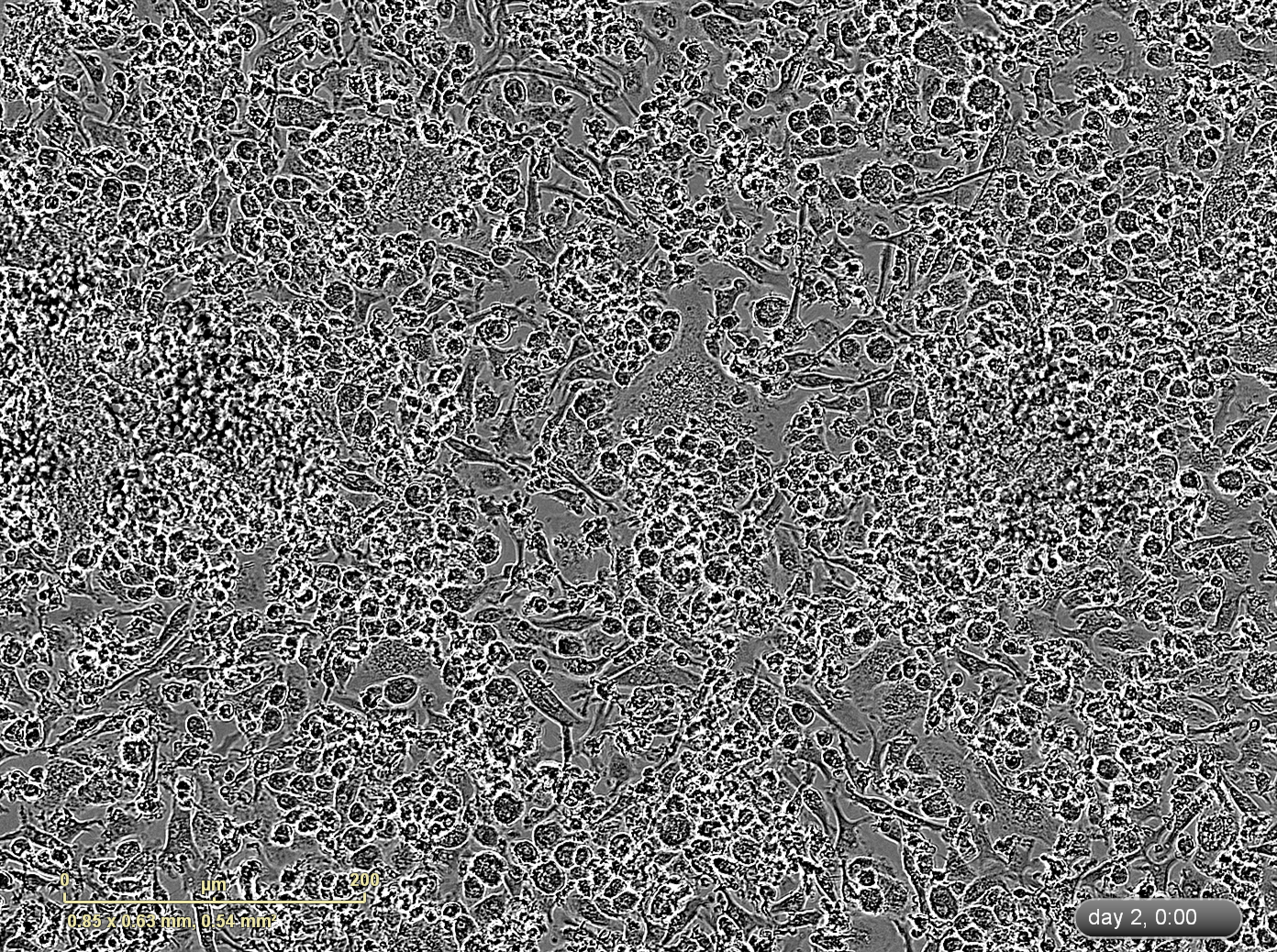

Supplement: Figure 2—figure supplement 2—source data 1. [file elife-88686-fig2-figsupp2-data1.zip › Unstim 48h.tif]

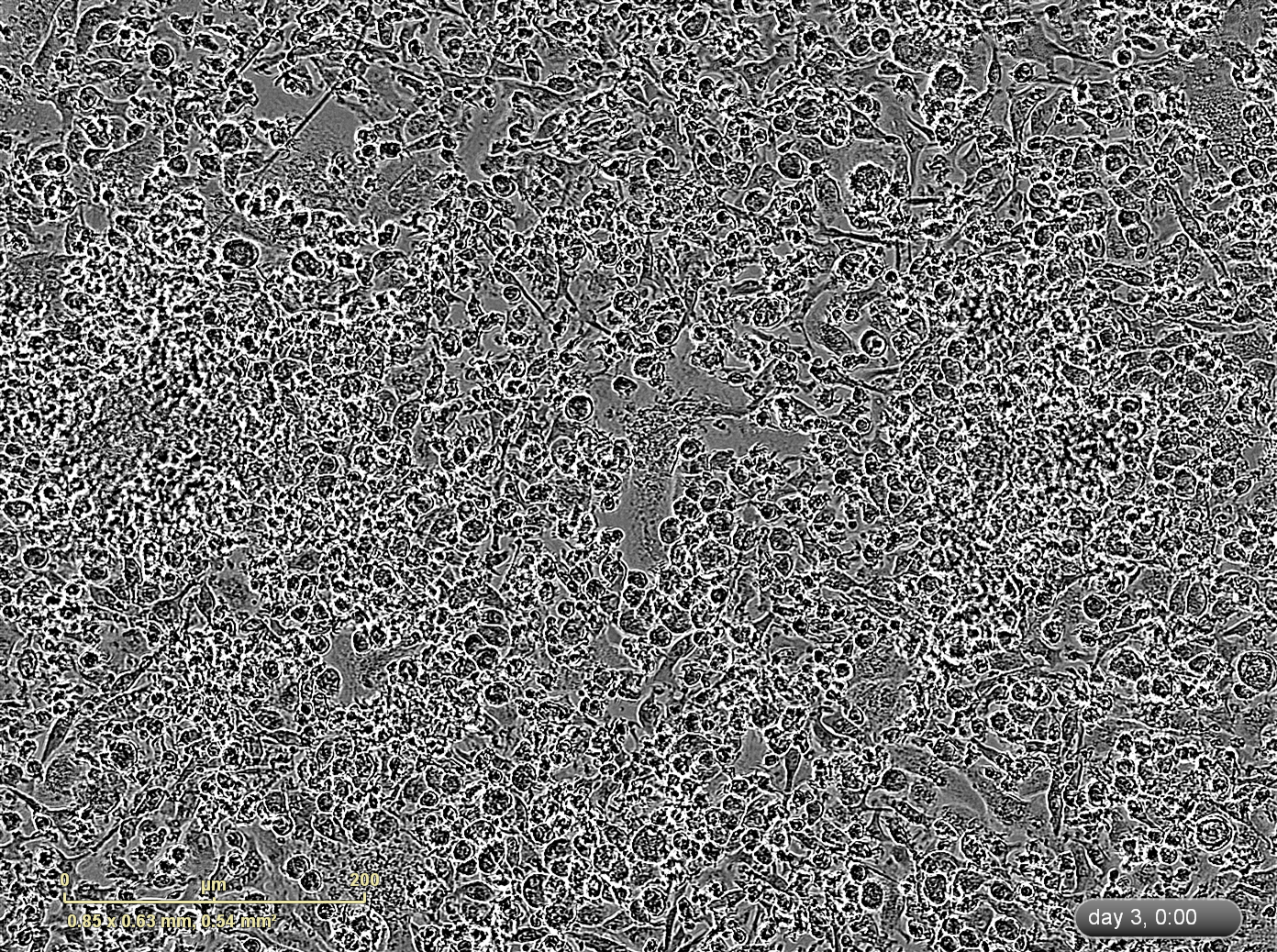

Supplement: Figure 2—figure supplement 2—source data 1. [file elife-88686-fig2-figsupp2-data1.zip › Unstim 72h.tif]

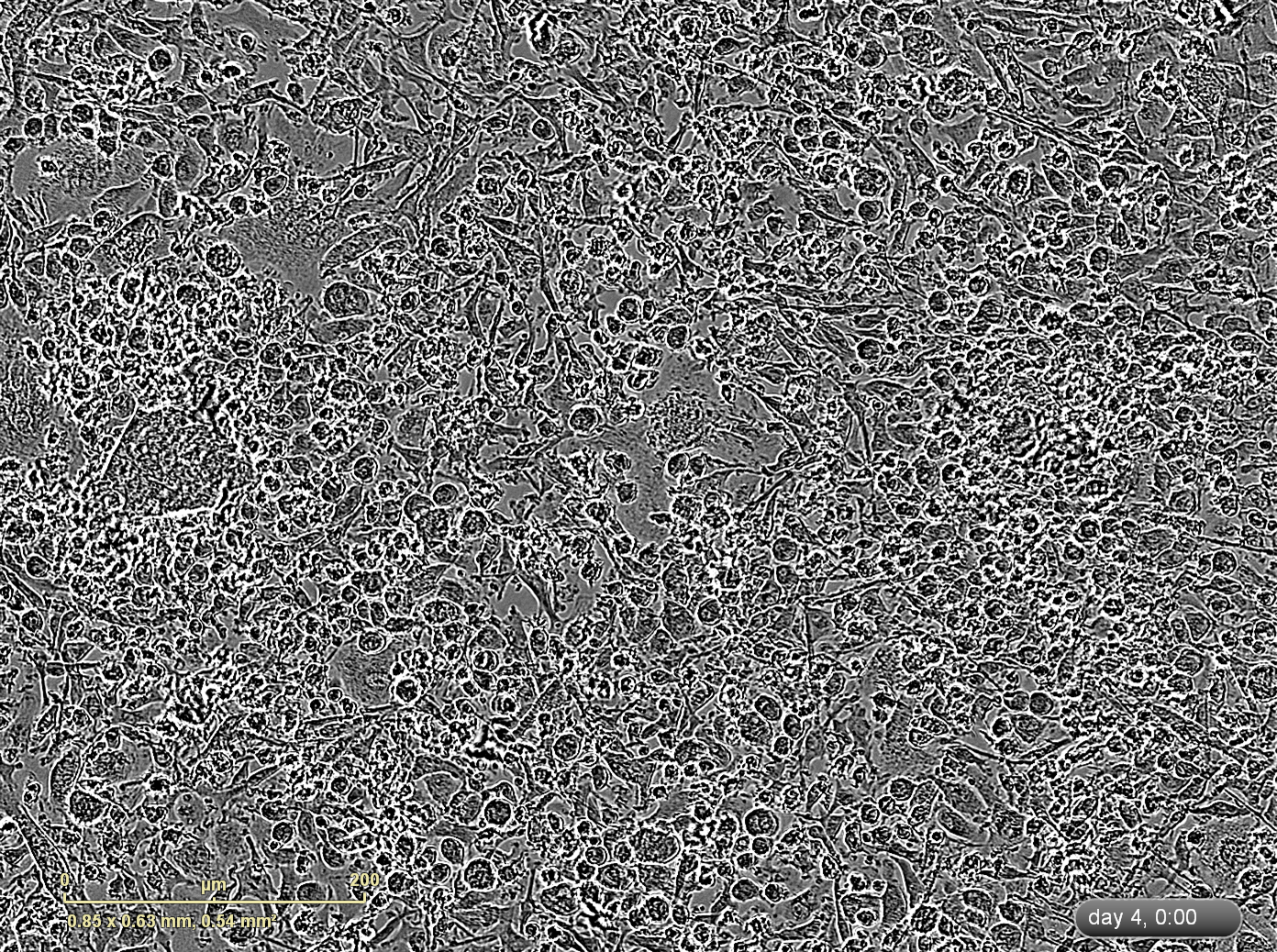

Supplement: Figure 2—figure supplement 2—source data 1. [file elife-88686-fig2-figsupp2-data1.zip › Unstim 96h.tif]

N N N N<sup>1</sup> N<sup>2</sup> N<sup>3</sup> 91 92 93 X X 65040

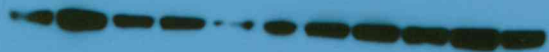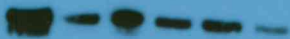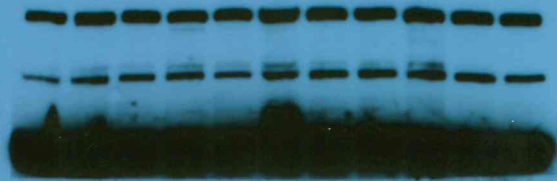

CSP-17

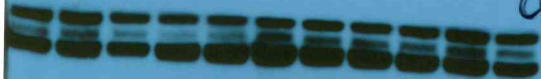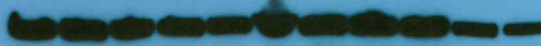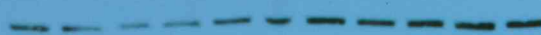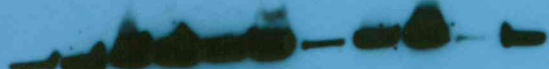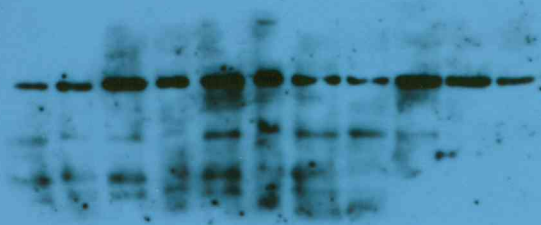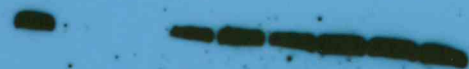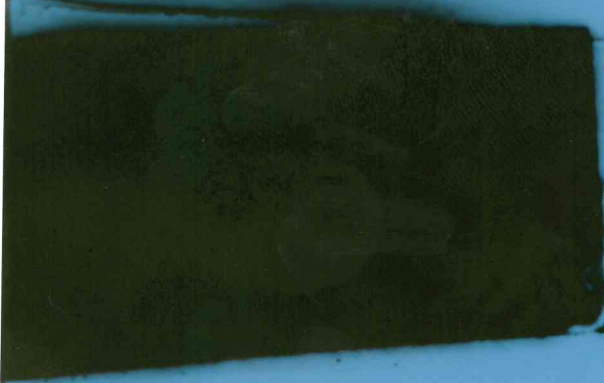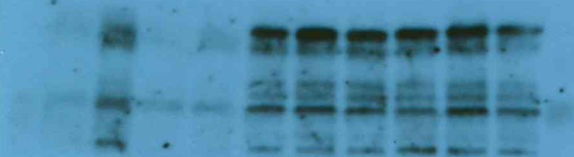

Supplement: Figure 3—source data 2. [file elife-88686-fig3-data2.zip › Figure 3F. Caspase 11 and GSDMD.pdf]

NC 63  
 6-5-79  
 JEN 42

N    W    3    X    N    W    3    X

NC 63

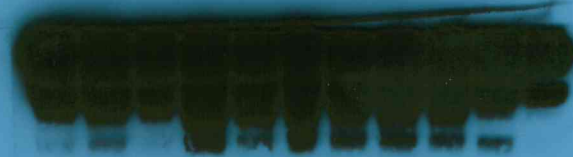

-50  
 -60

Pro 4-12

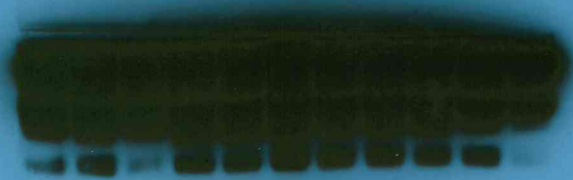

-70  
 -20  
 -10  
 -50  
 -60  
 -30  
 -20  
 -15  
 -10

Pro 4-12

Pro 4-12

Supplement: Figure 3—source data 2. [file elife-88686-fig3-data2.zip › Figure 3F. NLRP3.pdf]

pro-casp-1  
CFL-1

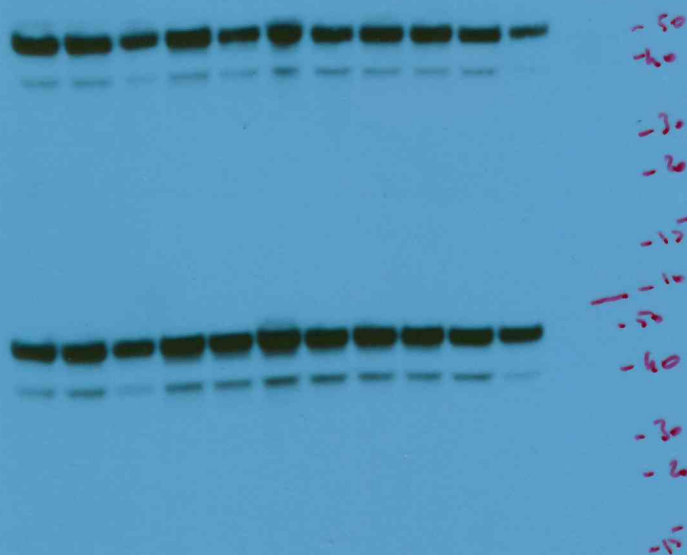

Pro caspase 1

Pro caspase 1

Supplement: Figure 3—source data 2. [file elife-88686-fig3-data2.zip › Figure 3F. Pro-Caspase 1.pdf]

# Actin

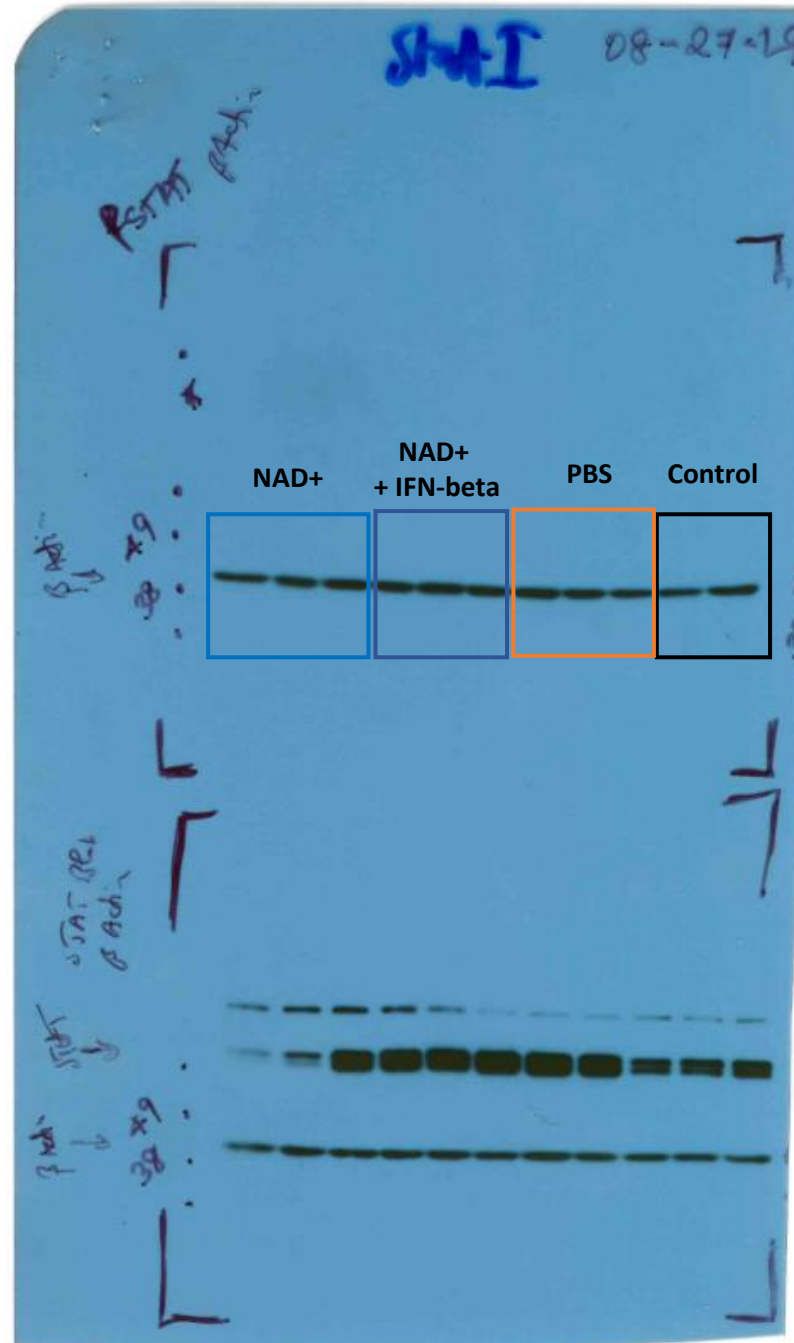

Supplement: Figure 3—source data 3. [file elife-88686-fig3-data3.zip › Figure 3F. Actin Highlighted.pdf]

**GSDMD**

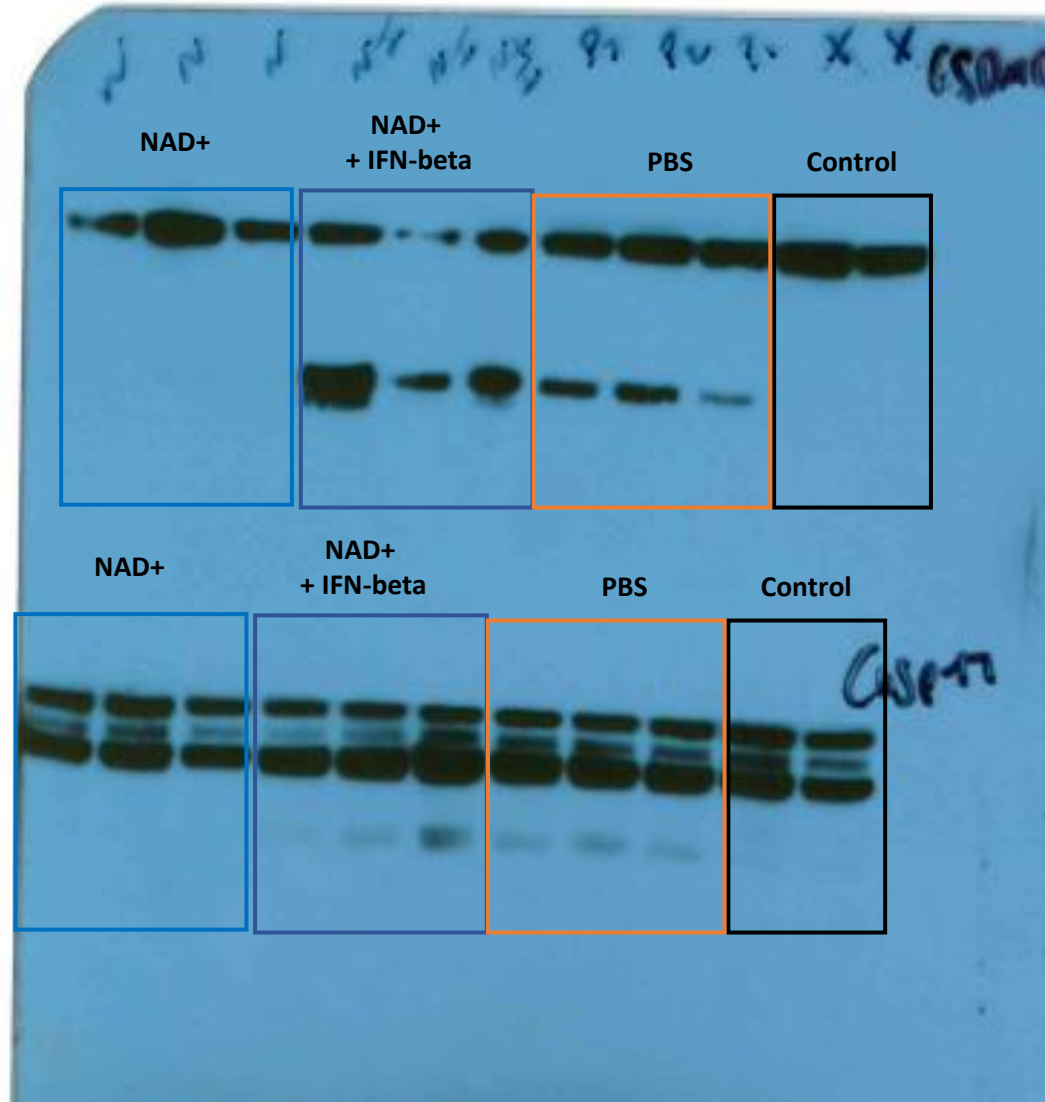

**Casp 11**

Supplement: Figure 3—source data 3. [file elife-88686-fig3-data3.zip › Figure 3F. Caspase 11 and GSDMD Highlighted.pdf]

# NLRP 3

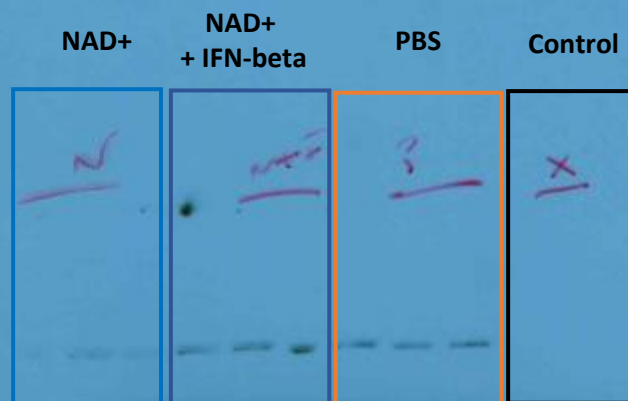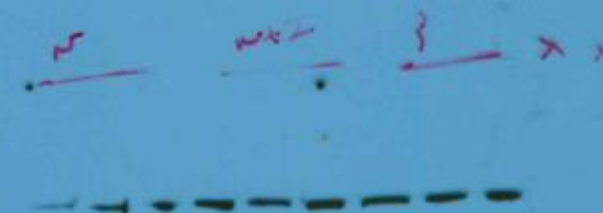

Supplement: Figure 3—source data 3. [file elife-88686-fig3-data3.zip › Figure 3F. NLRP3 Highlighted.pdf]

# Pro Casp 1

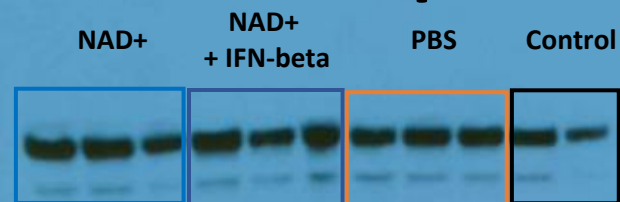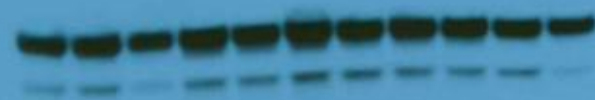

-50  
-40  
-30  
-20  
-15  
-10  
-5  
-40  
-30  
-20  
-15

Pro-casp-1  
CASP-1

Pro caspase 1

Pro caspase 1

Supplement: Figure 3—source data 3. [file elife-88686-fig3-data3.zip › Figure 3F. Pro-Caspase 1 Highlighted.pdf]

Phospho Slot I

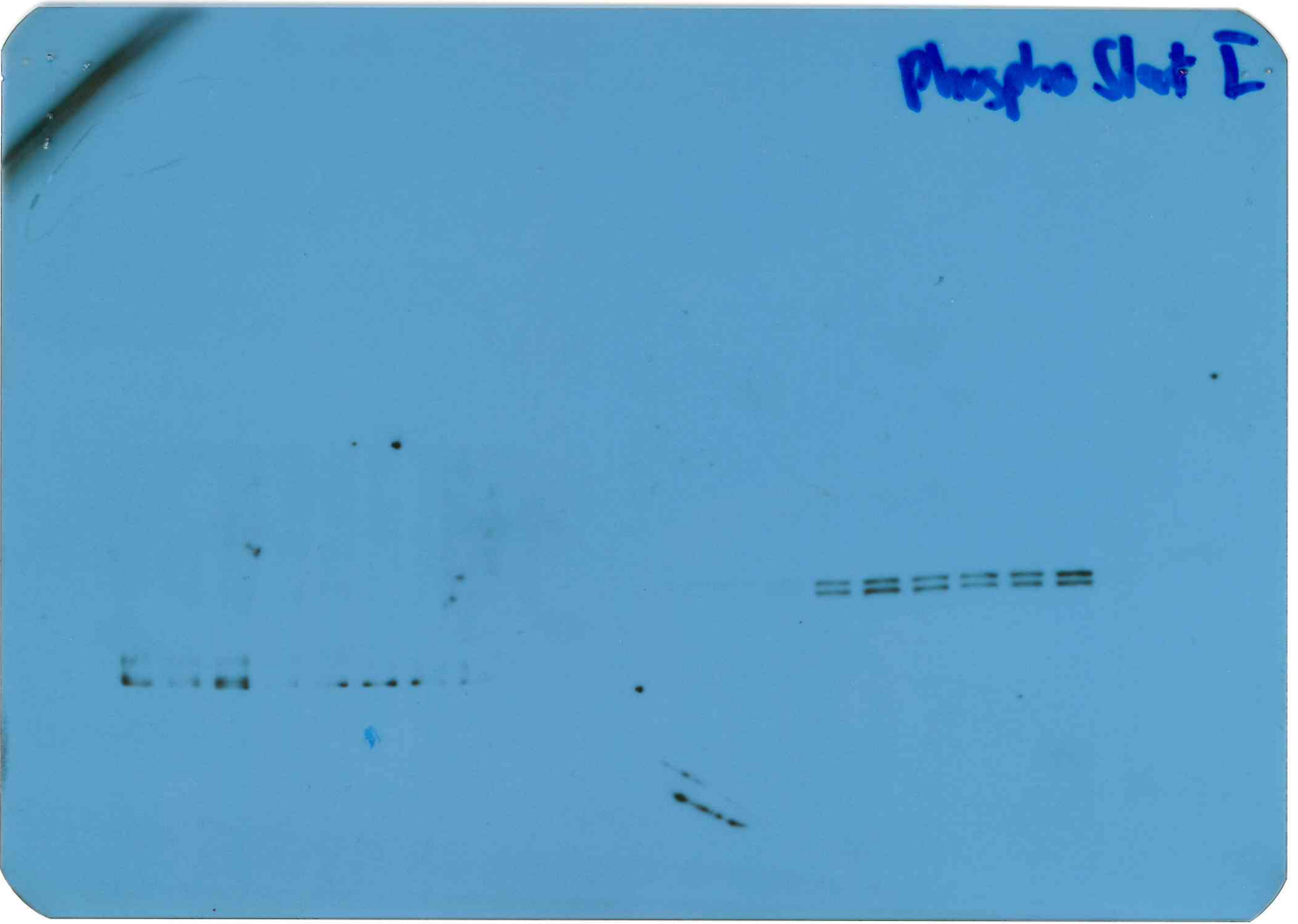

Supplement: Figure 3—source data 4. [file elife-88686-fig3-data4.zip › Figure 3G. Phospho Stat 1.pdf]

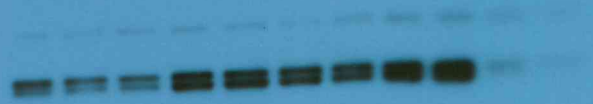

Start [15] + JFN

Supplement: Figure 3—source data 4. [file elife-88686-fig3-data4.zip › Figure 3G. Stat 1.pdf]

Phospho Stat E

STAT1-P

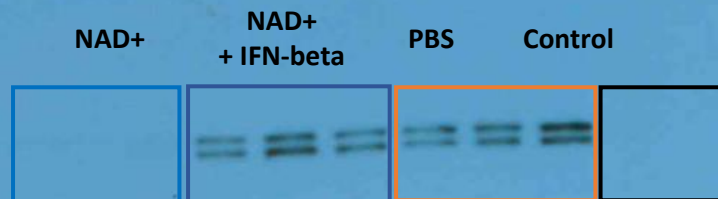

Supplement: Figure 3—source data 5. [file elife-88686-fig3-data5.zip › Figure 3G. Phospho Stat 1 Highlighted.pdf]

# STAT1

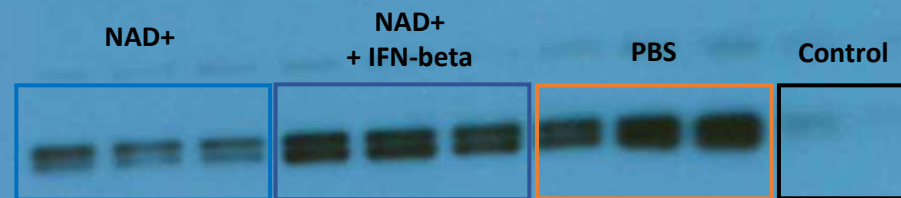

Stat 1 (p) + IFN

Supplement: Figure 3—source data 5. [file elife-88686-fig3-data5.zip › Figure 3G. Stat 1 Highlighted.pdf]
