# Supplementary material for: NAD+ prevents septic shock-induced death by non-canonical inflammasome blockade and IL-10 cytokine production in macrophages
Source: eLife. 2024 Feb 19;12:RP88686. doi: 10.7554/eLife.88686 (PMC10942599; doi:10.7554/eLife.88686)
Supplement: Figure 3—source data 2. [file elife-88686-fig3-data2.zip › Figure 3F. Actin.pdf]

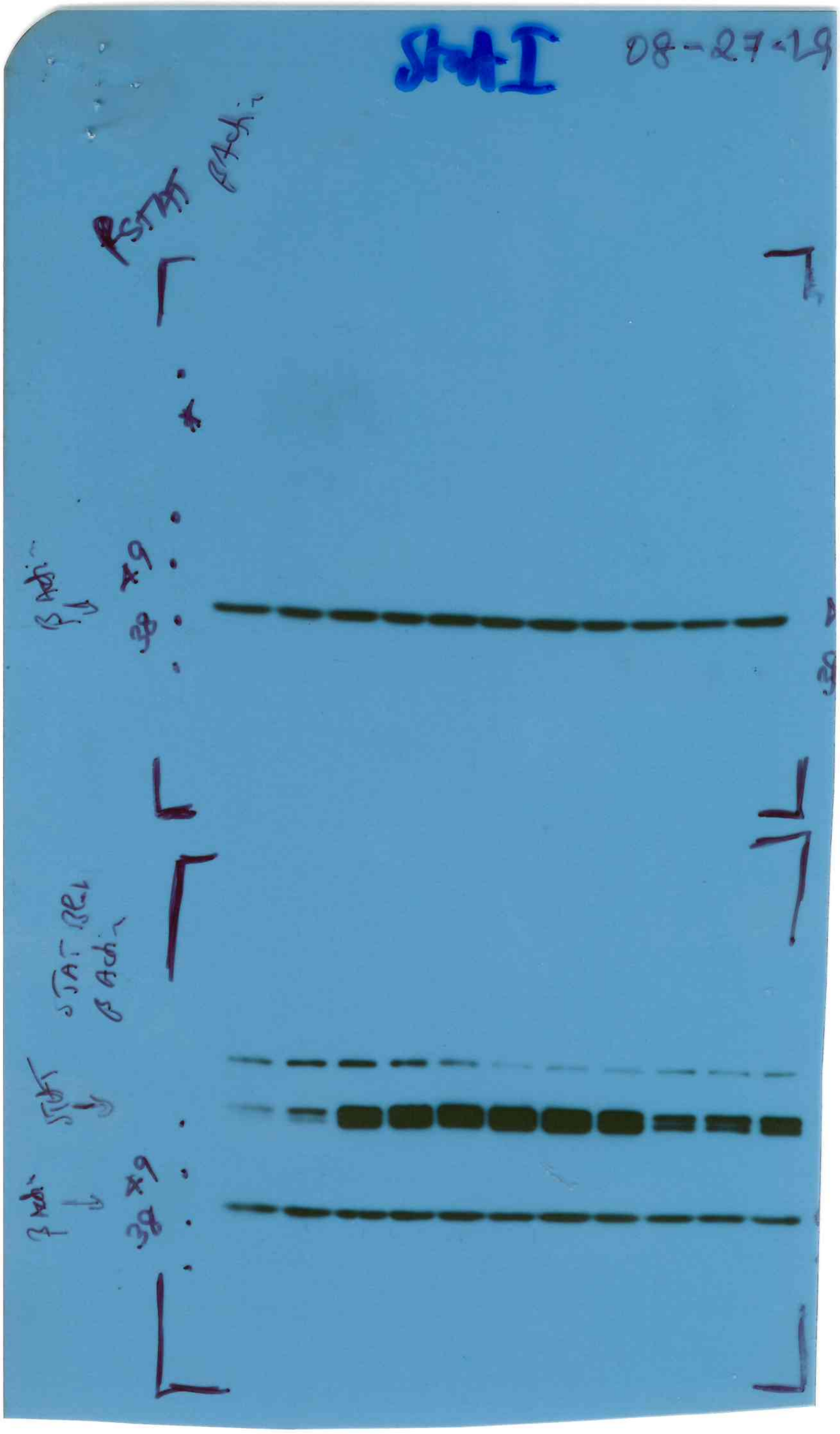

Stat I
08-27-19
